# A comprehensive overview on the crosstalk between microRNAs and viral pathogenesis and infection

**DOI:** 10.1002/med.22073

**Published:** 2024-08-26

**Authors:** Seyedeh Zahra Bahojb Mahdavi, Asiyeh Jebelli, Parisa Shiri Aghbash, Behzad Baradaran, Mohammad Amini, Fatemeh Oroojalian, Nasser Pouladi, Hossein Bannazadeh Baghi, Miguel de la Guardia, Amir Ali Mokhtarzadeh

**Affiliations:** ^1^ Department of Biology, Faculty of Basic Sciences Azarbaijan Shahid Madani University Tabriz Iran; ^2^ Immunology Research Center Tabriz University of Medical Sciences Tabriz Iran; ^3^ Department of Biological Science, Faculty of Basic Science Higher Education Institute of Rab‐Rashid Tabriz Iran; ^4^ Tuberculosis and Lung Diseases Research Center Tabriz University of Medical Sciences Tabriz Iran; ^5^ Department of Advanced Sciences and Technologies in Medicine, School of Medicine North Khorasan University of Medical Sciences Bojnurd Iran; ^6^ Department of Virology, Faculty of Medicine Tabriz University of Medical Sciences Tabriz Iran; ^7^ Department of Analytical Chemistry University of Valencia Burjassot Valencia Spain

**Keywords:** coronavirus, hepatitis B, hepatitis C, host miRNAs, human immunodeficiency virus, human papillomavirus, influenza A virus, viral miRNAs, virus

## Abstract

Infections caused by viruses as the smallest infectious agents, pose a major threat to global public health. Viral infections utilize different host mechanisms to facilitate their own propagation and pathogenesis. MicroRNAs (miRNAs), as small noncoding RNA molecules, play important regulatory roles in different diseases, including viral infections. They can promote or inhibit viral infection and have a pro‐viral or antiviral role. Also, viral infections can modulate the expression of host miRNAs. Furthermore, viruses from different families evade the host immune response by producing their own miRNAs called viral miRNAs (v‐miRNAs). Understanding the replication cycle of viruses and their relation with host miRNAs and v‐miRNAs can help to find new treatments against viral infections. In this review, we aim to outline the structure, genome, and replication cycle of various viruses including hepatitis B, hepatitis C, influenza A virus, coronavirus, human immunodeficiency virus, human papillomavirus, herpes simplex virus, Epstein–Barr virus, Dengue virus, Zika virus, and Ebola virus. We also discuss the role of different host miRNAs and v‐miRNAs and their role in the pathogenesis of these viral infections.

## 1 INTRODUCTION

The human genome project ascertained that only about 2% of the human genome encodes proteins, whereas around 98% of the genome does not have any information for protein coding. These parts of the genome produce RNA transcripts called noncoding RNAs which are classified into two groups: long noncoding RNAs with more than 200 nucleotides in length and small noncoding RNAs with less than 200 nucleotides.^1^, ^2^ MicroRNAs (miRNAs), as single‐stranded (ss) RNAs with a length of 17–24 nucleotides, are a member of the small noncoding RNAs family. The main role of miRNAs is the regulation of gene expression and translational inhibition of mature mRNAs by binding to the 3′ untranslated region (3′‐UTR).^3^, ^4^, ^5^ However, some miRNAs can induce translation upregulation. For instance, translation is repressed by hsa‐miR‐let7 in proliferating cells, whereas translation of target mRNAs is upregulated by hsa‐miR‐let‐7 in cell cycle arrest.^6^ Approximately 1881 miRNA sequences generate over 2588 mature miRNAs, according to miRBase 21.0.^7^, ^8^ These molecules were first described in 1993 by Lee et al., and since then, many studies have been conducted on the importance of these noncoding RNAs.^9^, ^10^ miRNAs have crucial roles in extensive number of biological processes including development, immune function, cell death, and regulation of antiviral host defense by mediating gene expression.^11^, ^12^, ^13^

Viral infections constitute a worldwide challenge for the world's population and play a remarkable role in pandemics and epidemics by affecting millions of people worldwide. Viruses, as the smallest intracellular parasites and major players in viral infections, utilize host cellular machinery for their genome replication, translation, and propagation to survive and produce new viral particles. These microscopic parasites are classified into different groups by their family, genome, or shape. Some viruses can stay in latent state, and in some conditions, can cause malignant or nonmalignant diseases that are responsible for human death. They can affect host cells in different manners by controlling host gene expression and regulating the expression of host miRNAs, which can result in the alteration of miRNA expression profiles.^14^ Viral infections, also, function as triggers for the expression of antiviral miRNAs.^15^, ^16^

Viruses from different families encode their own miRNAs called viral miRNAs (v‐miRNAs), which can operate as posttranscriptional gene regulators in host cells and participate in viral replication cycle.^17^ Similar to eukaryotic miRNAs, Dicer and DROSHA are responsible for v‐miRNAs synthesis.^18^ Some viruses are capable of encoding their own miRNAs; however, others cannot encode v‐miRNAs. The pattern of v‐miRNA expression remains unknown. However, there are some reasons that could account for this. DNA viruses are the most common source of viral miRNAs. miRNA biogenesis machinery is in the nucleus; cytoplasmic localization of RNA viruses prevents access of viral RNA genomes to nuclear miRNA processing machinery, which could be the reason of RNA viruses avoiding encoding their own miRNAs. Another reason could be to avoid genome instability. Cleavage and degradation induced by excision of encoded pre‐miRNA can lead to instability in viruses and the reason for avoiding encoding their own miRNAs.^19^, ^20^ However, a number of reports have suggested that HIV, as an RNA virus, encodes miRNAs as well.^21^ More studies are needed to determine why some viruses encode their own miRNAs while others do not.

Host miRNAs play an essential role in the interplay between hosts and viruses, from virus attachment to propagation and replication. They perform these functions by inhibiting or facilitating viral infections with specific regulatory pathways, which can be conducted directly by binding of host miRNAs to the viral genome or indirectly through targeting of replication related host factors.^8^, ^22^ In this review, we will discuss the involvement of specific miRNAs during different viral infections, and we will focus on the role of host‐encoded miRNAs in the regulation of viral replication and viral pathogenesis. Understanding these miRNAs functions in different viral infections can lead to development of novel methods for viral infection diagnosis and treatment.

## 2 miRNA BIOGENESIS

The miRNA genes are encoded by some specific clusters or individual genes and are classified into two different categories based on their genomic position: (I) intragenic and (II) intergenic. Intragenic miRNAs are coregulated with their host genes by function of RNA polymerase (Pol) II and these genes are embedded within exons or introns of protein‐coding or noncoding genes of same strand genes. Whereas intergenic miRNAs are located between genes and their transcription is regulated by RNA their own Pol II or Pol III promoters.^23^, ^24^

**Figure 1:**
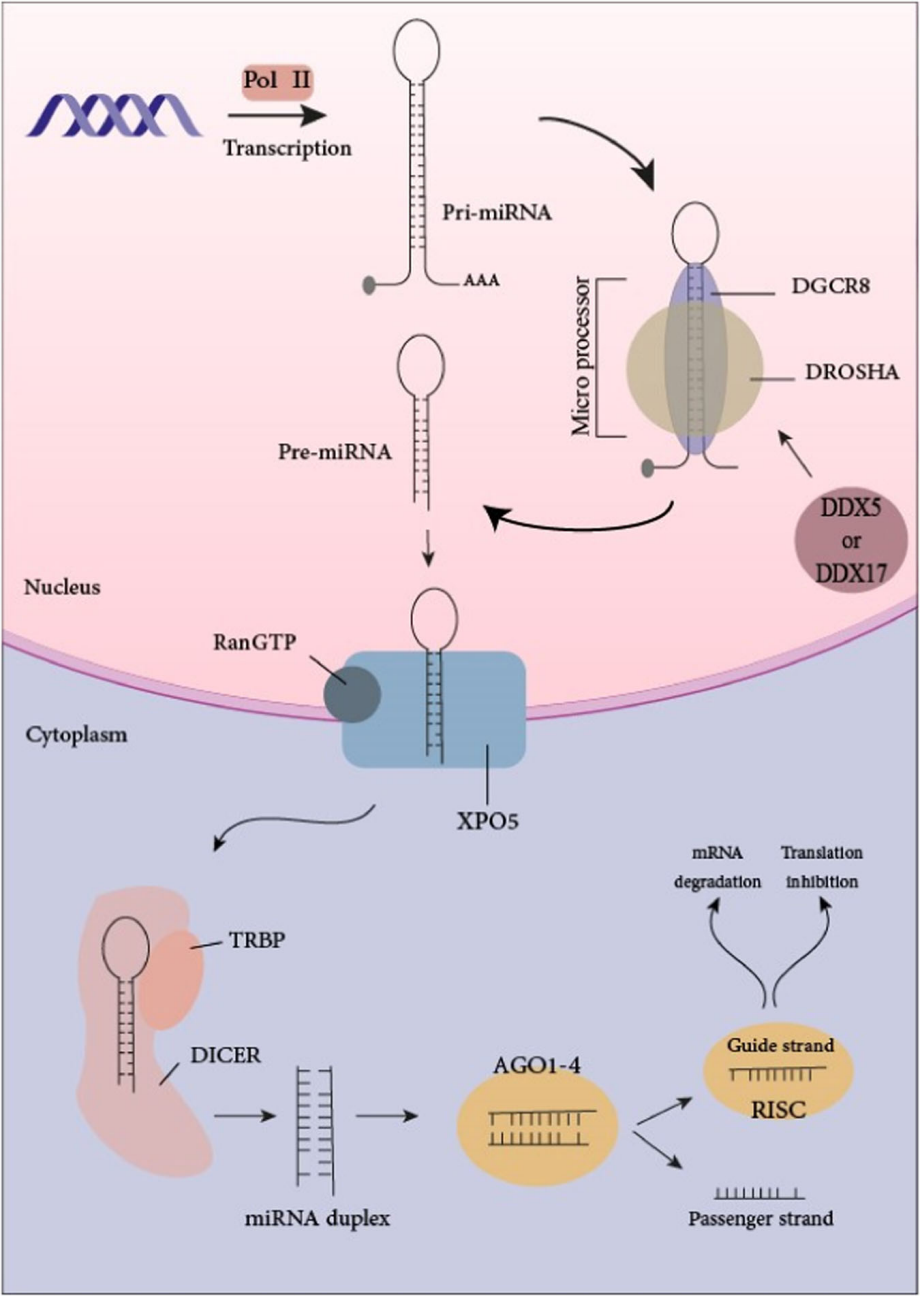
miRNA biogenesis. In the nucleus, RNA Pol II transcripts miRNA genes to produce pri‐miRNAs. Microprocessor complex cleaves pri‐miRNAs which leads to the formation of pre‐miRNAs. Exportin‐5‐Ran‐GTP exports pre‐miRNAs to the cytoplasm where RNase Dicer complex cleaves the pre‐miRNA to produce mature miRNA. RISC effector complex and mature miRNA form miRISC to target mRNAs. [Color figure can be viewed at wileyonlinelibrary.com]

Generally, miRNA biogenesis (Figure 1) and transcription initiate with RNA Pol II from intragenic or intergenic regions. The miRNA primary transcript, termed as pri‐miRNA, is composed of a hairpin structure with 7‐methyl guanylate (m7G) caps and poly A tails in 5′ and 3′ end, respectively.^25^, ^26^ In the next step, the nuclear microprocessor complex, which consists of DGCR8 RNA binding protein, the RNase III enzyme Drosha, and less well‐understood auxiliary factors, including DDX17 and DDX5, processes the primary transcript, leading to the production of ∼70 nucleotide stem–loop precursor known as pre‐miRNA.^27^ Pre‐miRNA is translocated to the cytoplasm by direct interaction with an exportin 5 (XPO5)/RanGTP complex.^28^ In the cytoplasm, it undergoes a final processing stage by the RNase type III endonuclease, Dicer, which results in a mature double strand (ds) miRNA with 20–25 nucleotides long.^29^ Finally, the double‐stranded miRNA is loaded onto an Argonaute (AGO) protein family, which leads to the formation of RNA‐induced silencing complex (RISC) for strand separation. AGO protein selects one strand to become the mature miRNA (guide strand), whereas the other strand is usually degraded (passenger strand).^30^ RISC‐bounded miRNA can silence or degrade hundreds of mRNAs by targeting the 3′‐UTR.^31^, ^32^ The name of the mature miRNA depends on the directionality of the miRNA strand, both 5′ and 3′ strands of miRNA duplex can be loaded onto the AGO protein family.^33^ The selection is based on 5′ nucleotide of each strand and thermodynamic stability.^34^ Most commonly, the strand with lower 5′ uracil or stability is expected to be the guide strand.^35^

## 3 ROLES OF THE miRNAs IN VIRAL INFECTIONS

The impact of miRNAs in an extensive number of biological pathways is well established. To modulate viral infections, miRNAs can target viral genomes or cellular factors. They are classified as antiviral or pro‐viral based on their function in viral infections. In pro‐viral function, viruses use host miRNAs to their advantage to facilitate their own replication and infection. Pro‐viral miRNAs allow viruses to escape from the host immune response through suppressing antiviral factors, including interferon (IFN).^36^, ^37^, ^38^, ^39^ Also, some miRNAs have antiviral effects by enabling infection‐fight mechanisms or activating viral latent state.^40^, ^41^ Furthermore, antiviral host miRNAs can block the viral replication, suppress pro‐viral proteins, or target host mRNAs that encode pro‐viral proteins.^42^, ^43^, ^44^, ^45^ Knowing miRNA's roles in viral infections can facilitate the development of antiviral therapeutic agents.

## 4 LIVER AFFECTING VIRAL INFECTIONS

In the human body, the liver can receive both systemic and portal circulation. Liver possesses one third of the reticuloendothelial system cells and has a crucial role in host defense against invasive microorganisms.^46^ Definition of hepatitis is inflammation of the liver and is most frequently caused by hepatocyte‐tropic viruses including hepatitis A, hepatitis B, hepatitis C, and less frequent hepatitis D and E.^47^ Infection with chronic hepatitis B virus (HBV) and hepatitis C virus (HCV) can lead to chronic liver diseases and hepatocellular carcinoma (HCC).^48^ As blood‐borne pathogens in the global community, we will focus on HBV and HCV infections and their relation with host miRNAs.

### 4.1 Hepatitis B virus

**Figure 2:**
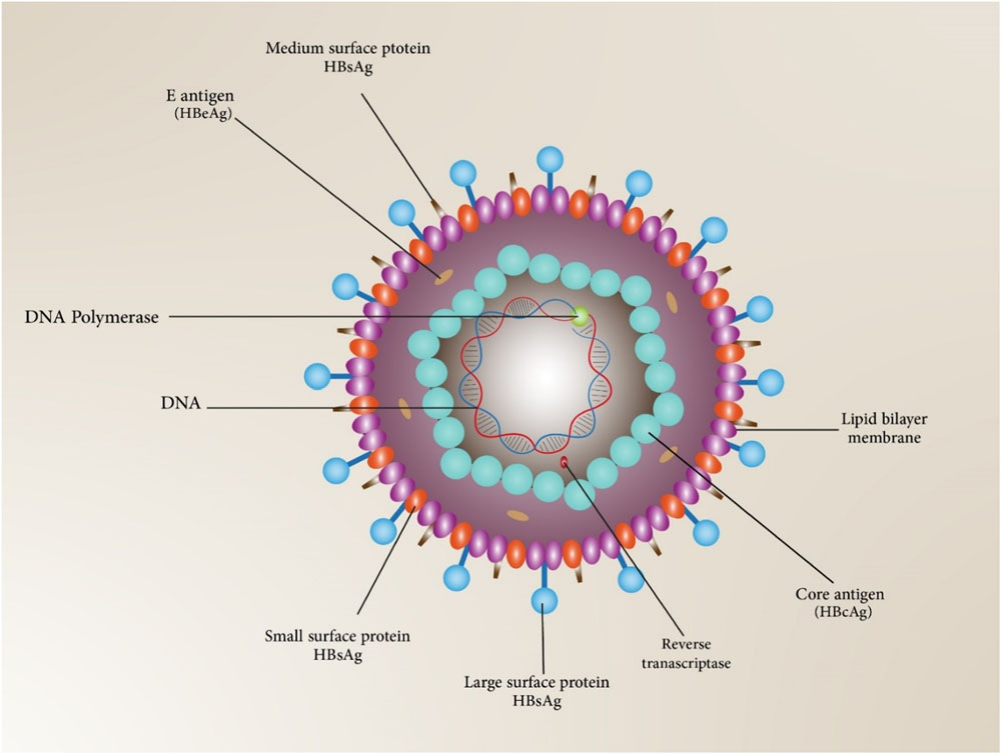
Schematic representation of Hepatitis B virus structure and particles. [Color figure can be viewed at wileyonlinelibrary.com]

Human acute and chronic hepatitis are the consequence of HBV infection.^49^ HBV‐mediated infection is one of the most frequent chronic viral infections around the globe. According to WHO report, HBV has infected 2 billion people. There were 296 million chronic hepatitis B infections in 2019 according to the WHO. Liver cirrhosis and HCC development are great risks of HBV infection.^50^ HBV as a small enveloped virus, is a member of the *Hepadnaviridae* family. The viral genome is comprised of small (3.2 kb), mostly double‐stranded, relaxed‐circular DNA (rcDNA) (Figure 2). HBV has a diameter of 42 nm, and its DNA is protected by a 27 nm internal capsid (core particle).^51^, ^52^ Four overlapping open reading frames (ORFs) are encoded by the viral genome. The viral polymerase, responsible for genome replication, is covalently attached to ds circular DNA. This polymerase is encoded by the largest ORF (polymerase ORF) and has reverse transcription activity that encodes the first strand of the DNA genome from an RNA intermediate. The second largest ORF (PreS1/PreS2/S ORF) has information to encode three viral envelope proteins including large (L‐), medium (M‐), and small (S‐) surface antigen (HBsAg). These envelope proteins and nucleocapsid surround the viral genome. HBV core antigen (HBcAg) is encoded by core ORF. PreC and the core ORF products are cleaved to form HBV E antigens (HBeAg).^53^ HBeAg is categorized as an accessory protein that is not essential for viral replication or infection. However, it plays a crucial role in promoting chronic infection and is directly linked to the modulation of the host's immune responses during chronic infection.^54^ Furthermore, HBeAg facilitates HBV immune escape and suppresses the immune response to HBcAG.^55^

The HBV replication cycle is initiated by the entrance of the virus into host cells. HBV, as a highly hepatotropic virus, recognizes heparan sulfate proteoglycans (HSPG) and sodium‐taurocholate cotransporting polypeptide (NTCP) receptors on the surface of liver cells. The virus makes low‐affinity interaction with HSPG and high‐affinity interaction with NTCP. L protein of HBsAg mediates the binding of the virus to NTCP through its preS1 region.^56^, ^57^ Following the entrance of the HBV into the hepatocytes, the nucleocapsid is released. rcDNA enters into the nucleus and is converted to covalently closed circular DNA (cccDNA) form.^52^ As cccDNA cannot be eliminated or deactivated, it plays a pivotal obstacle role in chronic hepatitis B treatment.^58^ cccDNA, as a microchromosome, utilizes host RNA polymerase for the transcription of viral RNAs, including pregenomic RNA (pgRNA).^52^ The viral polymerase encapsidates the pgRNA. Within the nucleocapsid, the process of reverse transcription occurs. This mechanism utilizes the pgRNA as a template to produce produce single‐stranded (ss) linear DNA, which subsequently forms partially double‐stranded relaxed circular (rc) DNA.^59^ The last steps are viral packaging, maturation, and budding.

#### 4.1.1 Role of miRNAs in HBV infection

Studies on the role of miRNAs in viral replication have shown that miRNAs can promote viral replications or play an inhibitory role. hsa‐miR‐155 is a miRNA that modulates HBV replication in HepG2215 cell lines. hsa‐miR‐155 is downregulated during HBV infection. Furthermore, excessive expression of the hsa‐miR‐155 downregulated SOCS1, repressed mTOR phosphorylation, and reinforced Akt phosphorylation. With ectopic expression, HBV replication in liver cancer cells is reinforced by hsa‐miR‐155 by affecting the SOCS1/Akt/mTOR‐autophagy axis. Transfection of HepG2215 cells by miR‐155‐expressing pCDNA31BIC elevated HBV mRNA, HBeAg, and HBsAg concentration.^60^ Also, hsa‐miR‐155 can mildly inhibit HBV infection by targeting SOCS1 via promoting JAK/STAT signaling in human hepatoma cells, which enhances innate antiviral immunity.^61^

Fu et al. identified XIAP as a direct target of miR‐146a‐5p. They demonstrated that miR‐146a‐5p targets the XIAP‐mediated MDM2/p53 axis, which leads to positive feedback by regulating autophagy‐induced HBV replication. In patients with nonsevere and severe hepatitis, expression levels of serum hsa‐miR‐146a‐5p were different. They found that HBV core protein (HBc) and HBx protein play a role in modulating hsa‐miR‐146a‐5p expression through the nuclear factor‐κB pathway. Furthermore, they suggested that hsa‐miR‐146a‐5p was overexpressed in a large collection of patients with chronic hepatitis B (CHB) and HBV‐expressing hepatocytes. This study indicated that HBV infection plays a role in increased expression of hsa‐miR‐146a‐5p which resulted in promoted HBV replication. These findings can lead to a therapeutic avenue for the prevention and treatment of HBV‐induced CHB.^62^

The role of hsa‐miR‐224 in HBV infection was investigated with hsa‐miR‐224 mimic transfection into Huh7‐1.3 cells. These cells showed a dramatic reduction in SIRT1 expression and LC3‐II protein as well as a significant increase in p62 expression. Accordingly, miR‐224 might inhibit autophagy formation by repressing SIRT1 and autophagic proteins. Furthermore, it was indicated that hsa‐miR‐224 mimic inhibited and suppressed viral infection, HBV antigen secretion, autophagosome formation, HBeAg and HBsAg expression, and viral cccDNA replication and might repress HBV replication. As for hsa‐miR‐224 effect on HBV infection, this microRNA can be considered as a potential factor for the treatment of HBV infection.^63^

hsa‐miR‐1236 and hsa‐miR‐204 have an inhibitory role in HBV infection. These miRNAs were downregulated in HBV‐producing cells. Cloning of the hsa‐miR‐1236 and hsa‐miR‐204 precursors in miRNA expression vectors and cotransfection of the HepG2 cells with these vectors and an HBV *ayw* genomic dimer plasmid showed that both miRNAs could inhibit replication of HBV DNA. HBV can activate host transcription factor STAT3 which leads to suppressing of hsa‐miR‐204 expression. hsa‐miR‐204 can inhibit capsid assembly and HBV pregenomic RNA encapsidation. Also, hsa‐miR‐204 has a tumor suppressor role, and the incidence of HCC may be related to chronic suppression of miR‐204 by HBV. In addition, a novel role was discovered for intronic hsa‐miR‐1236. It was demonstrated that hsa‐miR‐1236 can directly target HBV‐specific RNA which resulted in inhibiting HBV replication.^64^

Chen et al. demonstrated the role of a liver‐specific miRNA, hsa‐miR‐122, in downregulation of HBV gene expression and replication. The target sequence of miR‐122 is located in the 3′‐UTR of the core protein mRNA and coding region of the viral polymerase mRNA. HBV infection and replication reduce hsa‐miR‐122 expression, and increased levels of hsa‐miR‐122 reduce HBV gene expression and replication.^65^

hsa‐miR‐802 was upregulated in the HBV‐associated HCC. Comparison of the HBV‐associated HCC tissues with adjacent noncancerous samples showed a negative relation of the hsa‐miR‐802 with the expression of the SMARCE1. HBV infection of the HepG2.2.15 cells upregulated the miRNA level and downregulated SMARCE1 expression. SMARCE1 has an important function in the regulation of transcription and replication of the virus by attaching to mutant core promoter of HBV. A significant increase in HBV DNA replication was observed following hsa‐miR‐802 ectopic expression and led to increased viral replication through promoting HbsAg and HbeAg expression. Taken together, hsa‐miR‐802 has an important role in HBV pathogenesis and replication by promoting viral DNA replication by regulating the expression of the SMARCE1.^66^

#### 4.1.2 Viral miRNAs

**Figure 3:**
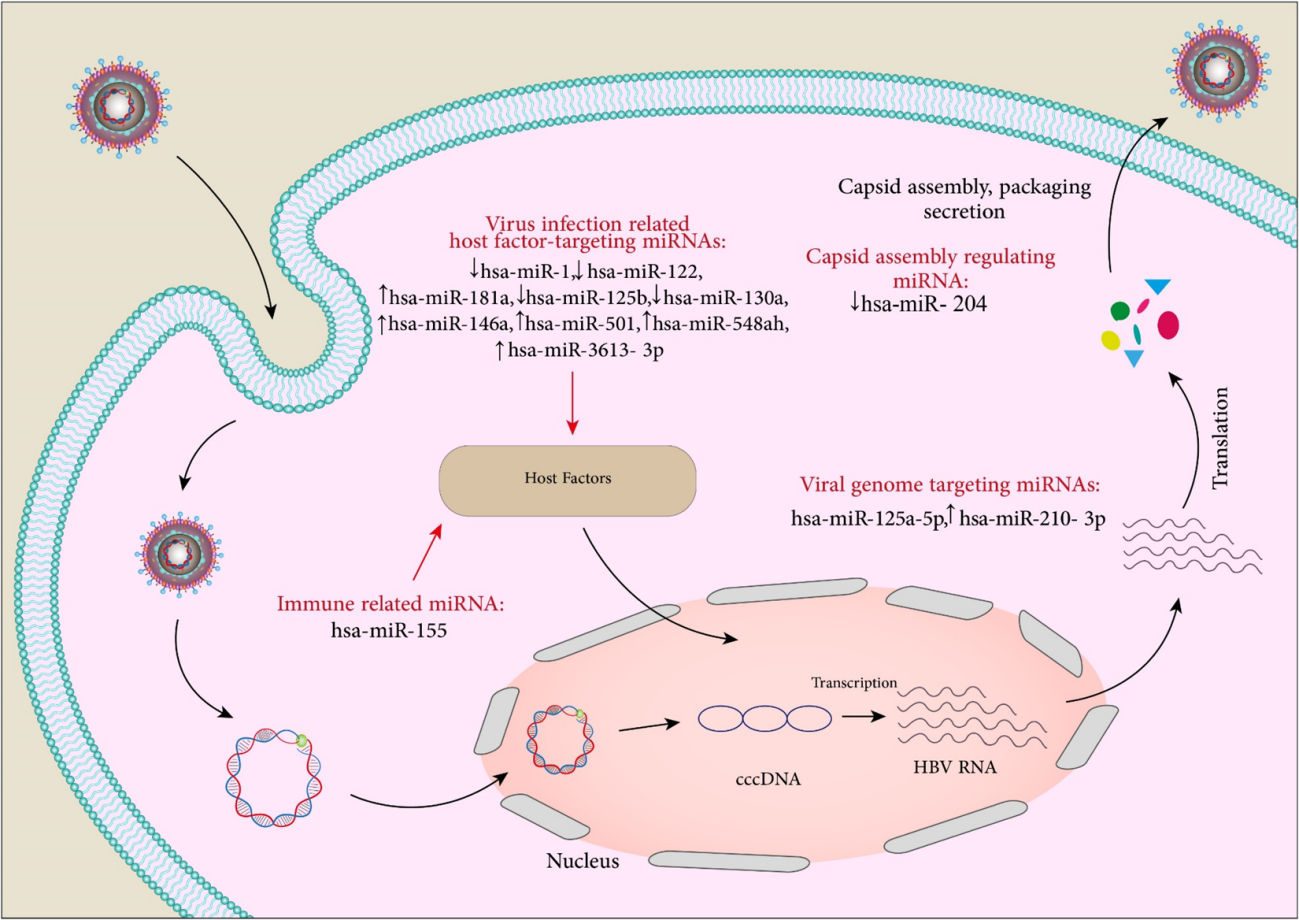
Summary of cellular miRNAs effect on HBV infection. Host cellular miRNAs including hsa‐miR‐1, hsa‐miR‐122, hsa‐miR‐181a, hsa‐miR‐125b, hsa‐miR‐130a, hsa‐miR‐146a, hsa‐miR‐501, hsa‐miR‐548ah, and hsa‐miR‐3613‐3p are involved in HBV pathogenesis through targeting and modulating host factors. Also, hsa‐miR‐125a‐5p and hsa‐miR‐210‐3p can modulate HBV infection by interfering with the translation of viral HBsAg mRNA and upregulating viral HBx expression, respectively. hsa‐miR‐204 can also inhibit capsid assembly. miRNA dysregulation is indicated by an up or down arrow. [Color figure can be viewed at wileyonlinelibrary.com]

As mentioned earlier, some viruses can encode their own miRNAs. HBV‐miR‐3 is one of these miRNAs which is encoded by HBV‐infected cells and HBV‐replicating HCC cells. HBV‐miR‐3 could decrease PTEN expression by binding to its 3ʹ‐UTR. This viral miRNA increased cell proliferation, invasion, and reduced cancer cell apoptosis.^67^ In another study, it was demonstrated that the levels of HBV‐miR‐3 were increased in HBV‐infected HepG2‐NTCP cells. HBV‐miR‐3 downregulated SOCS5 which led to activation of the JAK/STAT signaling pathway. These regulations enhance the anti‐HBV effect induced by IFN which restrains HBV replication.^68^ Table 1 contains additional miRNAs and Figure 3 summarizes the effect of cellular miRNAs in HBV infection.

**Table 1:** miRNAs and their contribution to HBV infection.

miRNA	Expression	Target/human or virus	Effect	References
hsa‐miR‐146a	Upregulated	ZEB2 (Zinc Finger E‐box‐binding homeobox 2)/HumanSTAT1/Human	By targeting and inhibiting the expression of ZEB2, miR‐146a promotes the replication and expression of HBV. Inhibits the production of type I interferon‐induced antiviral factors, inhibits the expression of STAT1.	69, 70
hsa‐miR‐17‐92 cluster	Upregulated	HBV transcripts/Virus	c‐Myc regulates HBV‐induced overexpression of the miR‐17‐92 cluster (hsa‐miR‐17‐5p, hsa‐miR‐20a, and hsa‐miR‐92a‐1). miR‐20a and miR‐92a‐1 directly target HBV. Suppresses HBV infection.	71
hsa‐miR‐3613‐3p	Upregulated	CMPK1/Human decrease expressions of IFN‐α and IFN‐β/Human	Overexpression of the miRNA decreases HBsAg and HBeAg levels while increases the HBV DNA copies, overexpression of the miRNA reduces CMPK1 mRNA and protein levels. Enhances HBV‐induced immune suppression in mice infected with HBV and injected with miR‐3613‐3p.	72
hsa‐miR‐137	Upregulated	PIAS2 (The protein inhibitor of activated STAT 2)/Human	Promotes the expression of HBV genes and viral replication, suppresses PIAS2 mRNA and protein expressions.	73
hsa‐miR‐155	Not reported	SOCS1/Human	Mildly suppresses HBV infection by augmenting IFN signaling pathway and promoting JAK/STAT signaling pathway. Suppresses HBV transcription, and SOCS1 expression. In vitro, miR‐155 inhibits HBV X gene expression to some extent.	61
hsa‐miR‐181a	Upregulated	E2F5/Human	HBV downregulates E2F5 expression and upregulates miR‐181a expression, upregulation of the miRNA downregulates E2F5 expression, induces cell growth, and may have a role in the progression of HCC (an inhibitor of HBV replication).	74
hsa‐miR‐501	Upregulated	HBXIP (HBV replication inhibitor)/Human	Upregulated in hepatocellular carcinoma tissues, and downregulation of the miR inhibits viral replication. Upregulated miRNA induces HBV replication by targeting HBXIP.	75
hsa‐miR‐1231	Upregulated	HBV core mRNA/Virus, HBc/Virus	Overexpression of the miRNA suppresses HBV replication, HBc protein, and HBV core.	76
hsa‐miR‐372/373	Upregulated	NFIB (nuclear factor I/B)/Human	Promotes HBV expression, represses NFIB expression. Increased miRs‐372/373 expression stimulated the production of HBV proteins.	77
hsa‐miR‐331‐3p	Upregulated	ING5/Human	HBV upregulates miRNA, HBx promotes miRNA expression, hsa‐miR‐331‐3p represses the expression of ING5 and promotes the proliferation of HCC cells.	78
hsa‐miR‐99 family	Upregulated	IGF‐1R/PI3K/Akt/mTOR/HumanmTOR/ULK1/Human	Attenuates IGF‐1R/Akt/mTOR pathway signaling, regulates HBV replication at posttranscriptional steps. Promotes autophagy through mTOR/ULK1, enhances HBV replication.	79
hsa‐miR‐21	Upregulated	IL‐12/Human	HCC cells highly express hsa‐miR‐21, upregulation of the miR increases proliferation and decreases IL‐12. Overexpression of HBx inhibits IL‐12. HBx‐induced hsa‐miR‐21 inhibits the apoptosis of the HCC cell.	80
hsa‐miR‐548ah	Upregulated	Histone deacetylase (HDAC) 4/Human	Inhibits HDAC4, promotes the replication and expression of HBV. The expression of the miRNA might be enhanced by HBV core antigen.	81
hsa‐miR‐501	Upregulated	HBXIP/Human	Overexpression induces HBV replication, downregulates HBXIP expression which inhibits replication of HBV.	75
hsa‐miR‐520c‐3p	Upregulated	PTEN (Human)	Interaction of viral HBx with CREB1 enhances the expression of hsa‐miR‐520c‐3p. Increased HCC migration and invasion by HBV promoted EMT via the hsa‐miR‐520c‐3p‐PTEN.	82
hsa‐miR‐192‐3p	Upregulated	ZNF143 (human)	hsa‐miR‐192‐3p positively correlates with the serum levels of HBV DNA and HBsAg, enhances HBV replication and transcription by targeting ZNF143 and inhibiting Akt/mTOR signaling in Huh7 and HepG2.2.15 cells. Upregulates nuclear receptor FXRα expression.	83
hsa‐miR‐192‐5p	Upregulated	‐	miR‑192‑5p upregulated in the feces, peripheral blood, and intestinal mucosal tissue samples of HBsAg‑positive patients, mRNA and protein expression of GLP‐1 decreases with upregulation of hsa‐miR‐192‐5p.	84
hsa‐miR‐210	Upregulated	HBsAg pre‐S1 region/Virus	Suppress HBV infection by targeting sequences of HBsAg pre‐S1 region.	85
hsa‐miR‐210‐3p	Upregulated	HBx/Virus	Increased HBV replication in Huh‐7/NTCP cells, upregulates the HBx expression in HBV‐positive HCC cells.	86
Hsa‐miR‐125a‐5p	Not reported	HBsAg mRNA/Virus	Overexpression of the miRNA suppresses HBV infection by interfering with the translation of HBsAg mRNAs.	87
hsa‐miR‐101	Downregulated	FOXO1/Human	HBV infection downregulates hsa‐miR‐101. Overexpression of the miRNA suppresses HBV replication and expression by targeting FOXO1 in HepG2.2.15 cells. Overexpression of FOXO1 promotes HBV replication and expression in HepG2.2.15.	88
hsa‐miR‐122	Downregulated	cyclin G1/HumanHO‐1/Human	Downregulation of the miRNA enhances HBV replication, HBV infection upregulates cyclin G1 which increases HBsAg, HBeAg, and HBV replication. Overexpression of the miRNA inhibits viral expression by inhibiting the expression of HO‐1.	89, 90
hsa‐miR‐130a	Downregulated	PGC1α, PPARγ/Human	HBV infection increases viral replication by increasing PGC1α and PPARγ as HBV replication stimulators through reducing the miRNA expression, overexpression of the miRNA reduces HBV RNA transcription and DNA replication.	91
hsa‐miR‐200c	Downregulated	NFIA/Human	Downregulated in stable HBV‐producing HepG2.2.15 cell line and pHBV1.3‐tranfected Huh7. Overexpression reduces HBsAg, HBeAg, pgRNA and total RNA levels and suppresses HBV replication. miRNA targets NFIA and represses HBV replication, NFIA overexpression increase viral replication.	92
hsa‐miR‐26b	Downregulated	CHORDC1 (cysteine‐ and histidine‐rich domain containing 1)/Human	Increases HBV enhancer/promoter activities and promotes viral transcription, gene expression, and replication, miRNA downregulation increases CHORDC1 protein levels.	93
hsa‐miR‐325‐3p	Downregulated	AQP5/Human	Downregulated miRNA in HBV‐HCC upregulates AQP5 which promotes cell proliferation and suppresses cell apoptosis in HCC cells and, overexpression of the miRNA induces cell apoptosis and inhibits cell proliferation.	94
hsa‐miR‐98‐5p	Downregulated	NIK (NF‐κB‐inducing kinase)/Human	Overexpression of hsa‐miR‐98‐5p decreases the level of HBV DNA, and inhibits proliferation, migration, and invasion of HBV‐HCC, and decreases protein levels of NIK.	95
hsa‐miR‐125b	Downregulated	SCNN1A gene/Human	Overexpression of the miRNA inhibits secretion of HBsAg and HBeAg and HBV expression and inhibits HBV expression by inhibiting the mRNA and protein levels of SCNN1A.	96
hsa‐miR‐212‐3p	Downregulated	NFIA (Human)	Overexpression of the hsa‐miR‐212‐3p inhibits HBV replication and expression by suppressing NFIA.	97
hsa‐miR‐340‐5p	Downregulated	ATF7/Human	Downregulation increases cell proliferation and inhibits apoptosis by enhancing ATF7 expression in in HBV‐associated HCC.	98
hsa‐miR‐1271	Downregulated	CCNA1/Human	Downregulated in HBV‐associated HCC negatively regulates CCNA1. Upregulation of the miRNA activates MPK signaling pathway and inhibits HBV‐DNA replication, invasion, migration, and proliferation of the HBV‐associated HCC cell.	99
hsa‐miR‐1271‐5p	Downregulated	AQP5/Human	miR‑1271‑5p is downregulated in HBV‑related liver cancer cells and tissues, causing AQP5 upregulation as direct target of the miRNA. Overexpression of miR‑1271‑5p inhibited the levels of viral HBeAg, HBsAg, and HBV DNA.	100
hsa‐miR‐1	Downregulated	HDAC4, MET, E2F5/Human	Overexpression enhances HBV replication by regulating the expression of host genes and augmenting FXRA expression, overexpression of the miRNA reduces MET expression and HDAC4 protein. Overexpression inhibits cell proliferation by targeting E2F5.	101, 102, 103

### 4.2 Hepatitis C virus

HCV as a member of the *Flaviviridae* family is classified in *Hepacivirus* genus. It is a hepatotropic blood‐borne virus, which can lead to acute or chronic hepatitis in humans. HCV infection is a global concern and according to WHO report, annually 3–4 million people are infected and approximately 71 million people are struggling with HCV infection.^104^ During the acute infection phase, the virus is spontaneously cleared in nearly 30% of infected patients; however, the remaining 70% with chronic infection develop serious liver disease, including cirrhosis, steatosis, liver fibrosis, and HCC.^105^

**Figure 4:**
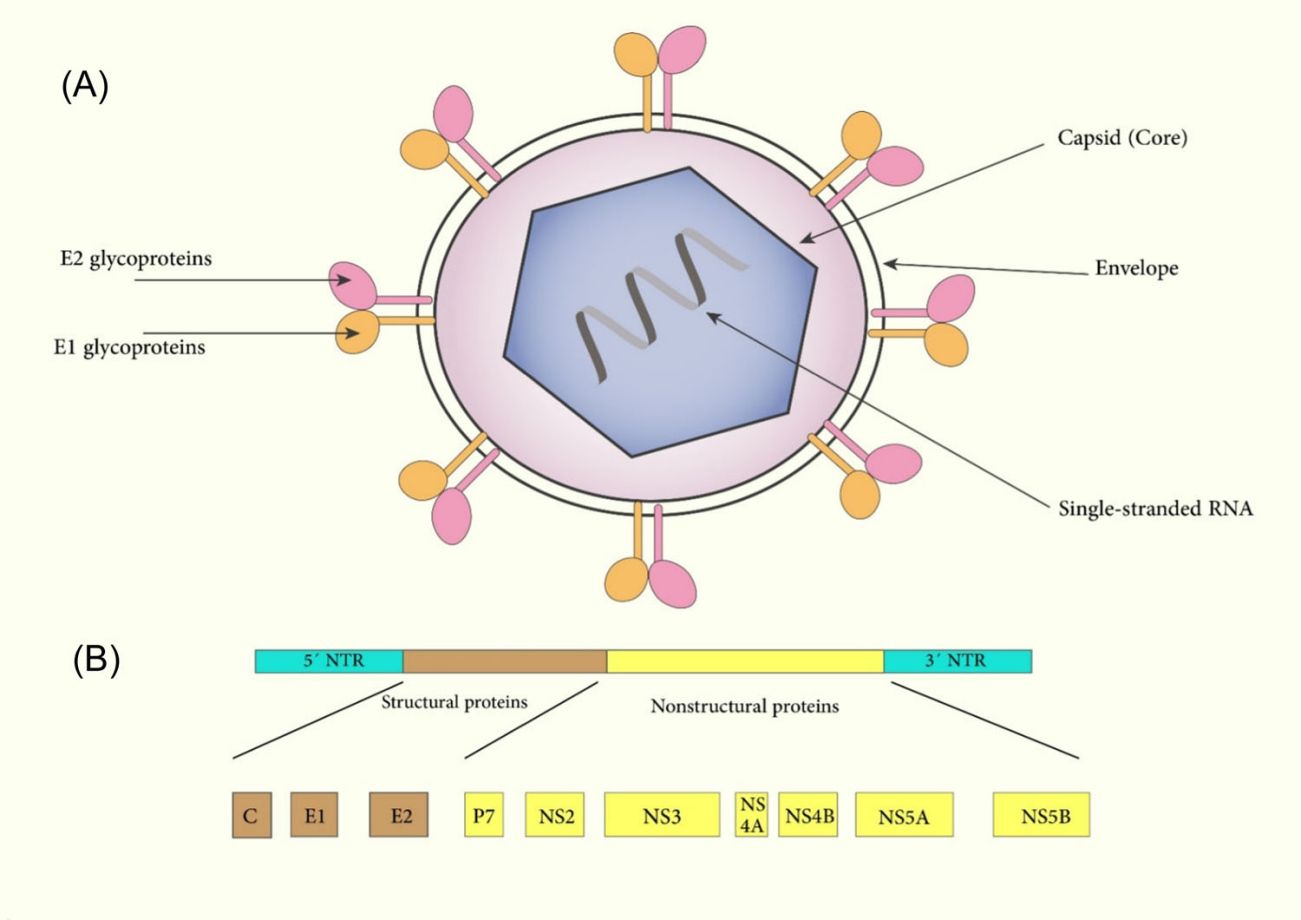
(A) Schematic representation of HCV structure and particles. (B) HVC genome structure. [Color figure can be viewed at wileyonlinelibrary.com]

HCV is an enveloped, icosahedral virus with a size of 55–66 nm.^106^ The viral genome is a positive‐sense 9.6 kb ssRNA that contains a single ORF flanked by conserved 5′‐ and 3′‐UTR, which are vital in HCV replication and translation. Internal ribosome entry site (IRES) is used for ORF translation. The ORF encodes a single polyprotein precursor with almost 3000 amino acids. Viral and cellular proteases cleavage this polyprotein to important HCV replication cycle proteins. Viral cleavage results in seven nonstructural proteins including NS5B (RdRp), NS5A, NS4B, NS4A, NS3 (NTPase/RNA helicase activities and complex harboring protease), NS2 (protease), and p7(viroporin). Cellular protease cleavage leads to three structural viral proteins, including core protein and the envelope glycoproteins E1 and E2.^107^ E1 and E2 heterodimers are enclosed in the lipid bilayer surrounding a nucleocapsid. The nucleocapsid is made up of the ssRNA genome (Figure 4).^108^

Due to the lack of appropriate in vivo infection models and robust cell culture, the HCV replication cycle is not well understood. Furthermore, the involvement of complex and extensive network of surface receptors in viral entry inhibits complete understanding the mechanism of the HCV replication cycle. In the entrance of HCV into hepatocytes, various cellular receptors are involved. E1 and E2 play a role as complexes of disulfide‐bound heterodimers and have a main role in the viral entrance and E2 is responsible for receptor binding. These envelope proteins interact with SR‐BI and CD81 receptors.^109^ Other human receptors including CLDN1^110^ and OCLN^111^ are also involved in viral entry. Additionally, EphA2 and EGFR were found to function as co‐factors for HCV entry; in other words, EGFR facilitates HCV entry into the target cell quickly and effectively and it regulates trafficking of cell surface CLDN1, CD81, or both. Finally, the formation of CD81‐CLDN1 coreceptor complexes, which are crucial for HCV entry, is regulated by EGFR and EphA2.^112^ Following HCV binding, the endocytic pathway and membrane fusion are responsible for internalizing HCV particles.^113^ In the liver with the same overlapping host entry factors, virus can transmit through direct cell‐to‐cell transmission, so, it creates conditions to evade neutralizing antibodies and immune response.^114^ The entrance stage continues with the fusion of host membrane and HCV through clathrin‐dependent endocytosis and low pH‐dependent fusion with endosomes, resulting in the viral uncoating, release of the HCV genome in the cytoplasm and the initiation of primary translation.^113^ The translation of IRES region in 5′‐UTR of the HCV through the mechanism of endoplasmic reticulum (ER)‐associated translation results in the formation of HCV single polypeptide. Cellular (signal peptide peptidase and signalase) and viral (NS3‐NS4A and NS2‐NS3) proteases co‐ and post‐translationally cleavage this polyprotein to form structural and nonstructural (NS) proteins.^108^, ^115^ It is believed that NS4B and NS5A induce biogenesis of ER‐derived membrane spherules called “membranous web” which is the place for RNA replication and contains viral replication factors.^116^, ^117^ The viral positive RNA genome is utilized as a template for negative‐strand synthesis. The key enzyme of RNA synthesis in the HCV replicase complex is NS5B, which is an RNA‐dependent RNA polymerase and has a classical right‐hand structure.^118^

The assembly site is near replication complexes and it is related to ER‐derived membranes next to lipid droplets where core proteins can aggregate.^119^, ^120^ Domain I and II of the NS5A phosphoprotein are essential in RNA replication, while domain III is involved in virus assembly. Also, NS5A has a role in RNA genome delivery to the core proteins.^121^, ^122^ Viral NS2 and p7 also have an important function in viral assembly by gathering all virus proteins, including E1E2 envelope glycoproteins, NS5A, and NS3 to the assembly sites.^123^, ^124^, ^125^ We described the HCV replication cycle briefly; however, as mentioned earlier, HCV replication cycle is partially understood.

#### 4.2.1 Role of miRNAs in HCV infection

hsa‐miR‐122 as a highly abundant liver‐specific miRNA plays a pro‐viral role by attaching to the 5′‐UTR of the viral genome and facilitating the replication of the HCV RNA. Also, in vitro inhibition of hsa‐miR‐122 can lead to marked loss of HCV RNAs.^126^ Another study by Henke et al. indicated that 5′‐noncoding region of HCV and hsa‐miR‐122 attachment is important in HCV RNA maintenance and can also enhance the binding of the ribosome to the HCV RNA, which stimulates HCV translation.^127^ hsa‐miR‐122‐associated Argonaute proteins binding to the 5′ end of the HCV genome led to slow decay of the HCV genomic RNA. This attachment also protected the viral genome from 5′ exonuclease activity of exoribonuclease 1 (Xrn1).^128^, ^129^ In nonpermissive cell line, sufficient HCV RNA replication can be reached through exogenous expression of hsa‐miR‐122.^130^

hsa‐miR‐21 upregulation showed a pro‐viral effect in HCV infection. Two HCV proteins, including NS5A and NS3/4A played a role in HCV‐induced hsa‐miR‐21 expression by activating protein‐1 (AP‐1). NS3/4A and NS5A regulated this miRNA expression through c‐Fos and c‐Jun, respectively. Also, these viral factors can trigger PKCα‐ERK and PKCε‐JNK, respectively. Furthermore, regulation of hsa‐miR‐21 expression induced by HCV infection resulted in suppression of type I IFN production through targeting IL‐1 receptor associated kinase 1 (IRAK1) and myeloid differentiation factor 88 (MyD88) as important TLR signaling pathway factors. These processes led to promoting HCV replication and facilitating evasion to the host immune system. Also, this miRNA can counteract the antiviral activity of IFN‐α and facilitate HCV replication.^131^

Patra et al. indicated that HCV infection resulted in the downregulation of hsa‐miR‐181c in hepatocytes through modulating of C/EBP‐β. hsa‐miR‐181c directly targetes Ataxia‐telangiectasia mutated (ATM) protein kinase and can interact with the 3′‐UTR of ATM. In HCV‐infected hepatocytes, ATM expression is higher due to the inhibition of hsa‐miR‐181c. Moreover, HCV infection enhances expression of the ATM at the transcriptional level. This protein kinase functions as a DNA damage response element. Furthermore, transfection of hsa‐miR‐181c mimic and overexpression of this miRNA led to the suppression of ATM expression. This study suggested that suppressing of hsa‐miR‐181c can combat DNA damage by HCV‐induced ATM expression.^132^

Even though many studies have been conducted, some miRNAs function is controversial on HCV replication. hsa‐miR‐130a is one of these miRNAs. HCV infection upregulates hsa‐miR‐130a expression which results in inhibition of IFN‐induced transmembrane protein 1 (IFITM1).^133^ hsa‐miR‐130a knockdown reduces HCV replication by enhancing of IFITM1 expression.^134^ However, another study indicated that HCV replication is inhibited by upregulation of hsa‐miR‐130a through restoring the expression of IFN‐α/β, ISG15, USP18, and MxA in RIG‐I and TLR3 deficient hepatocytes. These downregulated expression of hsa‐miR‐122 promotes HCV production.^106^

**Figure 5:**
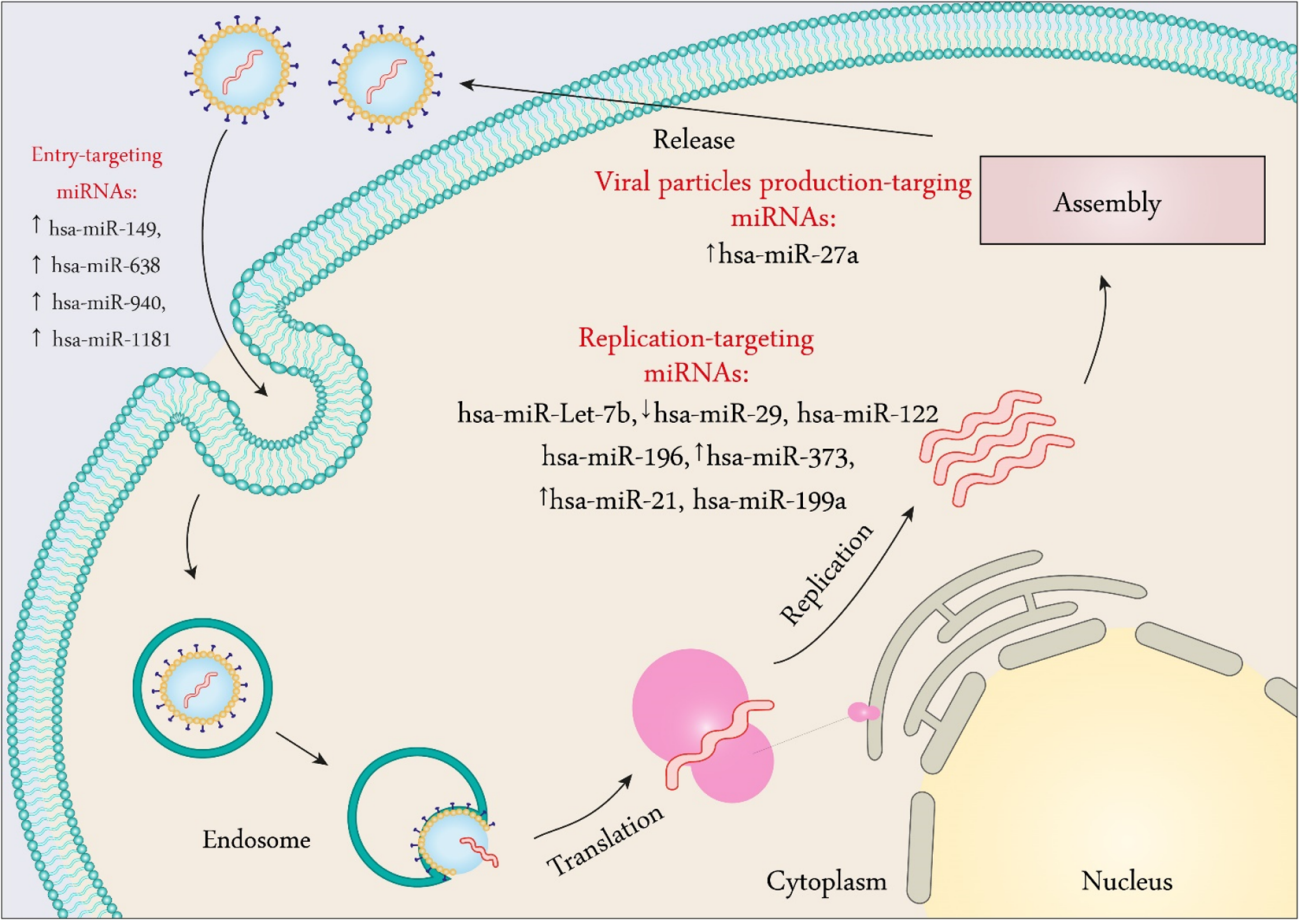
Summary of cellular miRNAs involved in HCV infection. Host miRNAs including hsa‐miR‐149, hsa‐miR‐638, hsa‐miR‐940, and hsa‐miR‐1181 slightly decrease viral entry. hsa‐miR‐27a regulates the expression of genes essential for the generation of infectious viral particles. hsa‐miR‐Let‐7b, hsa‐miR‐29, hsa‐miR‐122, hsa‐miR‐196, hsa‐miR‐373, hsa‐miR‐21, and hsa‐miR199a regulate viral replication. miRNA dysregulation is indicated by an up or down arrow. [Color figure can be viewed at wileyonlinelibrary.com]

Further information about the function of more miRNAs in HCV pathogenesis is highlighted in Table 2 and summarized in Figure 5.

**Table 2:** miRNAs and their contribution to HCV infection.

miRNA	Expression	Target/human or virus	Effect	References
hsa‐miR‐29	Downregulated	COL1A1, COL3A1/Human	Overexpression of the miR‐29 decreases collagen synthesis, suppresses fibrosis progression, reduces HCV RNA abundance, and suppresses COL1A1 and COL3A1.	135, 136
hsa‐miR‐30 cluster	Downregulated	‐	Expression of the hsa‐miR‐565, hsa‐miR‐324‐5p, hsa‐miR‐301, hsa‐miR‐192, hsa‐miR‐130a, hsa‐miR‐30c, and hsa‐miR‐30b downregulated in HCV‐infected Huh7.5 cells, interferon‐α treatment upregulated listed miRNAs. HCV replication was significantly enhanced by inhibition of miR‐30c expression, inhibition of miR‐130a decreased HCV RNA levels.	137
hsa‐miR‐181c	Downregulated	E1 and NS5A regions of HCV genome/VirusHOXA1/Human	HVC NS5A protein inhibits promotor activity of miR‐181c and downregulates the expression of the miRNA, overexpression of hsa‐miR‐181c reduces the viral replication by directly binding to NS5A and E1, inhibits HOXA1.	138
hsa‐miR‐196	Downregulated	Bach1/HumanNS5A region in HCV genome/Virus	Overexpression targets and suppress Bach1 mRNA and downregulates NS5A mRNA expression, hsa‐miR‐196 downregulates Bach1 which downregulates HCV replication.	139, 140
hsa‐miR‐491	Downregulated	PI3K/Akt pathway/Human	HCV infection downregulates miR‐491 expression, forced expression of the miRNA enhances HCV abundance, suppresses PI3K/Akt pathway.	141
hsa‐miR‐942	Downregulated	ISG12a/Human	HCV infection decreases expression of the miRNA in HLCZ01 cells, overexpression of the miRNA suppressed HCV infection induced apoptosis by decreasing ISG12a expression.	142
hsa‐miR‐107/449a	Downregulated	IL6, JAK1/Human	HCV infection downregulates miRNAs, downregulation of the miRNAs activates IL6 which regulate CCL2 expression, causes HCV‐mediated fibrosis and inflammatory responses.	143
hsa‐Let‐7a‐1	Downregulated	‐	hsa‐Let‐7a‐1 downregulates in the serum of HCV patients and leads to the development of HCC.	144
hsa‐miR‐16	Upregulated	HGF, Smad7/Human	HCV infection upregulates miRNA expression, downregulates Smad7 and HGF, have a role in development of liver fibrosis.	145
hsa‐miR‐21	Upregulated	IRAK1, MyD88, type I IFNSMAD7/Human	Promotes HCV replication by suppressing type I IFN, decreases MyD88 and IRAK1 expression, the NS5A and NS3/4A proteins of HCV induce the expression of miRNA through the AP‐1, the NS5A protein primarily regulates miRNA via c‐Jun, while the NS3/4A protein primarily regulates through c‐Fos. Inhibits SMAD7 and enhances TGF‐β‐mediated fibrosis.	131, 146
hsa‐miR‐21‐5p	Upregulated	PTEN/Human	HCV‐3a core transduction or HCV‐Jc1 infection increases the expression of the miRNA, HCV‐3a core induces PTEN downregulation, promotes HCV‐3a core‐induced steatosis and HCV replication.	147
hsa‐miR‐27a	Upregulated	RXRα, and lipid metabolism‐related genes/Human	Reduces lipid storage and enhances IFN signaling, suppress the expression of genes involved in lipid metabolism essential for the generation of infectious viral particles, represses HCV infection and replication.	148
hsa‐miR‐27b	Upregulated	Aquaporin 11 (AQP11)/Human	HCVcc infection upregulates miRNA, hsa‐miR‐27b indirectly suppresses AQP11 expression, HCVcc infection suppresses the expression of AQP11 which results in suppression of HCV genome replication and reduce HCV genome levels.	149
hsa‐miR‐93‐5p	Upregulated	IFNAR1/Human	HCV‐1b core protein increases the expression of the miRNA, miRNA decreases IFNAR1 protein and mRNA levels, HCV‐1b infection increases miRNA levels and inhibits the IFN signaling pathway.	150
hsa‐miR‐125a	Upregulated	MAVS, TRAF6/Human	HCV infection upregulates expression of the miRNA, miR‐125a promotes HCV infection and enables the virus to evade innate antiviral immunity by inhibiting the expression of MAVS and TRAF6.	151
hsa‐miR‐373	Upregulated	JAK1, IRF9/Human	Directly targets JAK1 and IRF9, inhibits IFN response. Enhances HCV RNA replication.	152
hsa‐miR‐758	Upregulated	TLR3, TLR7/Human	Inhibits the expression of TLR3 and TLR7, reduces IFN‐α and IFN‐β response and signaling.	153
hsa‐miR‐Let‐7b	Downregulated	5′UTR of the HCV genome, NS5B/Virus	Binds to the NS5B coding sequences and inhibits viral replication.	154
hsa‐miRLet‐7b	Not reported	‐	In the Ago2‐containing miRNP complex, miRNA directly interacts with HCV RNA. Let‐7b decreases viral NS5A, reduces HCV replication and raises cell apoptosis rate.	155
hsa‐miR‐Let‐7c	Not reported	Bach1/Human	Overexpression reduces HCV NS5B and core protein levels, reduces HCV RNA levels, stimulate HO‐1 expression and reduces HCV replication by targeting Bach1.	156
hsa‐miR‐199a	Upregulated	‐	Inhibits HCV replication.	157
hsa‐miR‐149/638/1181/940	Upregulated	‐	Slightly decrease HCV entry.	158
hsa‐miR‐146a‐5p	Upregulated	‐	Increases viral infection by promoting last steps of the HCV replication, regulates by NF‐κB signaling.	159

## 5 RESPIRATORY AFFECTING VIRAL INFECTIONS

Virus‐caused respiratory infections are one of the most pivotal global health problems that cause mortality and morbidity of people of all ages over the world. Acute respiratory illnesses caused by viral infections can be limited to the upper airways, or they can also involve the lower respiratory tract. Chronic respiratory diseases, including bronchiolitis, chronic obstructive pulmonary disease, asthma, and pneumonia can be caused by viral infections, which lead to enormous economic burdens.^160^, ^161^ Influenza virus (IV) and coronavirus (CoV) are among the most common respiratory viruses and affect an extensive population. These viruses are transmitted mainly through the suspension droplets or fomites.^162^ miRNAs can affect different mechanisms of these viral infections. In this section, we will review the effect of miRNAs on the infection processes of influenza A virus (IAV) and CoV.

### 5.1 Influenza A virus

Influenza viruses belong to the *Orthomyxoviridae* family, which has four different genera: *influenza A*, *B*, *C*, and *D*. Different genera are categorized based on antigenic differences between matrix 1 (M1) protein and nucleoprotein (NP).^163^ IAV can infect humans, birds, and other animals, while *influenza B* and *C* can only infect humans. However, type *A* is the primary agent of annually recurring epidemic disease and global pandemics.^164^, ^165^ IAV can be formed in a ~100 nm sphere or filamentous shape with a length of 20 µm, however, filamentous form is mostly lost.^166^ In cases of acute infection, patients commonly exhibit symptoms such as fatigue, headache, fever, dry cough, sore throat, and inflammation of the upper respiratory tract. Additionally, IAV infection can give rise to complications including ear infections, sinus infections, bronchitis, and pneumonia, which have the potential to cause organ failure and fatality.^167^ Primary influenza viral pneumonia initiates without complications in the upper respiratory system like the common flu; however, as the disease progresses, the symptoms of the disease related to the lower respiratory system such as cough, shortness of breath, and hypoxemia promote. Moreover, following a typical influenza infection, secondary bacterial pneumonia develops.^168^

**Figure 6:**
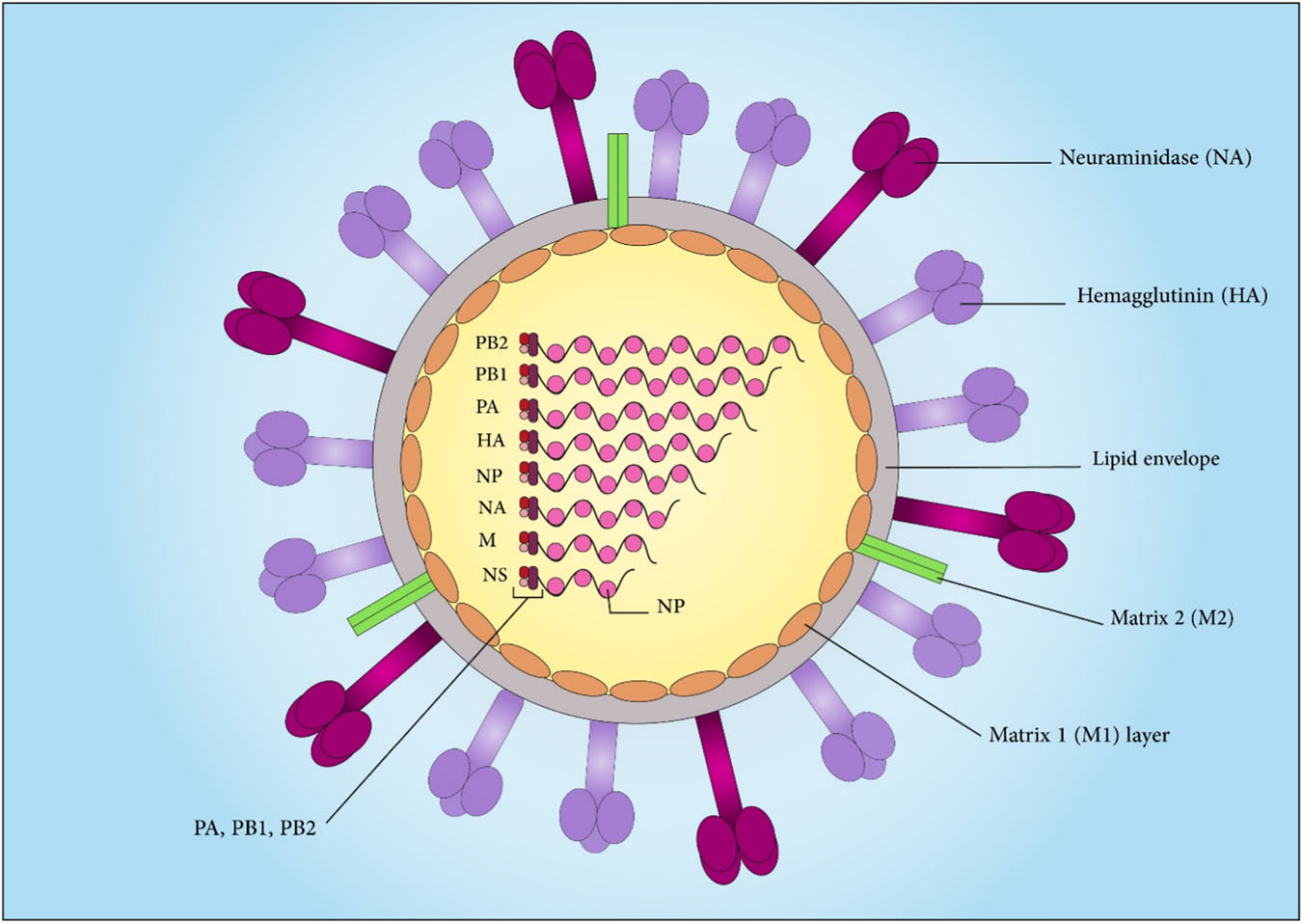
Schematic representation of Influenza A virus structure and particles. A single segment is attached to heterotrimeric viral RNA‐dependent RNA polymerase (PB1, PB2, and PA) and wrapped by multiple nucleoprotein (NP) copies. [Color figure can be viewed at wileyonlinelibrary.com]

IAV genome is a single‐stranded negative‐sense RNA with a total length of 13 kb, which is composed of eight segments. Through the reassortment mechanism (antigenic drift and antigenic shift), these segments can be exchanged. The primary structure of each segment consists of a central negative‐sense protein‐coding sequence (CDS), which is surrounded by 5′‐ and 3′‐terminal UTRs and segments encode one or two proteins.^169^ Proteins encoded by these segments are: PB2 (basic polymerase 2, regulates host‐cell RNA recognition), PB1 (basic polymerase 1, catalyzes nucleotide addition), PA (polymerase acidic protein, encodes three proteins and transcriptase protease activity), HA (hemagglutinin, divides into HA1 and HA2, in virus entry binds to sialic‐acid receptors), NA (neuraminidase, functions in new virions budding), NP (nucleoprotein, binds to the viral RNA), NEP (nuclear export protein also known as NS2), M1 and M2 (matrix proteins, have overlapping regions, M1 encodes component of the viral capsid and M2 functions as ion channel), and NS1 (Non‐structural protein, has a role in translation, splicing, and transport of cellular RNA) (Figure 6).^170^, ^171^ Each viral RNA (vRNA) segment forms a viral ribonucleoprotein complex (vRNP) together with nucleoprotein and viral polymerases (including PB2, PB1, and PA) which is a substantial unit for transcription and replication of the viral genome.^172^, ^173^ IAVs are divided into different subtypes based on their surface HA and NA glycoprotein. There are 18 HA and 11 NA subtypes.^174^ Among them, H1N1 and H3N2 are the main IAV subtypes that regularly infect humans.^175^ The envelope of IAV, supported by M1 protein, is a host‐derived lipid membrane that is decorated by HA, NA, and M2 proteins.^176^, ^177^ Besides, several studies showed the relation of M1 protein with morphologic changes of IAV.^178^, ^179^

IAVs replication cycle is initiated by the binding of HA, one of the important viral ligands, to sialic acid receptors on the cell surface. This attachment results in the entrance of the virus into the cell through an endocytosis‐dependent pathway. The low pH leads to structural changes in the HA protein, which causes the exposure of a fusion peptide. This peptide enables the fusing of the viral envelope with endosomal membrane and the release of vRNPs into the cytoplasm. In addition, hydrogen ions are pumped into the virion through the M2 channel and internal acidification disrupts the interaction between internal proteins, allowing viral RNPs to be released.^180^ Conformational change of M1 dissociates M1 and leads to the apparition of nuclear localization sequences (NLS) on the viral NP, as a part of vRNPs. It mediated the translocation of vRNPs to the nucleus.^181^ Moreover, the negative‐sense RNA genome must be converted into a positive sense to be able to function as a template. IAV polymerase, RdRp, with three PA, PB1, and PB2 subunits starts the synthesis of positive strand. To form a RNP, viral polymerase and NP co‐transcriptionally encapsidate newly‐synthesized vRNA. The PA and PB2 subunits of viral RNA polymerase facilitate initiation of transcription by producing capped primers through binding and cleaving host mRNAs.^182^ Viral proteins regulate the final steps of replication and vRNP assembly. Viral transcribed mRNAs are transported from the nucleus to the cytoplasm for translation. Viral surface proteins such as HA, M2, and NA are processed in the ER and glycosylated in the Golgi apparatus and transported to the cell membrane.^180^ Packaging of viral segments into virions can be done through two mechanisms of the specific and the random incorporation model.^183^ A virion must have all eight segments to be a completely infectious particle.^180^ Finally, in the newly synthesized viral particles, HA and NA proteins, which contain terminal sialic acids, lead to the attachment of the virus to the inner surface of the cell membrane. Subsequently, the NA cleaves the sialic acid residues from both newly synthesized HA and NA and cellular receptors and results in the release of the virus from the host cell.^177^ Interferon pathways and innate immunity can be antagonized by NS1 protein. NS1‐mediated inhibition of the antiviral system against the host can take place in the following ways: competitive binding of NS1 to dsRNA and interacting with RIG‐I receptors, which can directly inhibit the production of IFN, NS1 can also interact with ISGs and also can inhibit expression of mRNAs.^184^

#### 5.1.1 Role of miRNAs in IAV infection

In IAV infection, miRNAs can bind to the RNA virus genome and modulate translation, replication, and viral pathogenesis. They also can regulate host‐related processes, which can be beneficial to virus survival. For the first time in 2010, Song et al. demonstrated the modulation of influenza viral replication by regulation of cellular miRNAs. In this study, they used infected MDCK cells and utilized 3′‐UTR reporter analysis and virus proliferation assay. They found that replication of the H1N1 is inhibited by hsa‐miR‐654, hsa‐miR‐491, and hsa‐miR‐323 through binding to the viral RNA polymerase PB1 gene and its downregulation. The results of this study demonstrated that host miRNAs have a role in the regulation of flu virus replication.^185^

Downregulation of hsa‐miR‐30 in H5N1, H1N1, and also H9N2‐infected A549 cells leads to IAV proliferation. Expression of hsa‐miR‐30 through mimic transfection inhibited IAV proliferation. Its expression also reduced SOCS1 and SOCS3 expression. SOCS1 and SOCS3 have a role in the inhibition of IFN/JAK/STAT signaling pathway. Also, IFITM3 is important in cellular defense against IAV. hsa‐miR‐30 also can inhibit the expression of NEDD4 by targeting 3′‐UTRs of NEDD4 gene. NEDD4 as an E3 ubiquitin ligase promotes IAV replication by negative regulation of this protein. In summary, IAV infection downregulated the expression of hsa‐miR‐30 family members which promoted viral replication through modulating NEDD4, SOCS1, and SOCS3.^186^

H1N1 influenza virus inhibited the expression of hsa‐miR‐93. IAV infection downregulates miRNA through RIG‐1/JNK signaling. In addition, reduced expression of miRNA activated IFN/JAK/STAT pathway and led to the promoting of antiviral innate response. Since JAK 1 is a target of hsa‐miR‐93, overexpression of hsa‐miR‐93 suppressed JAK1 expression, which resulted in increased viral replication by suppressing of IFN‐JAK‐STAT pathway. IAV infection increase JAK1 expression through downregulation of hsa‐miR‐93. Taken together, hsa‐miR‐93 facilitates viral infection. However, H1N1 infection induces the downregulation of hsa‐miR‐93 which leads to upregulation of JNK, JAK1, and antiviral innate response and suppresses IAV replication.^187^

A study by Zhang et al. showed promoted IAV replication through in vitro H3N2 and H1N1 infection of A549 cells which resulted in upregulation of hsa‐miR‐146a in infected cells. They showed that upregulation of hsa‐miR‐146a can decrease IFN‐stimulated gene (ISG) expression and IFN‐β production. In addition, hsa‐miR‐146a attenuated type I IFN responses which exerted pro‐viral effect of this miRNA. Furthermore, this miRNA can target *TRAF6* gene and inhibit its expression. TRAF6 has a role in the production of type I IFN. The knockdown of this gene promotes IAV replication. Inhibition of hsa‐miR‐146a suppressed IAV replication and alleviated IAV‐induced lung injury in mice. This study demonstrated that overexpression of hsa‐miR‐146a has a pro‐viral effect and antagomir therapy might be effective during IAV infection.^188^

hsa‐miR‐203 was identified as an inhibitor of viral replication and was upregulated in IAV‐infected A549 cells. Two different mechanisms are involved in the upregulation of hsa‐miR‐203 in IAV infection. In the first mechanism, type I IFN induces expression of this miRNA. It is confirmed that IFNs are activated during IAV infections as players of host innate immune responses. In the second mechanism, H5N1 infection leads to downregulation of DNA methyltransferase 1 expression. Therefore, DNA demethylation in promoter of hsa‐miR‐203 mediates upregulation of this miRNA. In addition, ectopic expression of hsa‐miR‐203 targeted downregulator of transcription 1 (DR1) which inhibited IAV infection by increasing antiviral responses. In summary, upregulation of hsa‐miR‐203 induced by IAV infection inhibits viral replication through targeting DR1.^189^

Ingle et al. demonstrated that hsa‐miR‐485 regulates host and IAV transcripts through a dual role and restricts viral replication. They found that influenza virus H5N1 infection induced upregulation of hsa‐miR‐485. At lower doses of the influenza virus, hsa‐miR‐485 targeted 3′‐UTR of RIG‐1 which suppresses antiviral response. Retinoic acid–inducible gene I (RIG‐I) is known as a cytosolic sensor which induces type I IFN production by sensing viral RNA in the cytosol. In increased amount of IAV, hsa‐miR‐485 switched the target to viral PB1 by binding to its gene. PB1 gene encodes viral RNA polymerase. This binding resulted in inhibition of IAV replication and enhanced antiviral response.^190^

hsa‐miR‐584‐5p and hsa‐miR‐1249 are two downregulated miRNAs in IAV infection. These miRNAs downregulate the expression of the viral protein PB2 which leads to inhibition of H1N1 and H5N1 replication. IAV‐induced downregulation of these miRNAs results in enhanced PB2 expression and promoted IAV replication in A549 cells.^191^ Also, IAV infection upregulates hsa‐miR‐101 expression. This upregulation decreases viral NP expression, leading to a reduction in IAV replication and titer.^192^

#### 5.1.2 Viral miRNAs

**Figure 7:**
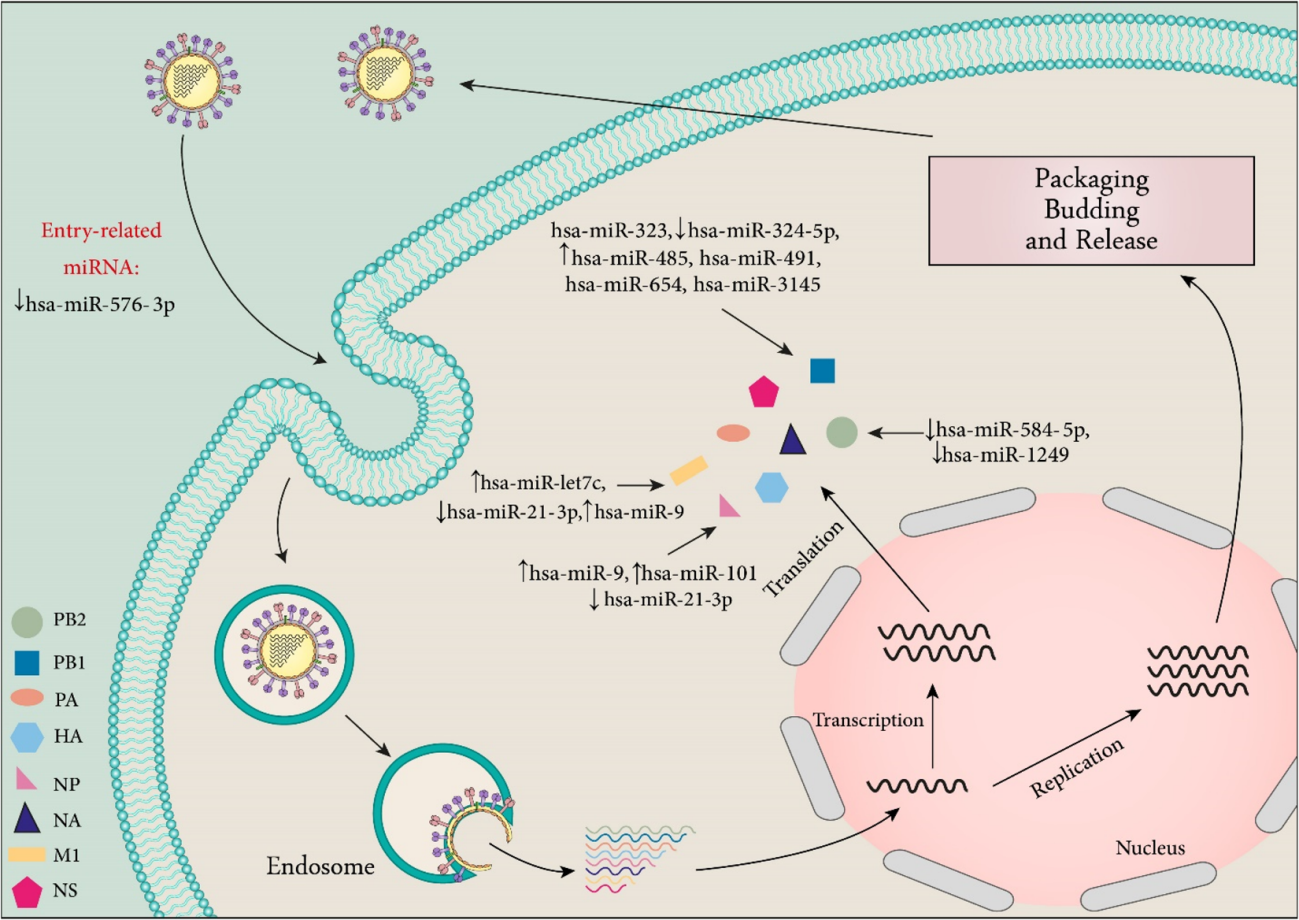
Summary of cellular miRNAs involved in IAV infection. Host miRNAs modulate the pathogenesis of IAV by targeting viral proteins (such as PB1, PB2, NP, and M1) that are essential for influenza replication and translation. miRNA dysregulation is indicated by an up or down arrow. [Color figure can be viewed at wileyonlinelibrary.com]

In a study performed by Li et al., miR‐HA‐3p was identified as the first miRNA‐like functional RNA fragment encoded by the H5N1 influenza virus. This viral miRNA suppresses the expression of the PCBP2. H5N1 infection induced suppression of the PCBP2 which increases cytokine production in infected mice and human macrophages. These findings show the role of this miRNA as an important virulence factor.^193^ Additional miRNAs are mentioned in Table 3 and summarized in Figure 7.

**Table 3:** miRNAs and their contribution to IAV infection.

miRNA	Strain	Expression	Target/human or virus	Effect	References
hsa‐miR‐21‐3p	H5N1	Downregulated	EGF2/Human	Downregulation of the miRNA reduces viral M1, NP, and IAV replication, while overexpression of the hsa‐miR‐21‐3p increases M1, NP and decrease type I IFN response and EGF2, promotes viral replication.	194
hsa‐miR‐21‐3p	H1N1, H5N1	Downregulated	HDAC8, FGF2/Human	Suppressing HDAC8 expression leads to promoting IAV replication. Overexpression aggravates H5N1 replication by downregulating FGF2.	194, 195
hsa‐miR‐26a	H1N1	Downregulated	USP3/Human	Downregulation increases USP3 and reduces IFN responses and IFNα/β production, enhance viral replication. Overexpression inhibits viral expression.	196
hsa‐miR‐29a	H1N1	Downregulated	FZD5/Human	Overexpression decreases viral production, protein, and mRNA. Overexpression reduces FZD5 protein levels.	197
hsa‐miR‐30 family	H1N1, H5N1, H9N2	Downregulated	SOCS1, SOCS3/Human	Overexpression inhibits IAV proliferation, reliefs inhibition of the IFN/JAK/STAT signaling pathway by reducing SOCS1 and SOCS3, inhibits NEDD4 expression.	186
hsa‐miR‐34a	H1N1, H3N2	Downregulated	BAX/Human	Downregulation upregulates BAX and promotes virus‐induced apoptosis.	198
hsa‐miR‐93	H1N1	Downregulated	JAK1/Human	Downregulation of hsa‐miR‐93 upregulates JAK1 and suppress IAV infection.	187
hsa‐miR‐221	H1N1	Downregulated	SOCS1/Human	Downregulation targets SOCS1/NF‑κB pathway which leads to suppressing type‑I IFN response and enhances IAV replication.	199
hsa‐miR‐302c	H3N2	Downregulated	NIK/Human	Downregulation of the miRNA upregulates NIK expression and activation of IFNβ expression, inhibits viral replication.	200
hsa‐miR‐324‐5p	H5N1	Downregulated	PB1/Virus CUEDC2/Human	Overexpression of the miRNA inhibits H5N1 replication by targeting the PB1 viral RNA, targets 3′UTR of the CUEDC2 and enhances antiviral immune responses, downregulation increases H5N1 replication.	201
hsa‐miR‐548an	H1N1, H3N2	Downregulated	NS1ABP/Human	Downregulation of the miRNA increases mRNA and protein levels of NS1ABP, overexpression of NS1ABP enhance viral maintenance, reduces apoptosis of infected cells, increases viral particle assembly and replication.	202
hsa‐miR‐576‐3p	H1N1	Downregulated	AP1G1/Human	Downregulation increases AP1G1 and could affect viral entry.	203
hsa‐miR‐584‐5p/1249	H5N1	Downregulated	PB2/Virus	Targets viral genome and downregulates PB2 expression, IAV‐induced downregulation of the miRNAs promotes viral replication.	191
hsa‐miR‐4276	H1N1, H3N2	Downregulated	COX6C/Human	Downregulation increases expression of COX6C, induce the apoptotic protein caspase‐9 and repress viral replication.	204
hsa‐miR‐let7c	H1N1	Upregulated	M1/Virus	Inhibits viral M1 vRNA synthesis, reduces IAV replication.	205
hsa‐miR‐9	H1N1, H3N2	Upregulated	MCPIP1/Human	Overexpression of the miRNA increases viral M and NP mRNA expression, promotes IAV gene expression and replication. Increases the expression of MCPIP1 which may have an antiviral role.	206
hsa‐miR‐33a	H1N1,H3N2, H9N2	Upregulated	ARCN1/Human	Inhibits ARCN1, weakens viral ribonucleoprotein activity and inhibits IAV replication.	207
hsa‐miR‐101	X‐31 virus (H3N2)	Upregulated	mTOR/Human	Represses mTOR, in last stages of infection and suppresses viral replication, decreases the expression of the NP.	192
hsa‐miR‐132‐3p	H1N1	Upregulated	IRF1/Human	Targets IRF1 gene and suppresses type I IFN response, inhibits INFα and INFβ, promotes IAV replication.	208
hsa‐miR‐136	H5N1	Upregulated	IL‐6, IFN‐β, RIG‐I/Human	Increases IL‐6 expression, acts as an immune agonist of RIG‐I, accumulation of IL‐6 and IFN‐β, suppress IAV replication, increase antiviral host response.	209
hsa‐miR‐141	H5N1	Upregulated	TGF‐β2/Human	Suppresses the expression of TGF‐β2, affect the inflammatory processes by decreasing inflammation.	210
hsa‐miR‐155	H1N1	Upregulated	S1PR1/Human	Regulates cytokine expression by downregulating S1PR1.	211
hsa‐miR‐200c	H5N6	Upregulated	CNTN1/Human	CNTN1 enhances viral replication, hsa‐miR‐200c downregulates the expression of the CNTN1.	212
hsa‐miR‐664a‐3p	H7N9	Upregulated	LIF, NEK7/Human	miRNA has a potent antiviral effects, targets LIF and NEK7 which are pro‐inflammatory factors.	213
hsa‐miR‐188‐3p	H1N1, H5N6, H7N9	Not reported	PB2/Virus	Downregulates the expression of the PB2 at mRNA and protein levels and suppresses the replication of the IAV.	214
hsa‐miR‐323, hsa‐miR‐491 hsa‐miR‐654	H1N1	Not reported	PB1/Virus	Binds and degrades the PB1 mRNA and inhibits replication of IAV.	185
hsa‐miR‐3145	H1N1, H3N2, H5N1	Not reported	PB1/Virus	Inhibits expression of the PB1 and inhibits viral replication.	215

### 5.2 Coronaviruses

Coronaviruses a diverse group of viruses with the ability of infecting humans and animals are a member of the *Coronaviridae* family. Human infecting CoVs can cause mild to severe respiratory diseases including pneumonia. Due to the absence of a serious threat, CoVs were not categorized as highly pathogenic infections. In 2002 and 2012, the outbreak of severe acute respiratory syndrome (SARS‐CoV) and Middle East respiratory syndrome (MERS‐CoV) as fatal respiratory illnesses, led to high mortality rates all around the world. Both SARS‐CoV and MERS‐CoV are believed to have originated in bats and are directly transferred to humans through market civets and dromedary camels, respectively.^216^ In 2019, SARS‑CoV‑2 another infectious agent of *Coronaviridae* family, with the ability to involve the lower respiratory tract, was identified worldwide and led to another pandemic and high mortality disease, known as COVID‐19, all around the world.^217^

**Figure 8:**
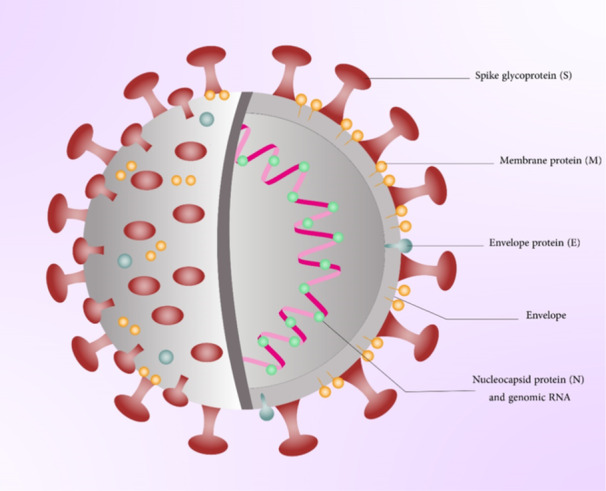
Schematic representation of SARS‐CoV‐2 virus structure. [Color figure can be viewed at wileyonlinelibrary.com]

SARS‑CoV‑2 has a ssRNA genome with a length of 29,881 bp^218^ (Figure 8). The genome of MERS‐CoV and SARS‐CoV shows 79% and 50% similarity to the SARS‐CoV‐2 genome, respectively.^219^ This RNA genome has the ability to encode structural proteins and non‐strucrural proteins. The genomic organization has six ORFs including envelope (E), nucleocapsid (N), membrane (M), spike (S), and replicase (ORF1a/ORF1b). Furthermore, accessory proteins are encoded by seven putative ORFs present between structural genes.^219^ More than two thirds of the viral genome is covered by the replicase gene. This gene is responsible for encoding a large polyprotein (pp1ab). Proteolytically cleavage of this polyprotein leads to 16 putative non‐structural proteins, which play an important role in viral replication and transcription. Some examples of these proteins are nsp13 (helicase), nsp12 (RNA‐dependent RNA polymerase, RdRp), nsp5 (main protease), and nsp3 (papain‐like protease).^220^

The replication cycle of SARS‐CoV‐2 initiates with endocytosis of the virus into human cells through attaching of spike protein to the ACE2 receptor of the host intestine, kidney, heart, lungs, vessels, and brain.^221^ Cleavages at the S2′ site and also at the junction of the S1 and S2 subunits of spike proteins are necessary for viral entry. SARS‐CoV‐2 has two distinct entry pathways: endosomal entry and cell surface entry. Clathrin‐mediated endocytosis occurs if the target cell lacks sufficient TM protease serine 2 (TMPRSS2) or if the virus–ACE2 complex does not encounter TMPRSS2. In this case, cathepsin cleavages S2′ in endolysosome. Conversely, TMPRSS2 induces cleavage of S2′ at the cell surface. Both routes lead to viral entry.^222^ SARS‐CoV also utilizes ACE2 receptors; however, SARS‐CoV‐2 binds to ACE2 receptors with higher affinity than SARS‐CoV.^223^ After the entry, the viral genome releases into the host cell cytoplasm, which leads to the translation of ORF1a and ORF1b (two large open reading frames) into pp1a and pp1ab polypeptides, respectively. Co‐translationally and post‐translationally processing of these polypeptides leads to the formation of 16 NSPs and a replication/transcription complex (RTC).^224^ This 16 NSPs, after translation from pp1a (nsp1–11) and pp1ab (nsp1–10, nsp12–16) by two cysteine proteases located inside nsp3 and nsp5, are affected by proteolytic cleavage and released. The nsp12 inside RdRp is the main component of RTC, which performs RNA synthesis. The cofactors of nsp12 for RNA production are nsp7 and nsp8.^225^ In the process of negative‐strand RNA production, the RTC encounters transcription regulatory sequences (TRSs), which are found upstream of the majority of ORFs in the 3′ one third of the viral genome, and RTC prevent transcription. Further, structural proteins are produced, translocated to the ER membrane, and transited through ERGIC.^224^ In the last step, virions leave infected cells via exocytosis. Generally, the exocytosis mechanisms of coronaviruses from the cell are assembly and budding of synthesized virions at the ER‐to‐Golgi compartment.^226^ However, the latest studies indicate that SARS‐CoV‐2 and other betacoronaviruses utilize lysosomal trafficking pathway to exit infected cells.^227^

#### 5.2.1 Role of miRNAs in coronavirus infection

Coronavirus genome contains 5′‐UTR and 3′‐UTR regions. These regions are important in coronavirus pathogenesis, transcription, and replication.^228^ Also miRNAs bind to these regions to perform their negative regulatory function.^229^ In a meta‐analysis study investigating 39 miRNAs, a significant decline was identified in the expression of hsa‐miR‐5004‐3p. Mining miRNAs identified this miRNA as the only miRNA that can target the leader sequence of SARS‐CoV‐2 and SARS‐CoV. The study reported that SARS‐CoV‐2 suppressed binding of this miRNA to viral 5′‐UTR by insertion‐type mutation which decreased the stability of miRNA binding. This process allowed SARS‐CoV‐2 to escape from the effect of immunity system‐related miRNAs.^230^

SARS‐CoV‐2 infection leads to disturbance in the regulation of pro‐inflammatory cytokines and causes an increase in the release of pro‐inflammatory cytokines such as IL‐6, TNF‐α, IL‐2R, and IL‐8 as a cytokine storm, resulting clinical symptoms exacerbation.^231^, ^232^ It is worth noting that IL‐8 and IL‐6 are regulated by miRNAs.^233^ Lung biopsies of SARS‐CoV‐2 infected patients identified reduced expression of hsa‐miR‐34a‐5p, hsa‐miR‐29b‐3p, and hsa‐miR‐26a‐5p. Besides, the correlation between these miRNAs, cytokine storm, expression of inflammatory biomarkers, and endothelial dysfunction was observed. In selected COVID‐19 patients, IL‐4 levels were evaluated and showed an inverse relation with expression of hsa‐miR‐29b‐3p. However, IL‐8 levels were increased proportionally to elevated expression of hsa‐miR‐29b‐3p. Also, IL‐6 and hsa‐miR‐26a‐5p indicated the same inverse correlation.^234^ Enrichment analysis showed that hsa‐miR‐34a‐5p as one of the miRNAs with highest number of potential target sites in SARS‐CoV‐2 RNA, although these predicted target sites must be validated using functional experiments.^235^ As CASP‐1 is the direct gene target of this miRNA, the cellular processes mediated by the CASP‐1 were potentially impacted by this miRNA. Decreased expression of hsa‐miR‐34a‐5p and increased expression of CASP‐1 were observed in COVID‐19 patients. Taken together, in SARS‐CoV‐2 infected patients above‐mentioned miRNAs have a role in inflammatory response.^234^

In a study, miRNA database searching and target prediction introduced viral RdRp as a target of the hsa‐miR‐15b‐5p which suggested this miRNA as an anti‐SARS‐CoV‐2 miRNA. In vitro RNA‐RNA binding assay demonstrated that hsa‐miR‐15b‐5p directly interacts with SARS‐CoV‐2 RdRp structure. This study indicated the potential biomarker role of the hsa‐miR‐15b‐5p in the treatment of the SARS‐CoV‐2 infection.^236^

Cytokine storm as dysregulation of pro‐inflammatory cytokines is seen in considering hospitalized SARS‐CoV‐2 infected patients.^237^ One of the host‐directed therapies in COVID‐19 treatments is utilizing Tocilizumab (TCZ) as monoclonal antibody against IL‐6.^238^ COVID‐19 patients show different responses to TCZ treatment.^239^ Some miRNAs, including hsa‐miR‐126‐3p, hsa‐miR‐21‐5p, and hsa‐miR‐146a, which are also known as inflamma‐miRs, have the ability to regulate inflammation and they can target molecules related to the NF‐κB pathway.^240^ In a study by Sabbatinelli et al., the serum levels of these miRNAs examined in tocilizumab received COVID‐19 infected 29 subjects with age‐ and sex‐matched healthy control. The serum levels of hsa‐miR‐146a‐5p were reduced, and IL‐6 was increased in COVID‐19 patients. Patients who did not respond to TCZ had the lowest serum levels of hsa‐miR‐146a‐5p after the treatment and experienced severe consequences.^241^

The potential targets of 100 miRNAs were identified on the SARS‐CoV‐2 RNA sequence only 15 miRNAs were predicted to target viral RNA. Among these miRNAs, five miRNAs including hsa‐miR‐1207‐5p, hsa‐miR‐4763‐3p, hsa‐miR‐103a‐3p, hsa‐miR‐6821‐5p, and hsa‐miR‐6089 were predicted to have a common target located in S glycoprotein viral sequence. Also, the binding site of the hsa‐miR‐130a‐3p was determined in N protein coding region. hsa‐miR‐4763‐3p, hsa‐miR‐6821‐5p, and hsa‐miR‐6089 had a binding site in Nsp13, Nsp12, and Nsp10 coding regions in ORF1ab gene. In normal lung tissues, hsa‐miR‐1207‐5p showed high expression levels and displayed an important role in inflammation by direct targeting of colony‐stimulating factor 1 (CSF1). Furthermore, this miRNA was introduced as a negative regulator of epithelial‐to‐mesenchymal transition (EMT). SARS‐CoV‐2 infection results in overexpression of CSF1 mRNA and genes involved in EMT. In COVID‐19 patients, hsa‐miR‐1207‐5p has the possibility of interacting with the viral genome. This interaction can result in the deregulation of CSF‐1 which leads to promoting EMT, enhancing inflammatory responses, and pulmonary fibrosis in SARS‐CoV‐2 infected patients.^242^

Computational prediction led to the identification of six miRNAs with high binding possibility across human coronaviruses. Among these miRNAs, hsa‐miR‐21‐3p binds to human coronaviruses with more possibility and it was identified as a potential regulator of most coronaviruses including, SARS‐CoV and SARS‐CoV‐2. In SARS‐CoV‐infected mouse, hsa‐miR‐21‐3p was dramatically upregulated in the lungs of the infected mouse. Considering the high structural similarity of SARS‐CoV and SARS‐CoV‐2 and the common binding sites of this miRNA, hsa‐miR‐21‐3p can be utilized in understanding COVID‐19 pathogenesis.^243^

TMPRSS2, as a serine protease of human cells, has a pivotal role in the pathogenesis of SARS‐CoV‐2 by mediating fusion between the viral envelope and plasma cell membrane.^244^ Utilizing three prediction algorithms led to the selection of three TMPRSS2 targeting miRNAs from a pool of 163 miRNAs. Three highly specific potent hsa‐miR‐32, hsa‐miR‐98, and hsa‐miR‐214 were identified with strong binding affinity. Transfection of these three miRNAs suppressed Tmprss2 at gene and protein levels. Overexpression of these miRNAs potentially can regulate viral entry.^245^ hsa‐miR‐98‐5p as a highly conserved miRNA has potential of targeting TMPRSS2 mRNA and repressing its expression. In an in vitro study of endothelial cells, hsa‐miR‐98‐5p targeted 3′‐UTR gene transcription of TMPRSS2, which may have a potential role in fighting against COVID‐19.^246^

**Figure 9:**
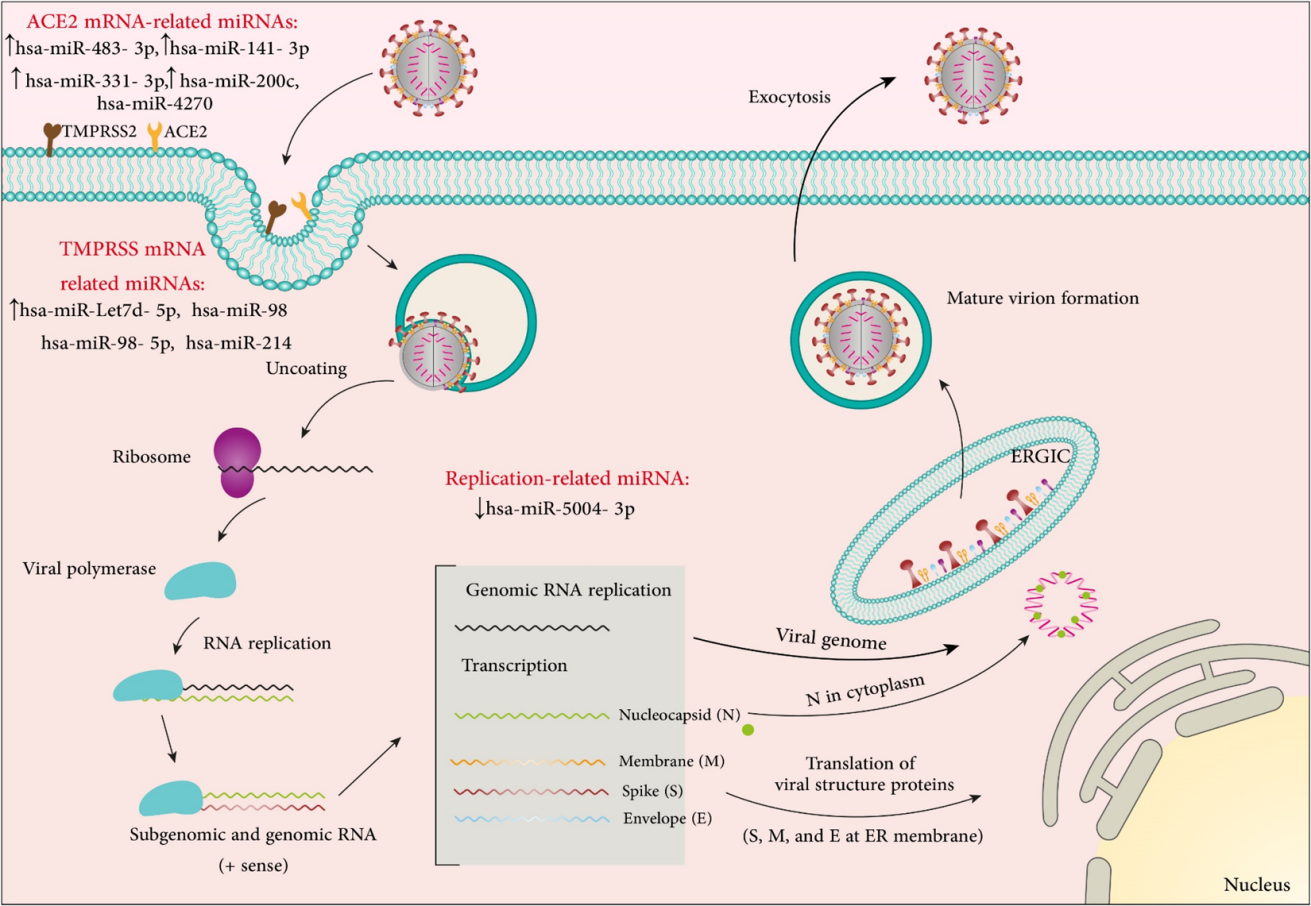
SARS‐CoV‐2 replication cycle and involved miRNAs. ACE2 and TMPRSS have an important role in viral fusion with plasma cell membrane and its entry. miRNAs related to ACE2, TMPRSS, and viral replication are shown. miRNA dysregulation is indicated by an up or down arrow. [Color figure can be viewed at wileyonlinelibrary.com]

Additional miRNAs involved in coronavirus pathogenesis are mentioned in Table 4 and summarized in Figure 9.

**Table 4:** miRNAs and their predicted contribution to SARS‐CoV‐2 infection.

miRNA	Cell line	Expression	Target/human or virus	Effect	References
hsa‐miR‐483‐3p	Calu‐3	Upregulated	ACE2 mRNA/Human	RNAseq data showed upregulation of miR‐483‐3p which targets ACE2 mRNA, inhibit SARS‐CoV‐2 entry.	247
hsa‐miR‐let‐7d‐5p	Human lung tissue	Upregulated	TMPRS2 mRNA/Human	RNA‐sequencing datasets showed upregulation of miRNA, predicted to targets TMPRS2 mRNA, upregulation inhibits SARS‐CoV‐2 entry.	247
hsa‐miR‐141‐3p, hsa‐miR‐4270, hsa‐miR‐331‐3p	Human lung tissue	Upregulated	ACE2 mRNA/Human	RNA‐sequencing datasets showed upregulation of miRNAs, predicted to target ACE2 mRNA, inhibits viral entry.	247
hsa‐miR‐200c	Rat primary cardiomyocytes, human iPSC‐derived cardiomyocytes	Upregulated	ACE2 mRNA and protein/Human	Targets ACE2 in cardiomyocytes and inhibits mRNA and protein levels of ACE2.	248
hsa‐miR‐155	SARS‐COV‐2 infected patients, SARS‐CoV‐2‐infected transgenic ‐mice	Upregulated	Not reported	Could be a biomarker for the detection of COVID‐19 disease. Anti‐hsa‐miR‐155 in SARS‐CoV‐2‐infected transgenic ‐mice reduced levels of inflammatory mediators, and improved survival and body weight.	249, 250
hsa‐miR‐361‐5p	SARS‐CoV‐2‐resistant cell line A549 and lung tissue	Co‐expressed withhsa‐miR‐24‐3p and hsa‐miR‐143‐3p	IFN‐α mRNA/Human	hsa‐miR‐361‐5p predicted to have a binding site at IFN‐α 3′‐UTR.	247
hsa‐miR‐24‐3phsa‐miR‐143‐3p	SARS‐CoV‐2‐resistant cell line A549 and lung tissue	Co‐expressed withhsa‐miR‐361‐5p	IFN‐γ mRNA/Human	Predicted to target IFN‐γ mRNA.	247
hsa‐miR‐495‐3p	Resistant cell lines A549human primary lung fibroblasts	Upregulated	ORF1ab region of the SARS‐CoV‐2 genome/VirusIFN‐γ 3′‐UTR/Human	Predicted to target IFN‐γ 3′‐UTR.	247
hsa‐miR‐361‐5p	Calu3 and LF cell lines	High expression in cell lines resistant to SARS‐CoV‐2 infection, low expression in cell lines permissive to infection	IFN‐α/Human	Predicted to target IFN‐α mRNA.	247

## 6 SEXUALLY TRANSMITTED VIRAL INFECTIONS

Sexually transmitted viral infections are great public health issues worldwide and can cause chronic, lifelong infections, or cancer.^251^ This section describes pivotal sexually transmitted viral infections including human papillomavirus (HPV), human immunodeficiency virus (HIV), and herpes virus.

### 6.1 Human immunodeficiency virus

Infection with HIV has been a global public health issue in the last 20 years. Based on UNAIDS reports of 2019, HIV infection led to the death of 690,000 people, and nearly 38 million people are living with HIV. The ways of transmission and spread of HIV include sexual transmission, contaminated blood, use of shared injection needles, and transmission from infected mother to infant during pregnancy and delivery.^252^

HIV eliminates CD4+ T cells, which protect the body against infections, leads to the weakening of the host defense system.^253^, ^254^ The last advanced stage of this infection is AIDS (acquired immune deficiency syndrome) disease.^255^ HIV as a member of the *Retroviridae* family is classified into main HIV‐1 and HIV‐2 types. HIV‐1 has groups of M, N, O, and P, and HIV‐2 is divided into A–H groups. HIV‐1 is the most pathogenic type and the M group of HIV‐1 as the most common strain is the cause of the global HIV pandemic.^256^

**Figure 10:**
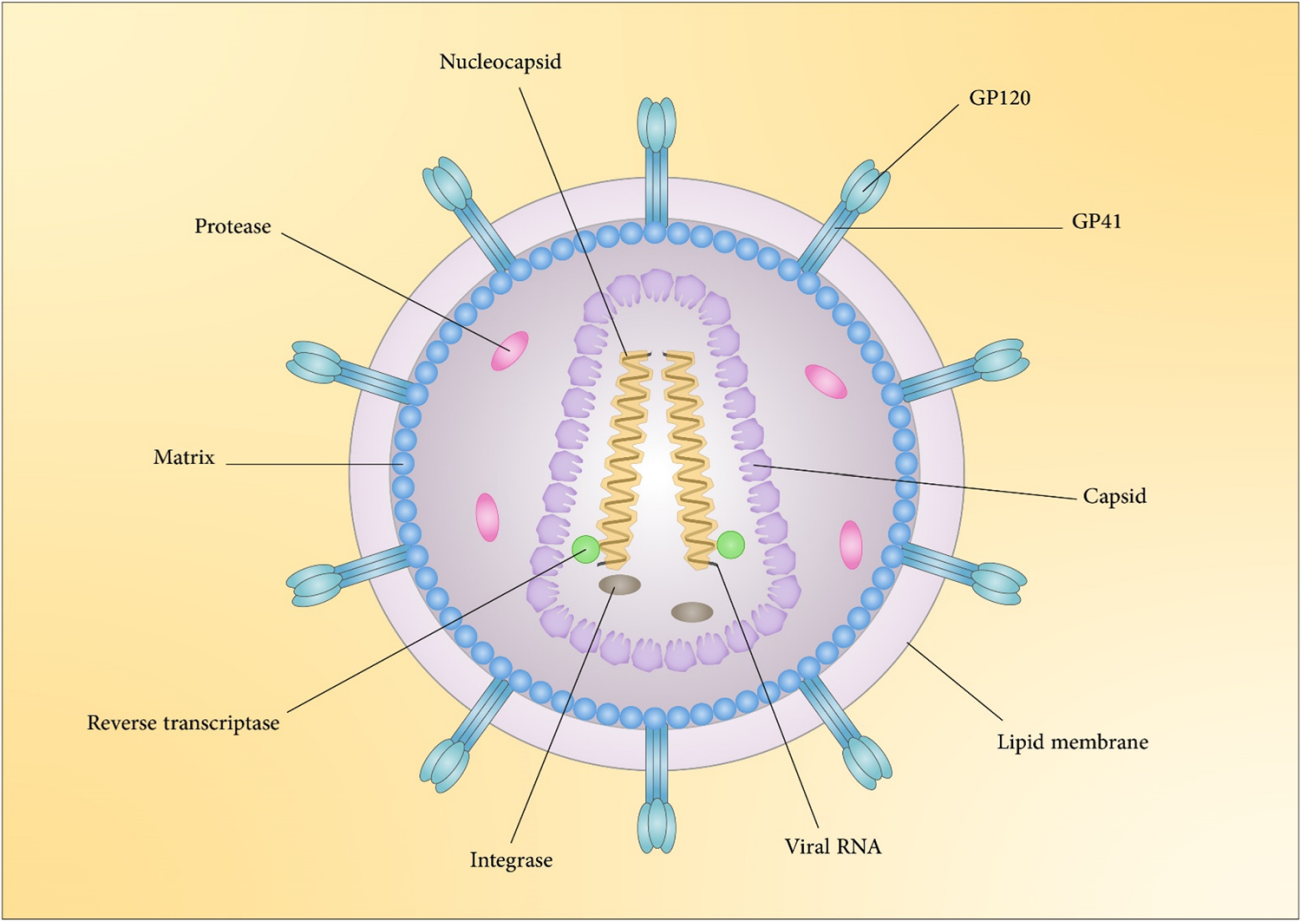
Schematic representation of HIV structure. [Color figure can be viewed at wileyonlinelibrary.com]

The genome of HIV is a ssRNA genome that contains nine genes and encodes 16 essential viral proteins. Viral envelope proteins including GP41 and GP120 (Figure 10), HIV enzymes including integrase, reverse transcriptase, protease, and also structural proteins including p6, nucleocapsid, capsid, and matrix are encoded from three major *env*, *pol*, and *gag* genes.^257^ Other viral genes are responsible for encoding structural proteins, including Nef, Vpr, Vpu (in HIV‐1)/Vpx (in HIV‐2), Vif, and regulatory proteins such as Rev and Tat.^258^

The replication cycle of HIV‐1 can be categorized into early and late phases. The early phase comprises attachment of the virus to the host cell surface and the integration of viral DNA into the host genome. Gene expression and releasing of new virions are steps of the late phase.^257^ Outermost envelop proteins and matrix protein p17 surround the core capsid structure of HIV‐1, which has two copies of the ssRNA.^259^ HIV replication cycle initiates with the entrance of the virus into host cells by recognizing CD4 receptors of the host cell surface through its glycoprotein GP120 as a structure of Env. Following this interaction, a conformational change occurs to GP120 protein, which leads to its interaction with CC‐chemokine receptor 5 (CCR5) and CXC‐chemokine receptor 4 (CXCR4) coreceptors. Besides, the viral protein GP41 leads to the fusion of the virion with plasma cell membrane and subsequently the release of viral capsid into cytoplasm. Reverse transcription begins in cytoplasm. Nuclear pore complexes (NPC) where the capsid docks, are responsible for transporting the capsid into the nucleus. Upon entry into the nucleus, the capsid partially uncoats, and reverse transcription of viral RNA into double‐stranded DNAs completes.^260^ Then, the viral DNA is integrated into the host's DNA genome by integrase, and as a result, the pro‐viral DNA is replicated along with the host's DNA.^261^, ^262^ In the next step, mRNAs are transcribed and transferred from the nucleus by Rev following the interaction between rev‐responsive element (RRE) in viral RNA and cellular Crm1. Finally, Gag and Gag‐Pol precursors multimerize through interaction with Gag proteins to produce new viral particles.^263^

#### 6.1.1 Role of miRNAs in HIV infection

hsa‐miR‐34a has already been shown to be upregulated during HIV‐1 infection and is involved in HIV replication.^264^ Also in neurons, Vpr and Tat proteins of HIV can increase the expression of hsa‐miR‐34a to perform HIV‐1‐induced dementia by causing apoptosis.^265^, ^266^ HIV‐1 utilizes host factors in its replication. For instance, Tat, responsible for the elongation of HIV transcripts, utilizes the host P‐TEFb cellular factor.^267^ Other multiprotein complexes including WDR82/PPP1R10/TOX3 interact with the P‐TEFb complex. As a nuclear protein phosphatase 1 (PP1), PPP1R10/PNUTS controls apoptosis and cell cycle progression.^268^ It was established that PNUTS/PPP1R10 is a target of hsa‐miR‐34a.^269^ In HIV‐1 infection, PNUTS/PPP1R10 interferes with the cyclin T1–CDK9 interaction and inhibits HIV‐1 transcription. Furthermore, it prevents the formation of a functional PTEFb complex and inhibits HIV‐1 replication. To overcome this inhibition, viral infection increases hsa‐miR‐34a expression. Progression of HIV‐1 infection leads to decreasing in PNUTS expression and promoting of HIV‐1 replication.^270^

Like CD4+ T cells, HIV infection can also target macrophages. However, infected macrophages show more resistance to virus‐mediated cytopathic effects than CD4+ T cells.^271^ They also play a role in viral spread to bone marrow, gut, lung, spleen, and brain.^272^ CD4 as a HIV‐1 essential receptor for viral entry is also being expressed in macrophages.^273^ Lodge et al. identified upregulation of hsa‐miR‐221 and hsa‐miR‐222 in bystander monocyte‐derived macrophages (MDMs). In MDMs, HIV infection enhanced the secretion of TNF‐α. Increased levels of TNF‐α mediated the enhanced expression of hsa‐miR‐222 and hsa‐miR‐221. These miRNAs directly reduced the expression of CD4, which reduced HIV‐1 entry in macrophages. They also limited production and propagation of HIV‐1 in MDM cultures.^274^

Chronic HIV‐1 infections result in functional exhaustion of immune cells. In acute infection, immune responses are reduced by negative regulation mechanisms including co‐inhibitory receptors cascade. HIV‐1 infection results in increased expression levels of hsa‐miR‐146a in the peripheral blood of infected patients. The increased levels of miRNA positively correlate with higher exhaustion markers. Furthermore, transfection of hsa‐miR‐146a mimic to CD3 antibody‐activated CD8+ T cells decreased protein and mRNA levels of TNF‐α, IL‐2, and IFN‐γ. This demonstrated that hsa‐miR‐146a may decrease the production of antiviral cytokines and reduce cellular cytotoxicity which may result in negative regulation of CD8+ T cells function. Also, c‐Fos protein levels were slightly decreased in the peripheral blood of infected patients. Immune exhaustion might be caused by hsa‐miR‐146a via indirect suppressing of c‐Fos. Taken together, hsa‐miR‐146a inhibits the antiviral function of immune cells.^275^

It was demonstrated that HIV infection induces expression of the hsa‐miR‐210‐5p. Also, HIV infection downregulates the expression of the TGIF2 as the target of the hsa‐miR‐210‐5p by upregulating this miRNA. Furthermore, the viral Vpr can also induce G2/M arrest of the cell cycle through upregulating of the miRNA via enhancing the activity of the p50 which is an NF‐κB transcription factor. Taken together, HIV infection and the viral Vpr can induce cell cycle arrest by upregulating hsa‐miR‐210‐5p.^276^

In a study in HIV‐1 productively infected cells and C11 cells, Wang et al. identified differentially expressed hsa‐miR‐1290 and hsa‐miR‐196b. These miRNAs play a role in the modulation of HIV‐1 latency and affect the HIV‐1 expression by targeting 3′‐UTR of HIV‐1 which leads to suppression of infectivity and production of HIV‐1.^275^ In another study, it was observed that viral Tat protein which regulates the elongation of HIV‐1 transcripts, increased the expression of hsa‐miR‐21 and hsa‐miR‐222. These miRNAs participate in apoptosis and cell cycle. HIV‐1 induced upregulation of these miRNAs downregulates PTEN, PDCD4, and CDKN1B. In CD4+ T cells, upregulation of these miRNAs interferes with the PTEN‐AKT‐FOXO3a signaling pathway and protects against apoptosis which may lead to maintaining efficient replication and HIV‐1 pathogenesis.^277^

HIV can be incorporated into the airway epithelial cells of humans.^278^ Pulmonary complications are one of the significant mortality reasons in patients infected with HIV.^279^ Nuclear factor (erythroid‐derived 2)‐like 2 (Nrf2) controls barrier function of alveolar epithelial cell (AEC) and oxidative defense. The expression of HIV‐1 in transgenic rats inhibits Nrf2. In primary alveolar epithelial cells (AECs) obtained from HIV‐1 transgenic (HIV‐1 Tg) rats, a significant increase in hsa‐miR‐144 levels was observed. hsa‐miR‐144 has a binding site in 3′‐UTR of Nrf2 mRNA. Increased expression of hsa‐miR‐144 impairs epithelial barrier function by inhibiting barrier formation by decreasing the activity and expression of Nrf2 and its downstream effectors. This increased expression of hsa‐miR‐144 leads to the inhibition of Nrf2‐regulated critical defense pathways. Furthermore, utilizing hsa‐miR‐144 antagomir increased the levels of Nrf2 which proposes that lung health in HIV patients can increase by targeting the inhibition of Nrf2.^280^

#### 6.1.2 Viral miRNAs

In a study conducted by Omoto et al., they found the possibility of producing nef‐derived miRNAs in HIV‐1‐infected cells. They found that HIV‐infected cells produce *nef*/U3 miRNAs which can suppress HIV‐1 virulence and Nef function.^281^

**Figure 11:**
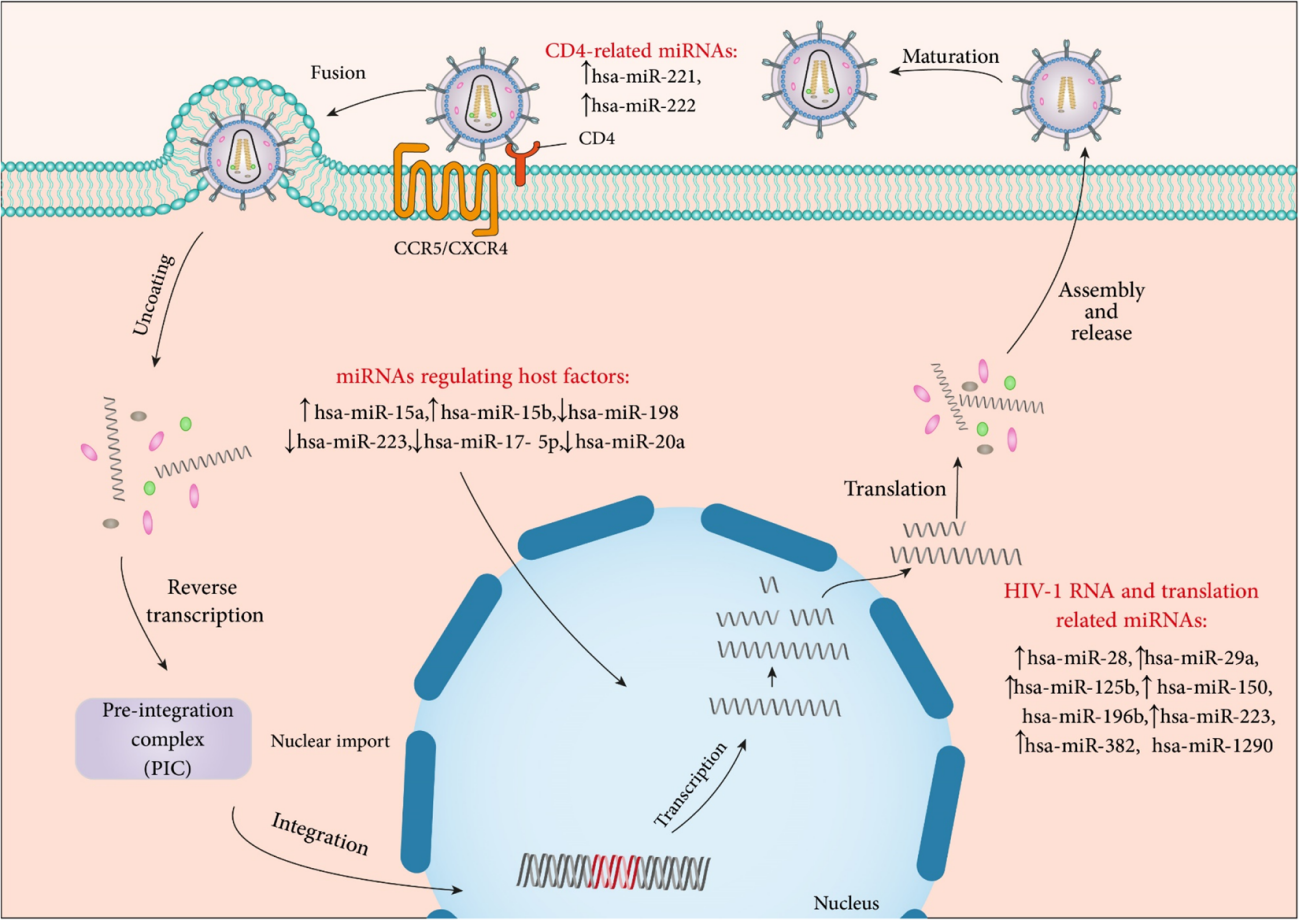
Summary of cellular miRNAs involved in HIV infection. hsa‐miR‐221 and hsa‐miR‐22 regulate the expression of CD4 which is an essential receptor for HIV entry. Some microRNAs are involved in HIV pathogenesis by regulating host factors. hsa‐miR‐15a and hsa‐miR‐15b regulates Pur‐α, hsa‐miR‐198 and hsa‐miR‐223 regulates cyclin T1, hsa‐miR‐17‐5p and hsa‐miR‐20a regulates PCAF. microRNAs regulating HIV1 RNA and translation are also shown in the figure. miRNA dysregulation is indicated by an up or down arrow. [Color figure can be viewed at wileyonlinelibrary.com]

miR‐H3, as another HIV‐1 encoded miRNA, showed a replication‐enhancing function. Overexpression of miR‐H3 increases viral production, protein expression, and HIV‐1 RNA transcription. Furthermore, miR‐H3 upregulates the promoter activity by targeting HIV‐1 5′ LTR and activates the viral transcription by interacting HIV‐1 5′ LTR located TATA box. As a replication‐enhancing miRNA, miR‐H3 enhances viral production.^21^ Also, the TAR element located in the 5′‐end of the HIV derived transcripts encodes miR‐TAR‐5p and miR‐TAR‐3p.^282^ Table 5 contains additional involved miRNAs, and Figure 11 summarizes miRNAs involved in HIV pathogenesis.

**Table 5:** miRNAs and their contribution to HIV infection.

miRNA	Cell	Expression	Target/human or virus	Effect	References
hsa‐miR‐let‐7c, hsa‐miR‐34a‐5p, or hsa‐miR‐124a	HeLa‐CCR5, JLTRG‐R5	Upregulated	p21 and TASK1/Human	Downregulates p21 and TASK1, increases virion release, viral genome transcripts, and increased HIV‐1 replication.	283
hsa‐miR‐28, hsa‐miR‐125b, hsa‐miR‐150, hsa‐miR‐223, hsa‐miR‐382	Resting CD4+T cells	Upregulated	3′ ends of HIV‐1 mRNA/Virus	Combination of inhibitors of these miRNAs increased HIV‐1 production and the expression of various HIV‐1 proteins.	284
hsa‐miR‐155	‐	Upregulated	TRIM32/Human	Inhibits the HIV‐activating effects of TRIM32 and promotes the return of virus to latency.	285
hsa‐miR‐29a	Human T lymphocytes	Upregulated	3′‐UTR region of HIV‐1/Virus	Suppressed viral replication.	286
hsa‐miR‐1236	Monocyte	Not reported	VprBP (Vpr (HIV‐1)‐binding protein)/Human	Overexpression of the miRNA represses transcription of VprBP in monocytes, represses HIV‐1 infection.	287
hsa‐miR‐132	Activated CD4+ T cell, Jurkat T cells	Upregulated	MeCP2/Human	Overexpression of exogenous miRNA increases HIV‐1 replication in Jurkat T cells. Knockdown of MeCP2 (hsa‐miR‐132 target) increases HIV replication.	288
hsa‐miR‐103	MDMs	Upregulated	CCR5/Human	Inhibits CCR5‐dependent HIV‐1 entry by reducing the expression of CCR5.	289
hsa‐miR‐144	Primary rat alveolar macrophage, human MDMs	Upregulated	Nrf2/Human	Inhibits the expression of the Nrf2, impairs macrophage Phagocytosis. Silencing of the miRNA can improve lung health of HIV‐infected patients.	290
hsa‐miR‐186, hsa‐miR‐210, hsa‐miR‐222	Sup‐T1 cells	Upregulated	Dicer1, HRB, HIV‐EP2/Human	Downregulates Dicer1, HRB, HIV‐EP2, inhibit viral replication.	
hsa‐miR‐15a, hsa‐miR‐15b, hsa‐miR‐16, hsa‐miR‐20a, hsa‐miR‐93, hsa‐miR‐106b	Monocyte	Upregulated	3′UTR of Pur‐α/Human	miRNAs repress translation of Pur‐α mRNA and impair HIV‐1 replication.	291
hsa‐miR‐17‐5p, hsa‐miR‐20a	Jurkat cells	Downregulated	Histone acetyltransferase PCAF/Human	Exogenous overexpression suppresses HIV‐1 replication and production by reducing PCAF expression. Inhibition of endogenous versions enhanced PCAF and HIV‐1 replication.	292
hsa‐miR‐27b	Activated CD4+ T cells	Downregulated	Cyclin T1 3′UTR/Human	Directly regulate the expression of cyclin T1, downregulation of miRNA increases cyclin T1 levels. Overexpression of miRNA decreases cyclin T1 protein levels and decreased viral gene expression.	293
hsa‐miR‐29b, hsa‐miR‐150, hsa‐miR‐223	Activated CD4+ T cells	Downregulated	Cyclin T1 3′UTR/Human	Indirectly regulate the expression of cyclin T1, downregulation of miRNA increases cyclin T1 levels. Overexpression of miRNAs decreases cyclin T1 protein levels.	293
hsa‐miR‐125b	HEK‐293T, SupT1, THP1 cells	Downregulated	CPSF6/Human	HIV infection induces downregulation of the miRNA which is related to HIV‐1 reverse transcription and upregulation of the CPSF6. Decreased expression of the miRNA enhances nuclear entry of HIV‐1.	294
hsa‐miR‐34c‐5p	CD4+ T‐cells, Jurkat T cells	Downregulated	‐	Alters the expression of several genes involved in cell activation and TCR signaling, downregulates during HIV infection and functions in antiviral host response, overexpression promotes HIV‐1 replication.	295
hsa‐miR‐181a	Astrocytes	Downregulated	SAMHD1/Human	Downregulation of hsa‐miR‐181a increases SAMHD1. Overexpression of miRNA decreases SAMHD1 and increases viral replication.	296
hsa‐miR‐198	Monocytes	Downregulated	3′UTR of Cyclin T1 mRNA/Human	Cyclin T1 levels increase in downregulation of hsa‐miR‐198 in primary monocytes. Overexpression of hsa‐miR‐198 in differentiating monocytes decreases upregulation of cyclin T1, represses HIV‐1 replication and proviral gene expression.	297
hsa‐miR‐29a	HeLa, HEK293T and Jurkat	Expressed more in Hela cells	Nef/Virus	Impairs HIV‐1 replication by downregulating Nef proteins expression.	298
hsa‐miE‐196b, hsa‐miR‐1290	C11 cells, productively infected cells, and Jukrat	Differentially expressed	3′UTR of HIV‐1/Virus	Ectopic expression of miRNA suppressed HIV‐1 production and infectivity.	275

### 6.2 Human papillomavirus

HPV infection is one of the prevalent sexually transmitted diseases, which has mucosal and cutaneous transmission, and it can be transmitted by mucosa‐to‐mucosa or skin‐to‐skin contact.^299^ HPV infection leads to benign protruding or flat warts, anogenital cancers such as cervical, vaginal, and anal cancer, head and neck cancers, and other malignancies.^300^, ^301^ According to WHO report, 99% of cervical cancer cases are related to HPV infection, which led to the death of 342,000 women in 2020.^302^, ^303^ To date, HPV has more than 200 types, which are categorized into α, β, γ, μ, and ν genotype genera.^304^ HPV α genera as the largest group mainly contain mucosal epithelia infecting HPVs. The members of this group have the ability of infecting the anogenital tract. High‐risk (HR) types lead to anogenital cancers and are classified as oncogenic and categorized into HPV α genera.^305^ HPV‐16 and HPV‐18 of the HR type account for approximately 60% and 15% of worldwide cervical cancer, respectively.^305^, ^306^

**Figure 12:**
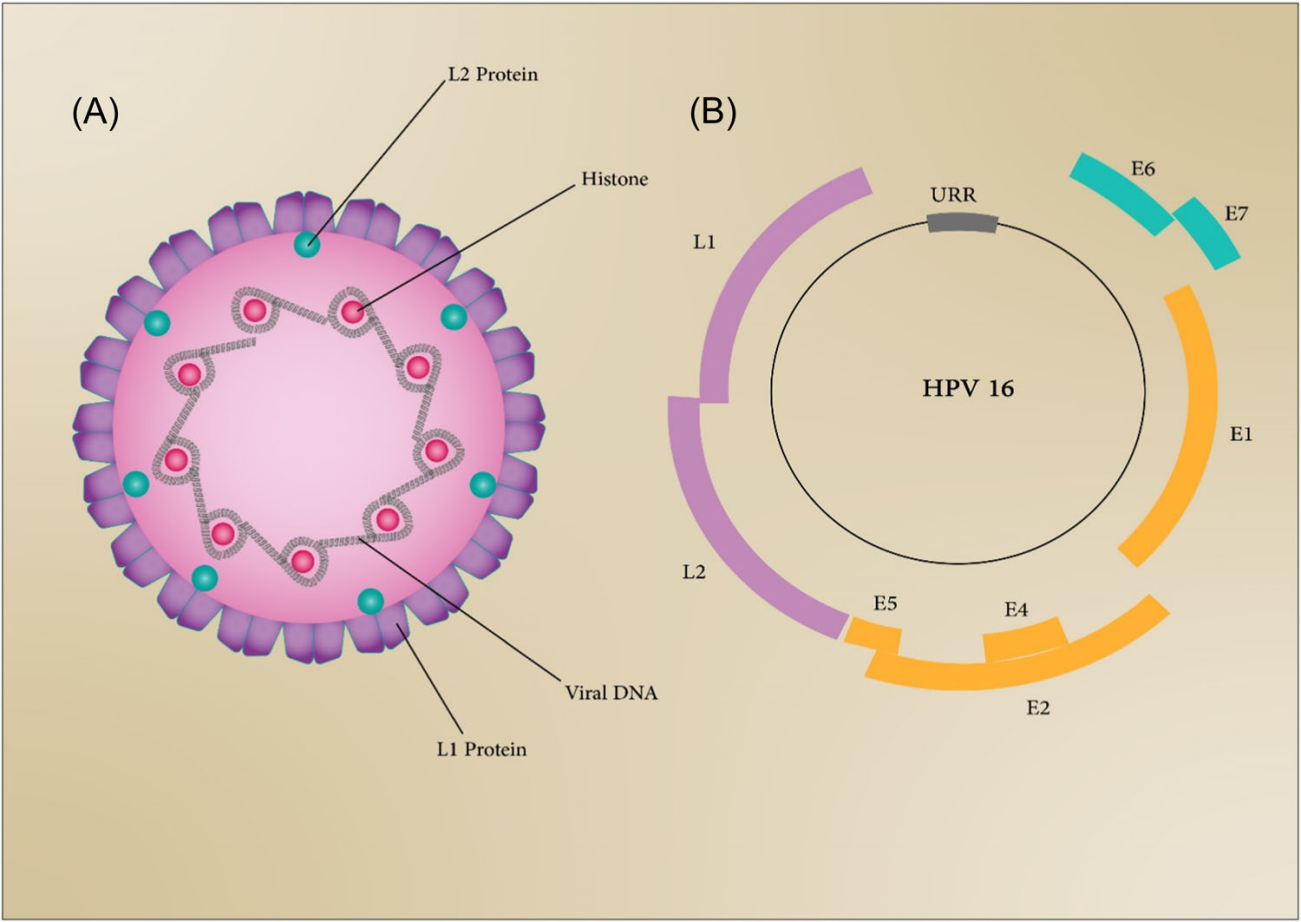
(A) Schematic representation of HPV structure. (B) Organization of the HPV16 genome. [Color figure can be viewed at wileyonlinelibrary.com]

HPV as a member of the *Papillomaviridae* family, is a nonenveloped DNA virus. The ds circular genome of HPV is about 8000 bp in size. The HPV genome contains early and late genes, as well as the upstream regulatory region (URR). Transcription factor binding sites and the origin of the replication site (ori) are in the URR region.^307^ Early genes are E1, E2, E4, E5, E6, and E7 and late genes are L1 and L2.^308^ The HPV genome encodes E1 and E2 which control HPV transcription, replication, and functions as regulatory proteins. E5, E6, and E7 proteins have an important role in immune evasion, viral replication cycle, and malignancy.^309^ Also, the HPV genome encodes capsid‐forming L1 and L2 structural proteins. The gene of E4 proteins encodes divergent and highly expressed late protein (Figure 12).^304^, ^310^

**Figure 13:**
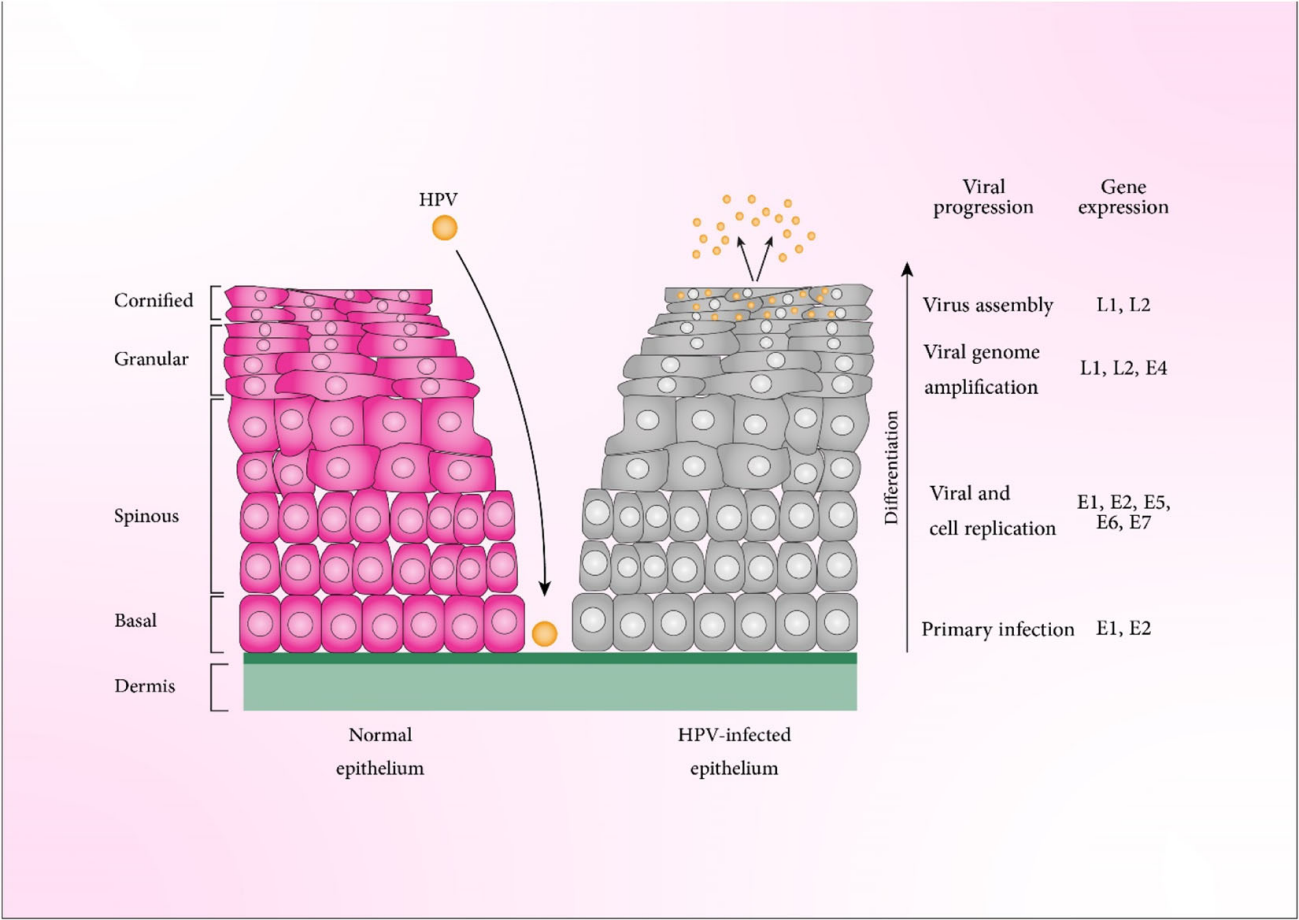
The replication cycle, viral progression, and expressed genes of human papillomavirus. [Color figure can be viewed at wileyonlinelibrary.com]

HPVs only infect cutaneous and mucosal epithelia and their replication cycle is linked to the differentiation of epithelial cells. To reach proliferating keratinocytes, HPV binds to the basement membrane of the epithelium (Figure 13).^311^ In this binding, L1 protein of viral capsid binds to sulfate proteoglycans (HPSGs). This binding leads to a conformational change in which the amino terminus of L2 minor capsid protein is exposed for cleavage by furin. This cleavage is essential for virus uptake and internalization.^312^ Following furin cleavage, RG‐1 epitope on L2 and allosteric L1‐mediated secondary binding events to transfer HPVs from basement membrane to the cell surface.^313^, ^314^ After the L1‐mediated endosomal uptake and entry of the virus by retrograde trafficking, L1 is degraded and HPV utilizes retromer to move toward the nucleus. L2 remains associated with viral DNA and mediates the escape to *trans*‐Golgi network and ensures the correct nuclear entry.^315^, ^316^ Cellular karyopherin mediates nuclear entry of L2 and L1.^317^ Viral E1 and E2 proteins are among the first expressed proteins. They are necessary during the early amplification phase while employing host DNA replication machinery; however, E1 may not be required after 50–100 episomal copies per cell have been reached.^318^, ^319^ E1 functions as a helicase at the replication origin, and E2 stabilizes this interaction.^320^ In the latent stage, HPV replicates once per cell cycle. In the squamous epithelium, the late stage of HPV replication and vegetative viral DNA replication occurs following keratinocyte differentiation.^321^ Expression of viral E5, E6, and E7 proteins drive keratinocyte into the S phase which enables access to host factors,^322^ E5 also has a role in the formation of koilocyte.^323^ L1 and L2 expression, which allows genome packaging is required for completing of HPV replication cycle.^324^ Viral particles mature at the upper layers of terminally differentiated squamous epithelia. Disulfide interactions that develop between L1 proteins during maturation promote the capsid's stability and resistance to proteolytic digestion.^325^ In addition, E4 may have a role in viral release.^326^

#### 6.2.1 Role of miRNAs in HPV infection

HPVs utilize cellular miRNAs to modulate their differentiation‐dependent replication cycle. HPV infection leads to decreased or increased expression of miRNAs. hsa‐miR‐145 is one of the potentially HPV‐targeting miRNAs. HPV infection downregulates the expression of hsa‐miR‐145. The seed sequences of hsa‐miR‐145 were observed in E1 and E2 coding regions of HPV‐31 and also most HPV types. E1 is greatly responsive to suppression by hsa‐miR‐145. Observation of the roles of E6 and E7 oncoproteins showed the role of E7 in suppressing hsa‐miR‐145. Stable viral copy numbers were significantly reduced by the heterologous expression of hsa‐miR‐145. Also, differentiation‐dependent amplification of the viral genome was blocked by hsa‐miR‐145 heterologous expression. Downregulation of hsa‐miR‐145 in HPV infection allows continuous viral infections and facilitates amplification of proteins and viral genome.^327^ In another study, it was demonstrated that following differentiation, viral E7 protein blocks the upregulation of hsa‐miR‐203 expression. In the human keratinocytes, p63 3′‐UTR is being targeted by hsa‐miR‐203, which leads to negative regulation of p63 levels. P63 protein has the function of modulating the balance between epithelial proliferation and differentiation. Blocking of hsa‐miR‐203 by E7 leads to increasing levels of p63 which helps to keep cells active and promotes cellular proliferation. Furthermore, MAPK/PKC pathway signaling activates hsa‐miR‐203 and affects its levels. The MAPK/PKC pathway‐dependent activation of hsa‐miR‐203 expression is also blocked by viral E7. Overexpression of hsa‐miR‐203 interferes with HPV amplification. Taken together, in differentiating cells, viral proteins block the expression of hsa‐miR‐203 to have productive replication.^328^

As mentioned earlier, cervical cancer is closely related to HPV infection. In a study, Liu demonstrated that in HPV16‐positive cervical cancer cells, HPV16 E6 leads to overexpression of hsa‐miR‐20a. This overexpression promoted growth in HPV16‐negative C33A cells, and the knockdown of this miRNA in HPV‐positive CaSki cells showed a growth‐inhibitory effect. TargetScan predicted PDCD6 as a target gene of hsa‐miR‐20a. However, PDCD6 expression was negatively regulated by hsa‐miR‐20a. Furthermore, it was demonstrated that activation of AKT and p38 might be related to hsa‐miR‐20a growth‐promoting effect. This study demonstrated HPV16 E6‐related regulation of hsa‐miR‐20a expression and oncogenic role of hsa‐miR‐20a in cervical cancer.^329^

It was reported that ectopic overexpression of HPV16 E6 in HPV16 E6 expressing HaCaT and HEK293T cells resulted in the downregulation of hsa‐miR‐2861. hsa‐miR‐2861, as a tumor suppressor, prevented invasion and cell growth of cervical cancer and its level showed a reduction in lymph node metastasis and advanced tumor stage. In cervical cancer cells, EGFR, AKT2, and CCND1 expression can directly be targeted by hsa‐miR‐2861, which is involved in the tumor suppression function of this miRNA. The invasion and proliferation of cervical cancer cells were suppressed by overexpression of hsa‐miR‐2861. The study indicated that utilizing this pathway and hsa‐miR‐2861 may control cervical cancer initiation and progression induced by HPV16 E6.^330^

PPARγ belongs to the nuclear receptor superfamily, regulates gene expression, and has a transcription factor role. Also, it can promote differentiation, apoptosis, and inhibit growth which makes it a tumor suppressor. PPARγ is involved in various diseases, including cancer.^331^, ^332^ In comparison with normal tissues, cervical cancer tissues have lower PPARγ levels.^333^ In a study performed by Zhang et al., it was demonstrated that HPV16 E7 upregulates hsa‐miR‐27b. PPARγ, as a target of hsa‐miR‐27b, can downregulate the sodium‐hydrogen exchanger isoform 1 (NHE1) which leads to suppression of cervical cancer. Inhibition of hsa‐miR‐27b leads to decreased ability of HPV16 E7 in activating NHE1 expression or suppressing PPARγ. In HPV16‐positive cervical cancer tissues, overexpression of hsa‐miR‐27b increases NHE1 expression and lowers the expression of PPARγ which increases invasion and proliferation of cervical carcinoma cells.^334^

Zhao et al. identified the downregulation of hsa‐miR‐154‐5p during cervical cancer progression. They transfected SiHa, HPV16‐positive cervical cancer cell line, with hsa‐miR‐154‐5p inhibitor and mimic. Increased hsa‐miR‐154‐5p expression resulted in reduced invasion, migration, and proliferation in SiHa cells while an inverse effect was observed in utilizing of hsa‐miR‐154‐5p inhibitor. Furthermore, the silencing of CUL2, a target of hsa‐miR‐154‐5p, reduced invasion, migration, proliferation, and increased pRb expression. In HPV16 E7‐induced cervical carcinogenesis, targeting CUL2 by hsa‐miR‐154‐5p regulates pRb expression, introducing it as a tumor‐suppressive factor.^335^

Tissue and organ homeostasis can be controlled by the Hippo signaling pathway through modulation of apoptosis and cellular proliferation.^336^ LATS1, LATS2, STK3, STK4, MOBs, and SAV1 comprise the Hippo kinase cascade.^337^ Yes‐associated protein (YAP) is a target of Hippo, and serine phosphorylation of YAP occurs following activation of these kinases.^338^ In cervical cancer, the expression levels of YAP have been increased. In a study, decreased levels of the master Hippo regulatory kinase STK4 were identified in cervical cancer cell lines and samples. The HPV E6 and E7 upregulate oncomiR hsa‐miR‐18a expression by targeting 3′‐UTR of STK4 mRNA which mediates the downregulation of STK4 expression. The knockdown of hsa‐miR‐18a resulted in the activation of the Hippo pathway and increased expression of STK4.^339^

#### 6.2.2 Viral miRNAs

In 2011, Gu et al. predicted HPV‐encoded miRNAs. They predicted that HPV‐38 encodes miRNA conserved to human hsa‐miR‐let‐7a and mucosal HPV types express miRNAs related to hsa‐miR‐466, ‐467, and ‐669.^340^ Establishing of miRNA libraries from HPV cell lines and HPV‐related cervical cancer resulted in identification of two HPV16‐, one HPV68‐, and one HPV38‐encoded miRNAs. Furthermore, two putative viral target sites were identified for the two HPV16 miRNAs.^341^ To find more miRNAs involved in HPV infection, refer to Table 6.

**Table 6:** miRNAs and their contribution to HPV infection and related cancers.

miRNA	Cell line	Deregulation	Target/human or viral	Effect	References
hsa‐miR‐221	HPV16‐positive SiHa, HPV18‐positive HeLa cell lines	Upregulated	SOCS1/Human	Overexpression inhibits in vitro HPV16 E1‐E2 mediated DNA replication, promotes the expression of ISGs genes, upregulates protein and mRNA levels of IFN‐α and IFN‐β, downregulates SOCS1.	342
hsa‐miR‐4454‐5p	HPV18‐positive HeLa, HPV16‐ positive CaSki cell	Upregulated	ABHD2, NUDT21/Human	Inhibits the apoptosis, promote the proliferation, invasion and migration, downregulates ABHD2 and NUDT21. Upregulation enhances cervical cancer cell invasion and migration.	343
hsa‐miR‐224‐3p	Siha, Hela cell lines	Upregulated	FIP200 gene/Human	Directly targets FIP200 expression and inhibits autophagy in HPV‐infected and hrHPV‐induced cervical cancer cells.	344
hsa‐miR‐93‐5p	HPV16‐positive CaSki, HeLa cell lines. HPV18‐positive HeLa, C4‐1 cell lines	Upregulated	BTG3/Human	HR‐HPV infection upregulates hsa‐miR‐93‐5p expression and downregulates BTG3, hsa‐miR‐93‐5p might have a oncogenic role and function in progression of cervical cancer.	345
hsa‐miR‐182	HeLa, SiHa cell lines	Upregulated	‐	E2F releases by binding of HR HPV E7 to pRb and leads to TGF‐β overexpression. hsa‐miR‐182 expression upregulates by HR HPV E7 through TGF‐β/Smad4 signaling pathway.	346
hsa‐miR‐21	The K14E7 HPV16 transgenic mice, HPV16 E7 expressing cell line	Upregulated	PTEN/Human	hsa‐miR‐21 expression increases in the presence of HPV16 E7 and 17β‐estradiol (E2), upregulation inhibits PTEN expression. Also confirmed in HPV16 containing cervical cancer samples.	347
hsa‐miR‐15a	Hypopharyngeal squamous cell carcinoma and FaDu cells	Upregulated	BCL2L2, BCL2/Human	In HPV‐16 E6‐E7 expressing FaDu cells and HPV‐16‐positive patients showed significantly increased levels of hsa‐miR‐15a. hsa‐miR‐15a inhibitor in HPV‐positive HSCC reduced apoptosis. Negatively regulates BCL2 and BCL2L2 posttranscriptional expression and induces apoptosis in HPV‐positive HSCC.	348, 349
hsa‐miR‐27b	HPV16‐positive CaSki and SiHa cell lines	Upregulated	PLK2/Human	DGCR8 increased by HPV16 E7 upregulates hsa‐miR‐27bhsa‐miR‐27b downregulates PLK2, upregulation of miRNA increase cell proliferation and miRNA plays oncogenic role.	350
hsa‐miR‑130a‑3p	HeLa and SiHa cervical cancer cell lines	Upregulated	ERα and AR receptors/Human	hsa‐miR‐130a‐3p directly targets ERα and AR, upregulation contributes to tumor progression, inhibition of miR‑130a‑3p, and overexpression of AR and ERα inhibits cervical cancer invasion and proliferation.	351
hsa‐miR‐155‐5p	HPV16‐positive Siha and HPV18‐ positive HeLa cells	Downregulated	3′ UTR of PDK1/Human	Downregulation of miRNA inhibits autophagy, overexpression of the miRNA inhibits PDK1 expression and enhances autophagy, downregulation of miRNA promotes PDK1 and AKT/mTOR signaling.	352
hsa‐miR‐199a	HPV‐positive CC tissue	Downregulated	Wnt5a/Human	miRNA inactivate the Wnt signaling pathway by targeting Wnt5a, HPV induces HDAC6 expression which downregulates hsa‐miR‐199a and leads to upregulation of the Wnt5a, and progression of HPV‐positive CC.	353
hsa‐miR‐218	HPV16‐positive SiHa, CaSki cervical cell lines, and HPV18‐positive HeLa cell line	Downregulated	LAMB3/Human	The E6 oncogenic protein of HR HPV‐16 reduced hsa‐miR‐218 expression and in the transcriptional level increases LAMB3 expression, may promote viral infection and tumorigenesis.	354
hsa‐miR‐143	The K14E7 HPV16 transgenic mice, HPV16 E7 expressing cell line	Downregulated	BCL‐2/Human	hsa‐miR‐143 expression decreased in the presence of HPV16 E7 andE2, the expression of BCL‐2 increased. Also confirmed in HPV16‐containing cervical cancer samples.	347
hsa‐miR‐218	HPV16 overexpressed Hela cells, U2OS cells	Downregulated	PTEN/Human	HPV type 16 E6 protein downregulated hsa‐miR‐218 expression. Overexpression of hsa‐miR‐218 could target PTEN and promote U2OS and Hela cells proliferation, overexpression of E6 promote invasion and migration of Hela cells.	355
hsa‐miR‐1246	HPV16 E6 positive C33A cell lines	Downregulated	DYRK1A, Human	HPV16 E6 infection decreases hsa‐miR‐1246 expression and increases DYRK1A protein expression. Deregulation of hsa‐miR‐1246 is involved in cervical cancer progression.	356
hsa‐miR‐641	Cervical cancer tissues and cell lines	Downregulated	3′ UTR of ZEB1/Human	hsa‐miR‐641 decreases ZEB1 expression in cervical cancer tissue, ZEB1 upregulates in downregulation of miRNA and reduces tumor suppressive function of hsa‐miR‐641. Upregulated hsa‐miR‐641 has tumor suppressive roles	357
hsa‐miR‐122	SiHa cell lines	Mediated expression (one fifth) compared to Huh7 cells	HPV16 E6 mRNA/Virus SOCS1/Human	Inhibits HPV16 E6 expression through directly binds to its mRNA. Induce IFN‐Is production through pairing with SOCS1, promotion of IFN signaling pathway inhibits the expression of HPV16.	358
hsa‐miR‐129‐5p	Hela cells	Downregulated	SP1/Human	Increasing of HPV‐18 E6/E7 and development of cervical intraepithelial lesion lowers the expression of hsa‐miR‐129‐5p. Overexpression of hsa‐miR‐129‐5p inhibits growth, proliferation, and blocks G1 cell cycle in Hela cells, reduces SP1 protein levels and downregulates HPV‐18 E6 and E7 expression.	359
hsa‐miR‐22	Cervical cancer tissues and cell lines	Downregulated	3′ UTR of HDAC6/Human	hsa‐miR‐22 downregulates HDAC6 expression at posttranscriptional level which is regulated by E6/p53 pathway. Cell proliferation and migration inhibited by ectopic expression of hsa‐miR‐22 and apoptosis induced in cervical cancer cell lines.	360
hsa‐miR‐3156‐3p	HPV16/18 positive CC lesions, HeLa, CaSki, SiHa cell lines	Downregulated	SLC6A6/Human	Modulates invasion, migration, apoptosis, and proliferation of cervical cancer cells, negatively downregulates SLC6A6, downregulation of miRNA increase SLC6A6 expression, miRNA inhibits cervical cancer tumorigenesis, downregulation promotes cervical cancer pathogenesis.	361
hsa‐miR‐409‐3p	Dysplastic cervical tissue, precancerous cervical lesion, CaSki, SiHa, C‐41, and HeLa cell lines	Downregulated	Viral E6/Virus	miRNA has lower expression in cervical dysplastic tissue. HPV16/18 E6 mRNA levels has inverse relation with miRNA level and miRNA directly reduces E6 mRNA levels. Upregulation of miRNA reduces migration and proliferation of cervical cancer cells.	362

### 6.3 Herpes simplex virus

Herpesviruses as the *Herpesviridae*, are ds DNA viruses with lytic and latent infection and the ability to cause lifelong infection. Based on their sequence homology, *Alphaherpesvirinae*, *Betaherpesvirinae*, and *Gammaherpesvirinae* consist of three subfamilies of herpesviruses.^363^ Human herpes simplex viruses (HSV) type 1 and 2 (HSV‐1, HSV‐2) belong to the *Alphaherpesvirinae* subfamily and cause oral and genital blisters, respectively.^364^ The transmission of HSV‐1 occurs via contact with the virus present in saliva or skin, leading to the manifestation of symptoms primarily in or around the mouth. On the other hand, HSV‐2 is primarily disseminated through sexual contact, leading to symptoms mainly in the genital regions.^365^ An estimated 3.7 billion people are struggling with HSV‐1. HSV‐2, with an estimated infection of 491 million people, is the main reason for recurrent genital herpes.^366^ However, a growing percentage of anogenital herpetic infections are associated with HSV‐1 infection.^367^ Infection with HSV‐1 virus can lead to various life‐threatening diseases. Among the clinical manifestations, it can be blepharitis, conjunctivitis, uveitis, and keratitis; relapsing viral keratitis leads to severe blindness. It is worth mentioning that the corneal tissue is a site for latent HSV‐1 infection.^368^ Also, HSV‐1 develops latency in peripheral ganglia sensory neurons, and variety of stresses can lead to recurrent virus reactivation. Reactivated viral particles can cause herpes simplex encephalitis which is rare but severe form of diffuse acute infection.^366^

HSV has a 125 kbp dsDNA with two unique large and short regions (UL and US). Inverted repeat sequences flank these regions. Three origins of replication, including two OriS and OriL are present in the HSV genome. OriL is located between the genes encoding catalytic subunit of the polymerase and UL29 genes of HSV, which encode ICP8 replication proteins. ICP8 proteins as the major ssDNA‐binding proteins (SSB) function in the regulation of viral gene expression, formation of prereplicative sites, and viral DNA synthesis. OriS is located between the genes encoding ICP4 and either ICP22 or ICP47 immediate‐early proteins.^369^

**Figure 14:**
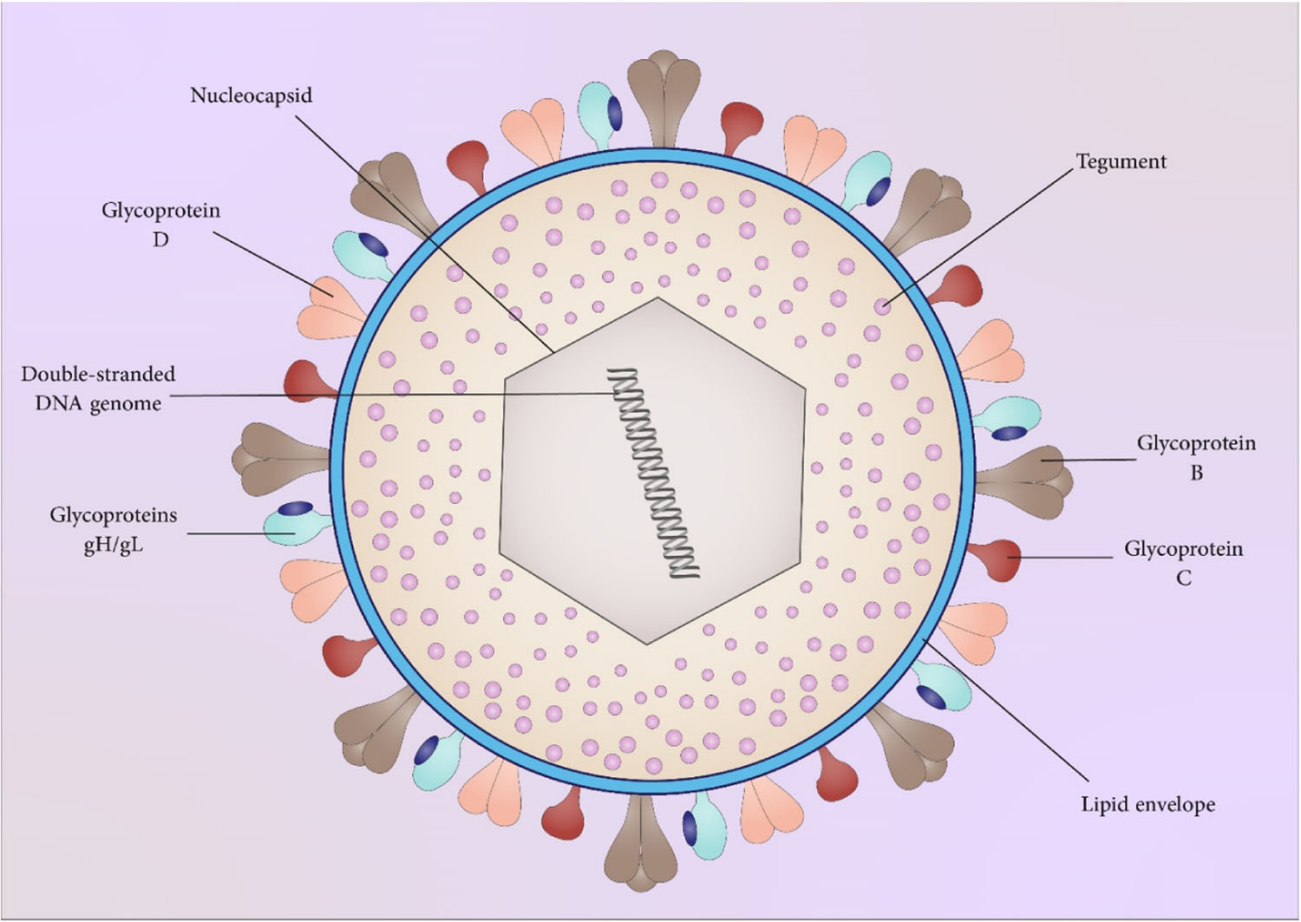
Schematic representation of HSV structure. [Color figure can be viewed at wileyonlinelibrary.com]

The HSV has a spherical shape and its icosahedral capsid has a diameter of 125 nm. HSV is comprised of a lipid envelope and its nucleocapsid is surrounded by a protein‐rich tegument layer.^370^, ^371^ Three major mature capsid forms of A, B, and C are detected in HSV. While capsid C contains viral DNA and matures into an infectious virion, capsids A and B are empty and scaffold‐containing capsids, respectively.^372^ Based on the studies, at least five glycoproteins of virus envelope, including gB, gC, gD, gH, and gL play a role in HSV entry (Figure 14).^373^, ^374^ gD as the main receptor‐binding protein of HSV binds to nectin‐1, nectin‐2, herpes virus entry mediator (HVEM) in HSV‐1 and HSV‐2, and also 3‐O‐sulfated heparin sulfate (3‐OST HS) for HSV‐1.^375^ Two entry pathways have been proposed for HSV‐1. One of the critical mechanisms is the fusion of the viral envelope with the plasma membrane and transfer of the viral content into the cytoplasm. The important step in this process is the binding of surface glycoproteins of the virus with the surface receptors of the target cells.^376^ The binding of gD to cellular receptors results in an interaction between gH–gL heterodimer and gD. Nectin‐1, Herpes Virus Entry Mediator (HVEM), and 3‐O‐sulfonated heparan sulfate are host cell receptors for HSV gD. gH‐gL transmits a signal to gB and leads to a conformational change and insertion of hydrophobic fusion loops into the membrane.^375^ Furthermore, binding of gB to cell surface paired immunoglobulin‐like type 2 receptor (PILR) is required for HSV entry.^377^ The second mechanism is mediated through the endocytosis‐dependent pathway. After binding the virus to the cell receptors, the virus enters the target cell and the virus envelope is integrated with intracellular vesicles. It is worth mentioning that viral gC and gB glycoproteins interact with cell surface glycosaminoglycans (GAG), especially heparan sulfate.^378^ In the cytoplasm of the host cell, some capsid‐related tegument proteins stay in the cytoplasm while other proteins including VP16 migrate to the nucleus to promote the transcription of viral genes.^379^

For the entry of viral DNA into the cell nucleus, the viral capsid docks to nuclear pore complexes. Studies identified Nup214, Nup358 nucleoporins, and VP1/2 as factors associated with the binding of capsid to the host nucleus.^380^ Host RNA polymerase II synthesizes the viral mRNA with the participation of viral factors. HSV‐1 gene expression is categorized into three groups including α (immediate–early or IE), β (early), and γ (late). IE genes as the most complex genes are the first transcribed genes, which are stimulated by viral VP16 tegument protein and transcribed by host factors. The transcription of early genes occurs after synthesis of IE proteins and the transcription of late genes only happens after the starting of viral DNA replication. HSV‐1 genome encodes five IE proteins, among them ICP4 and ICP27 are two examples of IE proteins which are required for productive infection. ICP4 is a functional protein in transcription of early and late genes and ICP27 enhances processing and export of viral mRNAs. ICP0, as another IE protein, is not essential in HSV‐1 replication. However, it is required in proceeding to lytic replication. DNA replication of the HSV initiates after the start of expression of early genes.^381^ HSV‐infected cells facilitate the infection by producing viral capsids; genome‐lacked noninfectious light particles and infective virions which leads to bringing additional tegument proteins to the host cells.^382^ Capsid assembly and DNA packaging are conducted in the nucleus. After some complicated steps, tegument and envelope are completed and mature virus particles are released from the cell.^383^ Successful HSV replication leads to alteration in the structure of host cells and induces cell death. The entrance to the latent phase happens 10–14 days after the initiation of the viral replication.^384^ In the latent phase, only the latency‐associated transcript (LAT) is expressed. LAT acts as a miRNA precursor and encodes viral miRNAs.^385^, ^386^ HSV‐1 and HSV‐2 encode 27 and 24 functional viral miRNAs, respectively.^387^, ^388^

#### 6.3.1 Role of miRNAs in HSV infection

hsa‐miR‐155‐5p, as one of the most studied miRNAs, is demonstrated to regulate autophagy genes and mediate T lymphocyte differentiation. It has a promotor of proliferation role in different cancer cells.^389^, ^390^, ^391^, ^392^ It was reported that the expression of hsa‐miR‐155‐5p was upregulated by HSV‐1 infection. This upregulation resulted in increased HSV‐1 replication and spread. Also, SRSF2 transcription is epigenetically regulated and enhanced by miR‐155‐5p through modifying histone modifications near the transcription start site (TSS). The transcriptional activator SRSF2 plays an important role in viral gene expression. These changes along with the upregulation of hsa‐miR‐155‐5p increase HSV‐1 gene expression and replication.^393^

Xie et al. demonstrated that the expression of the hsa‐miR‐373 was upregulated by HSV‐1 infection. *In vitro* knock‐down and overexpression experiments showed the role of hsa‐miR‐373 in promoting HSV‐1 replication. Also, they indicated that IRF1, which regulates type I IFN expression, is a direct target of the miRNA. hsa‐miR‐373 targets 3′‐UTR of IRF1 which leads to the promotion of HSV‐1 replication Furthermore, it was shown that in HSV‐1‐infected cells, hsa‐miR‐373 suppressed the expression of type I IFN and interferon‐stimulated gene (ISG). These actions enhance HSV‐1 infection in HeLa cells.^394^

HSV‐1 infection decreases the expression of hsa‐miR‐649. This miRNA promotes the infection of HSV‐1. Downregulation of this miRNA reduces viral DNA levels, while the overexpression has an inverse effect and facilitates HSV‐1 replication. 3′‐UTR of MALT1 (mucosa‐associated lymphoid tissue lymphoma translocation gene 1) as a novel direct target of this miRNA which is negatively regulated by hsa‐miR‐649. MALT1 functions as an antiviral host factor. Overexpression of hsa‐miR‐649 inhibits endogenous levels of MALT1 which promotes HSV‐1 replication. However, increasing MALT1 levels inhibited viral infection.^395^

Wang and coworkers demonstrated that overexpression of hsa‐miR‐101 could suppress HSV‐1 infection. They introduced 3′‐UTR of mitochondrial ATP synthase subunit beta (ATP5B) as a direct target of hsa‐miR‐101. ATP5B showed a pro‐viral role, hence its knockdown inhibited HSV‐1 infection. ATP5B suppression via hsa‐miR‐101 prevented HSV‐1 replication.^396^ Another research finding indicated that HSV‐1 induces the expression of hsa‐mir‐101‐2. They demonstrated that ICP4, an essential regulatory factor of activating transcription of early and late viral genes, activated the expression of hsa‐miR‐101 by directly binding to its precursor hsa‐miR‐101‐2. Also, they identified RNA‐binding protein G‐rich sequence factor 1 (GRSF1) as the direct target of hsa‐miR‐101. This factor facilitates viral proliferation through direct binding to HSV‐1 p40 mRNA and enhancing its expression. ICP4‐induced expression of hsa‐miR‐101 reduces HSV‐1 replication by downregulating GRSF1.^397^

The levels of hsa‐miR‐138 which is enriched in various neuronal tissues and cells remain high after HSV‐1 infection. This miRNA showed potential targets in 3′‐UTR of ICP0 mRNA in both HSV1 and HSV‐2. Transfection of hsa‐miR‐138 mimic resulted in reduced mRNA expression and ICP0 protein and decreased the expression of ICP0 in both HSV‐1‐infected and ‐transfected cells. In a seed region‐dependent manner, this miRNA can inhibit ICP0 expression. Also the lytic viral gene expression was downregulated by hsa‐miR‐138 in HSV‐1‐infected cells. The mutation of hsa‐miR‐138 target site in ICP0 led to increased mortality and morbidity in infected mice. This miRNA prevents the death of host cells which results in the promotion of HSV‐1 latency, by targeting the expression of ICP0.^40^

hsa‐miR‐23a level is not steadily decreased or increased during infection. However, HSV‐1 infection induces hsa‐miR‐23a expression, and its highest expression was reported 18 h postinfection. IRF1 protein with a role in proapoptotic signaling, inflammation, and innate antiviral immunity was introduced as a target of hsa‐miR‐23a. IRF‐1 promotes the expression of RSAD2 which can inhibit viral replication. hsa‐miR‐23a‐induced downregulation of IRF‐1 leads to suppression of RSAD2 which results in increased HSV‐1 replication. Accordingly, miR‐23a facilitates HSV‐1 replication and increases the number of infected cells.^398^

In HSV1‐infected cells, transcription factor AP1 induces the expression of the hsa‐miR‐24 through HSV1‐induced activation of the MAPKs which leads to promoting of virus replication. This miRNA inhibits translation of the STING mRNA by binding to its 3′ UTR. Inhibiting the expression of the hsa‐miR‐24 suppressed virus replication in infected cells. Furthermore, morbidity and death of the infected mice reduced by the injection of antihsa‐miR‐24 indicates the possibility of utilizing hsa‐miR‐24 against HSV1 infection.^399^

#### 6.3.2 Viral miRNAs

Both HSV‐1 and HSV‐2 encode 18 stem–loops, which results in 27 and 24 miRNAs, respectively. In 2006, Cui et al. reported the first HSV‐1 miRNA, miR‐H1 which is expressed as a late gene.^400^ Following this finding, several studies were conducted to reveal more HSV miRNAs. Most of the abundantly expressed HSV‐1 miRNAs are generated from the LAT locus.^401^ For instance, it was demonstrated that miR‐H4‐3p and miR‐H3 target viral ICP34.5 expressions through an antisense mechanism. Also, viral miR‐H2‐3p can reduce the protein levels of viral ICP0 and viral miR‐H6 reduces ICP4 protein expression and may therefore increase latent state durability.^385^ Furthermore, this viral miRNA decreases the interleukin 6 production. Transfection of miR‐H6 mimics to human cornea epithelial (HCE) cells infected with HSV‐1, resulted in inhibition of viral ICP4 expression as well as HSV‐1 replication and productive infection.^368^

Despite other viral miRNAs, miR‐H27 as another HSV‐1 miRNA, has an important function in viral proliferation, and replication. This viral miRNA targets and blocks cellular transcriptional repressor Kelch‐like 24 (KLHL24). This cellular factor inhibits transcriptional efficiency of viral IE and E genes.^402^

In a recent search conducted on gingival biopsies derived from subjects with periodontitis, overexpression of miR‐H1 was observed. Overexpression of miR‐H1 in human oral keratinocytes (HOK) implied the role of this v‐miRNA in the regulation of viral entry and infection.^403^ Also, it was established that miR‐H1 targets the endocytosis pathway through regulating and targeting of SORT1. Upon viral infection, SORT1 stimulates pro‐inflammatory immune response. As a result, miR‐H1 downregulates Sort1, promoting cell infection and evasion of the host immune responses.^404^

**Figure 15:**
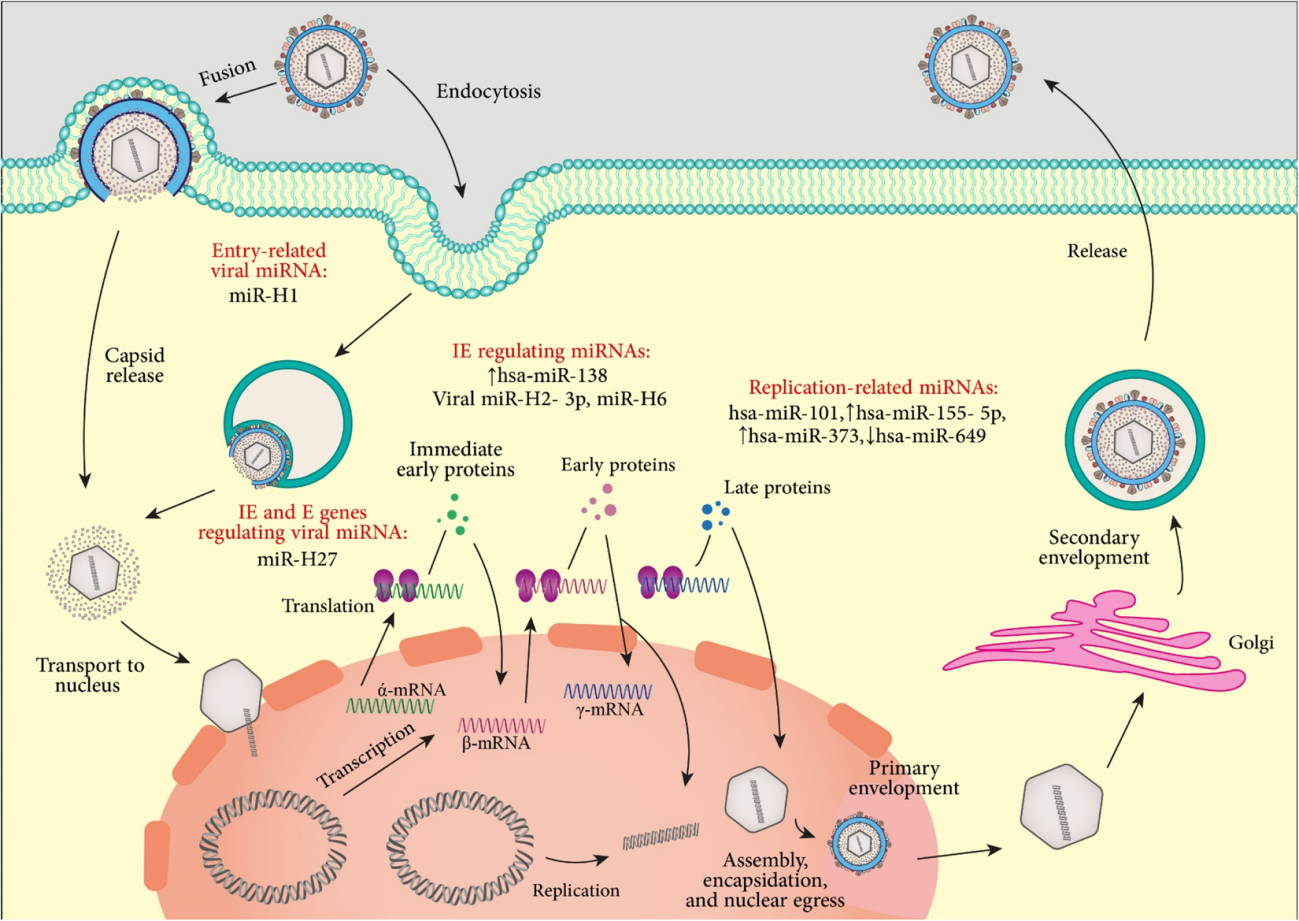
Summary of cellular and viral miRNAs involved in HSV infection. IE genes encode five proteins including ICP0 and ICP4. Viral miR‐H2‐3p and hsa‐miR‐138 target ICP0 while viral miR‐H6 targets ICP4. Viral miR‐H27 inhibits the transcriptional efficiency of IE and E genes. Host encoded hsa‐miR‐101, hsa‐miR‐155‐5p, hsa‐miR‐373, and hsa‐miR‐649 regulate viral replication. Also, viral miR‐H1 regulates viral entry and infection. miRNA dysregulation is indicated by an up or down arrow. [Color figure can be viewed at wileyonlinelibrary.com]

Microarrays were used to screen miRNA expression in 10 patients with primary lung cancer without known metastases and 10 patients with lung cancer bone metastases. Results indicated that the expression of miR‐H9‐5p encoded by HSV‐2 LAT sequence was highly increased in bone metastasis of lung cancer patients. This viral miRNA binds to 3′‐UTR of SOCS2 and regulates its expression. Similar to phenotypic effects produced by Hsv2‐miR‐H9‐5p mimic, the knockdown of SOCS2 also resulted in antiapoptotic cell behavior, as well as increased migration and metastasis. In vitro study indicated that overexpression of miRNA using Hsv2‐miR‐H9‐5P mimic greatly improved the cell migration of either LTEP‐α‐2 or SPC‐α‐1 cell lines.^405^ Similar to HSV‐1, miR‐H2 targets ICP0 and ICP34.5 of HSV‐2.^406^ Figure 15 summarizes important cellular and viral miRNAs involved in HSV infection.

### 6.4 Epstein–Barr virus

Epstein–Barr virus (EBV) as a DNA virus is a member of the *Herpesviridae* family and belongs to the *Gammaherpesvirinae* subfamily. EBV as a ubiquitous virus is also termed as human herpes virus 4 (HHV4). Infection with the EBV virus was first discovered in endemic Burkitt's lymphoma cells. This virus is related to several diseases which their incidence varies in different places, and it is reported that more than 90% of the world's population is infected with this virus. This virus transmits through saliva, sexual contact, blood transfusion, and organ transplant.^407^, ^408^, ^409^ EBV infects B cells and epithelial cells and it can lead to other malignancies, including Hodgkin, Burkitt, T‐cells lymphomas, nasopharyngeal and gastric carcinomas, and lupus erythematosus as an autoimmune disease.^410^ Symptomatic infections are most common in adolescents and adults. Infections with EBV in children usually cause no symptoms, or the symptoms are similar to those caused by other mild, brief illnesses in children.^411^, ^412^

**Figure 16:**
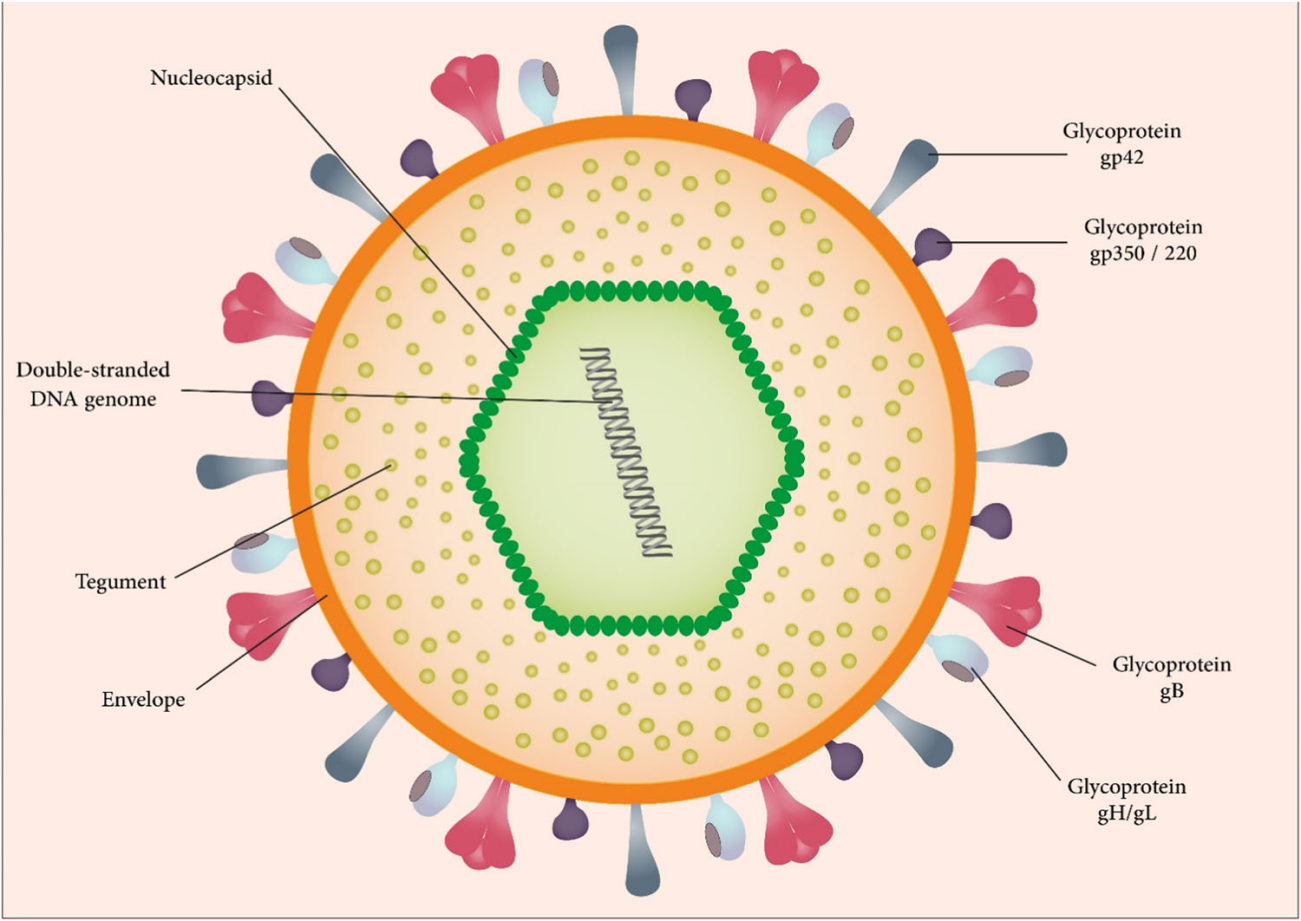
Schematic representation of EBV structure. [Color figure can be viewed at wileyonlinelibrary.com]

EBV contains a 170 kb ds DNA that encodes more than 80 genes.^413^ The structure of the virion is similar to other herpesviruses and has a common icosahedral capsid of 162 capsomers. Viral capsid antigen (VCA) is the major capsid protein while BDLF1 and BORF1 are predicted to be minor capsid proteins.^414^ Similar to other herpesviruses, EBV contains a tegument space between the outer membrane and nucleocapsid. BSRF1 and BSRF2 are two examples of EBV tegument proteins which forms a hetero‐complex (Figure 16).^415^

Similar to other herpesviruses, EBV also has latent and lytic phases. An immune escape strategy used by herpesviruses is mediated through expressing no viral genes and remaining in the latent phase as an episome.^416^ EBER (EBV‐encoded small RNA), nontranscribed BART RNAs, two LMPs (latent membrane proteins), and five EBNAs (EBV‐encoded nuclear antigens) are being expressed by EBV latent genome. EBV was classified by alleles at the EBNA2 and EBNA3 into type 1 and type 2. EBV type 1 is more prevalent around the world.^417^ The entrance mechanism of the virus is similar to HSV. However, in the B cell entrance of the EBV, gp42 is the receptor‐binding protein instead of gD.^375^ Five glycoproteins, including gB, gH, gL, gp42, and gp350/220 are involved in the B cells entry of the virus.^418^ Initially, human oropharynx epithelial cells are the first target cells in EBV infection. In the next step, EBV, after replication in oropharyngeal epithelial cells, spreads to B cells and may remain latent in B cells. The latent phase of the EBV infection has three latent programs: latency types III, II, and I. Different viral genes are being expressed in each latency program in various EBV malignancies. Latency type III is the main characteristic of lymphoblastoid lymphomas and active lymphoproliferative diseases, Hodgkin's disease and NPC show type II latency, and Burkitt's lymphoma shows latency I.^419^ The spectrum of expressed viral genes in each latency type is shown in Table 7. In addition to viral genes, latent III profile consists of 25 pre‐miRNAs. More than 40 mature viral miRNAs are derived from 25 precursors.^420^ EBV miRNAs are derived from two clusters: BHRF and BART. Except for BHRF1‐1, ‐2, and ‐3 which are encoded from the BHRF cluster, other miRNAs are derived from the BART cluster.^421^ Zta (also known as ZEBRA and BZLF1) and Rta (also known as BRLF1) as two viral transcription factors trigger the transition to lytic state and productive cycle.^422^ At least six viral proteins in addition to Zta are required for viral DNA synthesis. The viral DNA replication is independent of cellular DNA replication. OriLyt L and OriLyt R are two origins of DNA synthesis. These regions recruit viral replication proteins, including primase‐helicase complex, BMRF1 (DNA polymerase processivity factor), BALF2 (ssDNA–binding protein), and BALF5 (DNA polymerase). Amplified viral DNA concatemers via rolling cycle model pack into viral capsids.^423^ The next step is expression of viral structure genes. EBV encodes approximately 36 late genes responsible for coding tegument proteins, glycoproteins, and viral structural proteins.^424^ The last step is the practical assembly of EBV. BDLF1 and BORF1 minor capsid proteins form a triplex. This triplex in addition to BFRF3 minor protein, BcLF1 major protein, BdRF1 scaffold protein, and BVRF2 protease forms mature nucleocapsid resembling icosahedron.^425^

**Table 7:** Latency gene expression in the different types of EBV latency.

Latency type	Expressed viral genes
Type I	EBER1, EBER2, EBNA‐1, LMP‐2A/B, BART miRNAs
Type II	EBER1, EBER2, EBNA‐1, LMP‐2A/B, LMP‐1 (type IIa) or EBNA‐2 (type IIb), BART miRNAs
Type III	EBER1, EBER2, EBNA1, EBNA2, EBNA3A‐C, EBNA‐LP, LMP2A/B, LMP1, BART miRNAs

#### 6.4.1 Role of miRNAs in EBV infection

Investigation of EBV‐related lymphoma patients showed increased expression of hsa‐miR‐18a, a member of the hsa‐miR‐17‐92 cluster. It has been shown that hsa‐miR‐18a expression is directly linked to EBNA1 expression. Upregulation of EBNA1 and hsa‐miR‐18a leads to shorter overall survival of patients. Based on the study performed in the EBV‐positive lymphoma cells, hsa‐miR‐18a transfection increases the viral load and promotes cell proliferation. EBV infection leads to DNA damage. Hypoxia treatment and UV exposure in EBV‐positive lymphoma cells also increased the viral load and the expression of hsa‐miR‐18a, indicating the role of this miRNA as a DNA damage sensor and a regulator of EBV reactivation through inhibiting ATM expression after DNA damage.^426^

EBNA1 is expressed in all EBV‐related cancers such as EBV‐associated gastric cancer (EBVaGC). Expression of EBNA1 in EBVaGC downregulates hsa‐miR‐34a which in turn results in upregulation of NOX2. Upregulated NOX2 also increases ROS. Inhibition of EBNA1 upregulates hsa‐miR‐34a levels and changes NOX2 levels which finally confirms the role of hsa‐miR‐34a in regulating NOX2 expression.^427^ Also, Infection of primary B cells with EBV results in increased hsa‐miR‐34a expression as a consequence of the viral LMP1‐mediated IKKβ‐dependent activation of canonical NF‐κB signaling pathways.^428^

A study conducted by Treece et al. indicated that hsa‐miR‐196b expression was downregulated, whereas the expression of hsa‐miR‐155, hsa‐miR‐185, and hsa‐miR‐378 was elevated in EBV‐positive gastric tumors compared to EBV‐negative gastric tumors.^429^ hsa‐miR‐200 family is another downregulated miRNA in EBVaGC. Expression of cellular ZEB1 and ZEB2 increases as a result of decreased miRNA expression. Also, E‐cadherin expression reduces and which in turn causes loss of cell adhesion. Latent genes, including BARF0, EBNA1, and LMP2A modulate downregulation of miRNA and upregulation of ZEB1/ZEB2 which leads to decreased expression of E‐cadherin repressors and loss of cell‐to‐cell adhesion. These processes which play an important role in tumor progression.^430^ In EBV‐positive epithelial and B‐lymphocytic cell lines, the levels of hsa‐miR‐200a and 200b were negatively correlated with the levels of ZEB1/ZEB2. By downregulating ZEB1 and ZEB2, miR‐200b induces EBV‐positive cells to lytic replication and produce infectious viruses. ZEB1 and ZEB2 inhibit expression of the BZLF1 gene which plays an important role in the lytic activation of EBV. Also, this miRNA represented a positive relation with the levels of BZLF1. Taken together, hsa‐miR‐200 family has an important role in switching to the lytic phase in EBV infection.^431^

Viral LMP1 dysregulated the expression of hsa‐miR‐146a. Comparing levels of hsa‐miR‐146a in three viral latency programs revealed that the expression of this miRNA was upregulated in latency type III which expresses LMP1. LMP1‐induces hsa‐miR‐146a expression by an NF‐κB‐dependent mechanism through two binding sites of NF‐κB transcription factor located in hsa‐miR‐146a promoter.^432^ Also, in B lymphoma cells hsa‐miR‐21 was upregulated and hsa‐miR‐146a was downregulated by EBNA2, another latency III expressed viral protein. In EBNA2 transfectants, low miR‐146a expression is correlated with increased IRAK1 expression.^433^

#### 6.4.2 Viral miRNAs

Viral miRNAs also play some roles in EBV pathogenesis. EBV miRNAs can target viral mRNA, proteins, and cellular immune‐related genes to regulate immune responses. They can maintain EBV latency by suppressing apoptosis of infected cells. In EBV‐related cancers, viral miRNAs regulate cell growth and transformation, and contribute to immune escape.

Cancer progression involves B cell receptor (BCR) signaling. In cancers including lymphoma, mutations lead to constitutively activation of this pathway and increase proliferation of cancerous B cells.^434^ BCR signaling also plays a role in EBV reactivation. EBV miR‐BHRF1‐2‐5p and EBV miR‐BART2‐5p attenuate BCR signaling. These viral miRNAs do not actively inhibit spontaneous lytic replication. However, they are capable of responding to extracellular stimuli leading to virus reactivation. EBV miR‐BHRF1‐2 can also interact with 3′‐UTR of PAG1, RAC1, and GRB2, which have a role in BCR signaling.^420^ The results of a study in lymphoblastoid cell lines (LCL) demonstrated that EBV miR‐BHRF1‐2 can promote EBV lymphomagenesis by downregulating the expression of PRDM1. Upregulation of PRDM1 as a tumor suppressor gene in LCL cells leads to cell cycle arrest and apoptosis.^421^ In chronic lymphocytic leukemia, EBV miR‐BHRF1‐1 regulates the expression of the p53. Inhibition of this viral miRNA upregulates p53 protein expression. Furthermore, EBV miR‐BHRF1‐1 inhibition decreases cell proliferation and leads to induction of apoptosis and cell cycle arrest.^435^ Also, EBV miR‐BHRF1‐3 as another viral miRNA attenuates the lytic phase of the virus by inhibiting Zta induction of BRLF1. EBV miR‐BHRF1‐3 impacts virus production by targeting the 3′UTR of the BZLF1.^436^

EBV miR‐BART16 can also inhibit IFN signaling through direct targeting of a coactivator in IFN signaling, CREB‐binding protein. IFN‐α–induced gene transcription is impaired by CBP downregulation. The formation of latent EBV infection is facilitated by miR‐BART16‐induced suppression of type I IFN signaling which leads to enhanced viral replication.^437^

MAP3K2 plays an essential role in signaling cascades involved in initiating lytic viral replication. It was demonstrated that 3′‐UTR of MAP3K2 encoding mRNA is targeted by miR‐BART18‐5p. Therefore, overexpression of this viral miRNA downregulates the expression of MAP3K2. In latently infected memory B cells, EBV miR‐BART18‐5p reduces the risk of reactivation and maintains latency. Also, this miRNA reduces viral replication. EBV miR‐BART18‐5p is the first identified viral miRNA with the ability of inhibiting a target in the MAP kinase signaling cascade.^438^ In addition, BART5‐3p is the first identified EBV miRNA with the ability to degrade p53 protein. In some gastric cancer cells and nasopharyngeal carcinoma cells, this miRNA targets 3′‐UTR of the TP53 gene which has a tumor suppressor function. Also, this miRNA inhibits cell apoptosis through downregulation of FAS, BAX, and CDKN1A and promotes cell growth. Furthermore, it was revealed that BART5‐3p may play a role in resistance to chemotherapy drugs and facilitate staying in the latent phase.^439^

**Figure 17:**
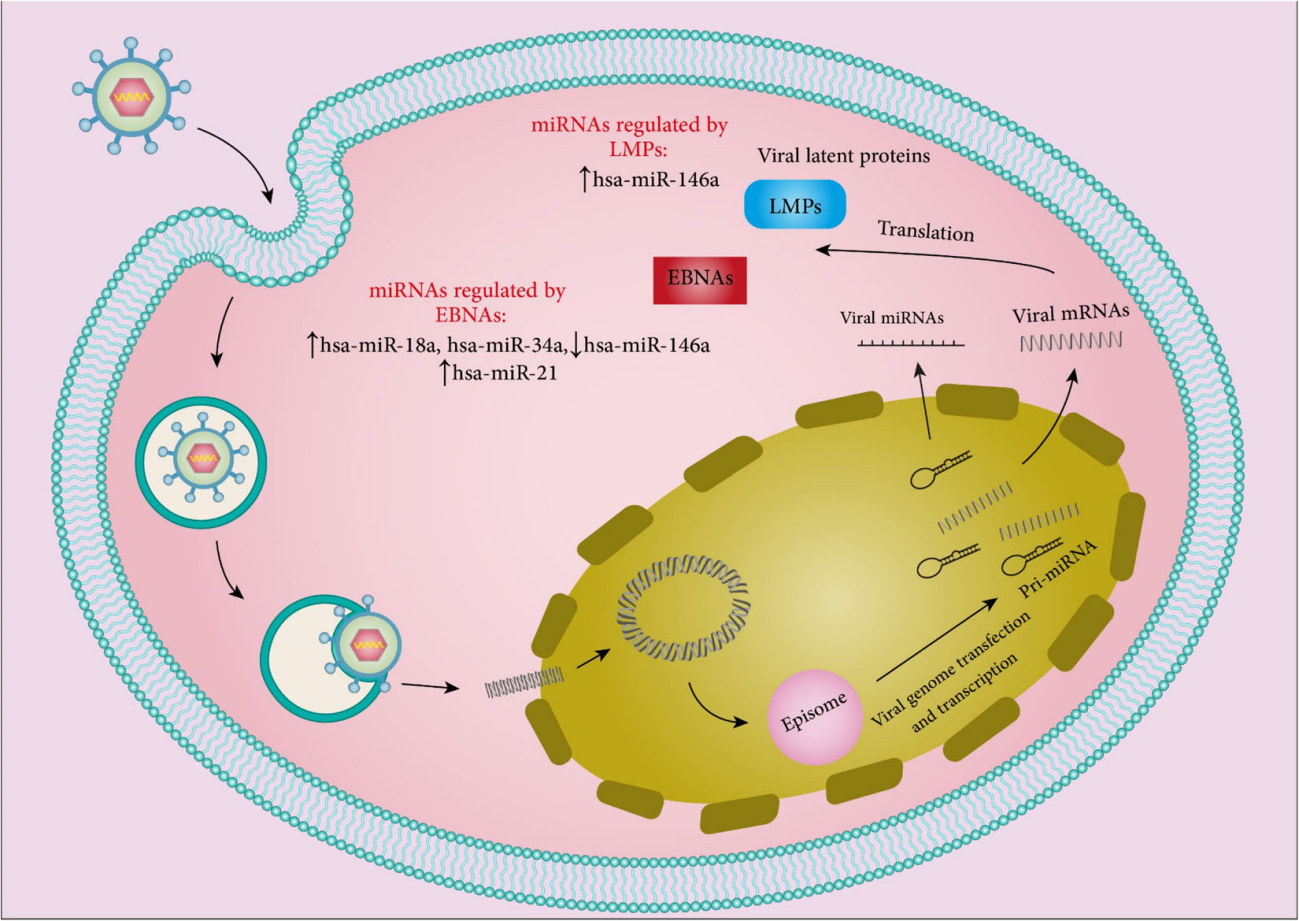
Cellular miRNAs involved in EBV infection. In EBV latency type III cell line, LMP1 induces miR‐146a expression. In lymphoma patients, the levels of miR‐18a are positively correlated with EBNA1. miR‐34a is reversely correlated with EBNA1 in EBVaGC. Also, EBNA2 downregulates miR‐146a and upregulates miR‐21 in B lymphoma cells. miRNA dysregulation is indicated by an up or down arrow. [Color figure can be viewed at wileyonlinelibrary.com]

In addition to the mentioned miRNAs, other various miRNAs are documented in Table 8 and summarized in Figure 17.

**Table 8:** Viral miRNAs and their contribution to EBV infection and related cancers.

miRNA	EBV‐infected cells	Target/human or virus	Effect	References
miR‐BHRF1‐2‐5p	Latently infected B cells	3′ UTR of IL‐1R1/Human	Downregulates protein and RNA levels of IL1R1, changes the levels of steady‐state cytokine. Inhibition of the miRNA induces the expression of pro‐inflammatory cytokine.	440
miR‐BART3‐3p	EBVaGC	TP53 gene/Human	Promotes the growth of gastric cancer cells in vitro and in vivo, downregulates p21 by targeting TP53 gene, prevents the senescence of gastric cancer cells.	441
miR‐BART6‐3p	Human PMBC, HK‐1, C666‐1, BJAB cells	3′ UTR of RIG‐I/Human	Inhibits IFN‐β signaling and production of IFN‐β mediated by RIG‐I‐like receptor signaling, represses EBV‐triggered immune response, facilitates EBV infection.	442
miR‐BART6‐3p	Burkitt lymphoma (BL)	PTEN and IL‐6 receptor (p80, gp130)/Human	Inhibition of the viral miRNA upregulates protein and mRNA levels of PTEN and IL‐6, has an important role in the pathogenesis of Burkitt lymphoma.	443
miR‐BART6	C666‐1 nasopharyngeal carcinoma cells	EBNA2/VirusDicer/Human	miRBART6‐5p RNAs regulate the production of host miRNA by targeting Dicer, suppressing miR‐BART6 upregulates EBNA2, Rta, and Zta.	444
miR‐BART8‐3p	Nasopharyngeal carcinoma (NPC)	PAG1/Human	Increases in patients with NPC, inhibits PAG1 host gene and promotes metastasis and invasion of radioresistant NPC cells.	445
miR‐BART9	NPC	E‐cadherin/Human	Induce a mesenchymal‐like phenotype by inhibiting E‐cadherin, increases the invasion and migration of NPC cells, function as a prometastatic viral miRNA.	446
miR‐BART10‐3p	NPC	3′UTR of ALK7/Human	Suppresses the expression of the ALK7, promotes dedifferentiation and proliferation of NPC.	447
miR‐BART11‐5p	HEK 293T	3′UTR of EBF1/Human	Targets EBF1, may play a role in the modulation of B‐cell differentiation.	448
miR‐BART11	NPC, GC	3′UTR of FOXP1/Human	Downregulates FOXP1 and its tumor‐suppressive effect, inhibits FOXP1‐induced differentiation of TAM, promotes proliferation and inflammation‐induced carcinogenesis of gastric cancer and nasopharyngeal carcinoma.	449
miR‐BART 15‐3p	EBVaGC	3′UTR of TAX1BP1/Human	Downregulates the protein and mRNA levels of TAX1BP1, regulates NF‐κB activity, promoteschemosensitivity to 5‐FU, increases apoptosis by targeting TAX1BP.	450
miR‐BART20‐5p	EBVaGC	3′UTR of BAD/Human	Downregulates the protein and mRNA levels of BAD, enhances cell growth by reducing apoptosis, increases chemoresistance to docetaxel and 5‐FU, contributes to tumorigenesis of EBVaGC.	451
miR‐BART‐22	NPC	LMP2A/Virus	Downregulate the expression of LMP2A leads to promotion of tumor cell survivor and escape from host immune system.	452

## 7 OTHER VIRUSES

### 7.1 Dengue virus

Dengue virus (DENV) as a member of the *Flaviviridae* family is a mosquito‐borne viral infection with the ability of establishing mild‐to‐severe life‐threatening diseases, including visceral disease, dengue shock syndrome (DSS), and dengue hemorrhagic fever (DHF). Severe infection can lead to cytokine storm and vascular leakage, hypotension, and shock.^453^ DENV‐infected patients have increased steadily over the past 70 years and more than 100 countries are struggling with the infection. With an estimated 390 million infections, each year DENV infection poses a risk to each 2.5 million people. Dengue virus has been classified into four serotypes including DENV‐1, DENV‐2, DENV‐3, and DENV‐4.^454^

**Figure 18:**
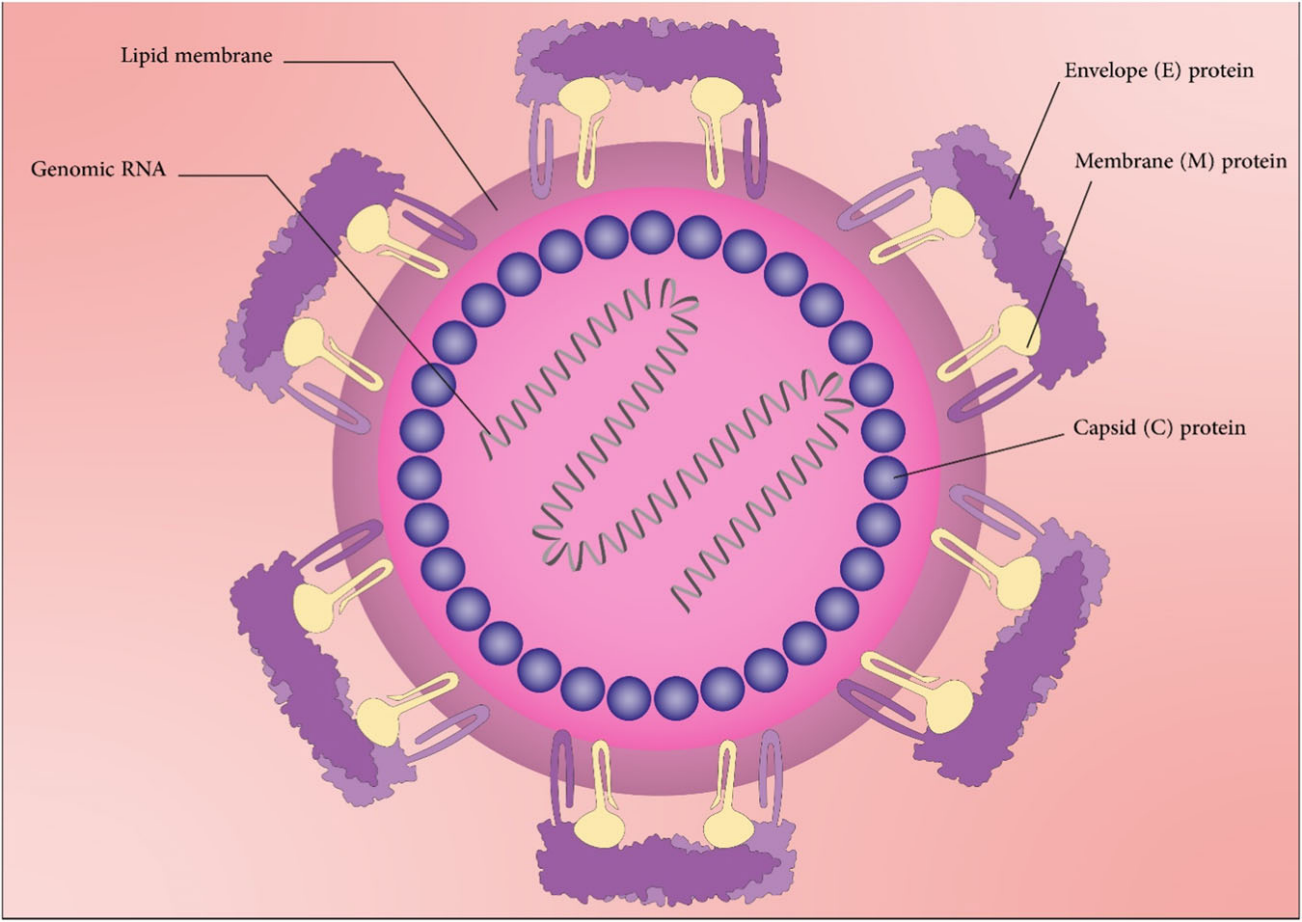
Schematic representation of DENV structure. [Color figure can be viewed at wileyonlinelibrary.com]

The genome of the virus is an ssRNA with positive‐sense and a length of 11 kb. Viral envelope protein covers the genome containing icosahedral nucleocapsid. The RNA genome of the virus consists of a long ORF with 5′‐ and 3′‐UTR in the upstream and downstream. One polypeptide encoded by the genome is cleaved by the host‐ and virus‐derived proteases into 10 different proteins. These proteins consist of seven nonstructural (NS) proteins (NS1, NS2A, NS2B, NS3, NS4A, NS4B, and NS5), and three structural proteins of C (capsid), prM/M (membrane), and E (envelope) (Figure 18).^455^ Structural proteins have different functions. For instance, NS3 has protease activity in its N‐terminal as well as an RNA triphosphatase domain and ATPase/helicase in its C‐terminal.^456^ Integral membrane proteins of NS2A, NS4A, NS4B, and NS2B are essential for viral replication. NS5 as the largest viral protein has a domain with RNA‐dependent RNA polymerase (RdRp) function in its C‐terminal and a methyltransferase domain (MTase) in its N‐terminal.^457^ NS1 interacts with prM and E proteins and plays a major role in particle production and viral assembly.^458^ Also, it binds to the lumen side of the ER membrane, forms a dimer and facilitates anchoring of the viral replication complex.^459^

The virus utilizes receptor‐based endocytosis for entrance into target cells; previous studies showed that DENV particles bind to uncharacterized cellular receptors and move along the cell surface before being captured.^460^, ^461^ Different serotypes of the DENV utilize different receptors including lectins, heparin sulfate, DC‐SIGN, the mannose receptor (MR) of macrophages, and heat‐shock proteins (HSP) 90 and 70. Furthermore, TIM‐1 and TAM, which enhance DENV infections are identified as important receptors in the viral entry.^462^, ^463^ The primary route for virus entrance is receptor‐mediated endocytosis and the virus is internalized with two endocytic pathways: (I) clathrin‐mediated endocytosis and (II) nonclassical clathrin‐independent endocytic pathways.^464^ DENV may use different entry routes based on its serotype. For instance, in Vero cells, DENV‐1 employs a clathrin‐dependent pathway while DENV‐2 prefers nonclassical clathrin‐ and caveolin‐independent pathway.^465^ Following endocytosis despite the selected route, E protein‐mediated membrane fusion releases the viral RNP into the cytoplasm, C protein may also have a role in this process.^466^, ^467^ Viral RNA and proteins are released into the cytoplasm after uncoating viral core. The rough ER is responsible for viral protein synthesis. ER‐associated ribosomes translate polyproteins from the single ORF of the positive viral RNA. As mentioned earlier, host and viral proteases cleavages this polyprotein into structural proteins and NSPs. The formation of vesicle packets (VPs) which include clusters of double‐membrane vesicles (Ve) is induced by viral proteins through invaginations of the ER. The genome replication complex is located in Ve.^468^ NS5 which functions as the RdRp transcribes the ds RNA from the viral positive strand. Furthermore, the intermediate negative RNA template which is used in subsequent genomic replication, is also translated from the positive strand. The negative strand transcribes multiple positive strands. ER assembly of the methylated positive strand, prM, E, and C proteins forms immature virion.^469^ During viral maturation, produced viral proteins and genome pack into virions in the ER and pass into Golgi vesicles. Host TGN protease furin produces M protein by cleaving the prM. This step is a required event in the maturation and replication cycle of the virion.^470^ The last step is releasing the matured virions by exocytosis. In addition to structural proteins, NSPs, including NS2A also play a role in viral assembly. NS2A is also involved in RNA replication.^471^

#### 7.1.1 Role of miRNAs in DENV infection

Similar to other viral infections, hsa‐miR‐155 has also a role in Dengue pathogenesis. DENV infection time‐dependently downregulates the expression of hsa‐miR‐155. In vivo overexpression of this miRNA reduced propagation and life‐threatening effects of the Dengue infection in ICR suckling mice. Bach1 inhibits the transcription of heme oxygenase‐1 (HO‐1). It was demonstrated that hsa‐miR‐155 inhibits the activity of the DENV by targeting 3′‐UTR of the Bach1 which leads to induction of HO‐1 regulated inhibition of the protease activity of the viral NS2B/NS3. Antiviral interferon responses such as OAS1 (2′‐5′‐oligoadenylate synthetase 1), 2, 3, and PKR (interferon‐induced protein kinase R) were induced as a result of inhibition of viral NS2B/NS3 protease activity which reduced replication of the DENV. Taken together, DENV infection induces downregulation of the hsa‐miR‐155 which is beneficial for DENV replication.^472^ DENV infection in macrophage‐monocytic cell line U937‐DC‐SIGN and hepatic Huh‐7 cells upregulated the expression of the hsa‐miR‐Let‐7c. Upregulated miRNA downregulates BACH1 and also downregulates infection of DENV2 and DENV4. HO‐1 expression was increased in infected cells when BACH1 expression was downregulated, possibly contributing to the stress oxidative response.^473^

DENV2 infection of human umbilical vein endothelial cells (HUVEC) decreases PPARγ expression which downregulates the expression of hsa‐miR‐573. This miRNA downregulates the expression of TLR2 and suppresses pro‐inflammatory IFN response. DENV2‐induced downregulation of the hsa‐miR‐573 can serve as a prognostic and therapeutic role for severe dengue patients.^474^

Wen et al. showed that hsa‐miR‐548g‐3p can affect DENV replication through direct targeting and binding to the Stem Loop A (SLA) promoter in the 5′‐UTR of DENV genome. The overexpression of the hsa‐miR‐548g‐3p led to inhibition of the DENV RNA and protein synthesis. Also, the cytopathic effect of DENV2 delayed as a result of the miRNA overexpression. It was demonstrated that the antiviral effect of this miRNA is not related to the interferon pathway and cell cycle arrest. Furthermore, knockdown of the miRNA significantly increased DENV RNA accumulation and promoted virus replication.^475^

In another study, it was demonstrated that DENV infection induces the upregulation of the hsa‐miR‐30e*. hsa‐miR‐30e* enhanced host antiviral immune response which resulted in suppressing the DENV infection. This miRNA inhibits the negative feedback loop of the NF‐κB/IκBα pathway by directly targeting of 3′‐UTR of the IκBα. This process results in the hyperactivation of NF‐κB. Furthermore, hsa‐miR‐30e* increases the expression of the IFN‐β. Also suppression of IκBα mediates the effect of miRNA on antiviral response induced by IFN‐β. Upregulation of the miRNA also leads to upregulated expression of the IFITM1, MxA, and OAS1 as downstream ISGs and suppressing of viral replication.^476^

**Figure 19:**
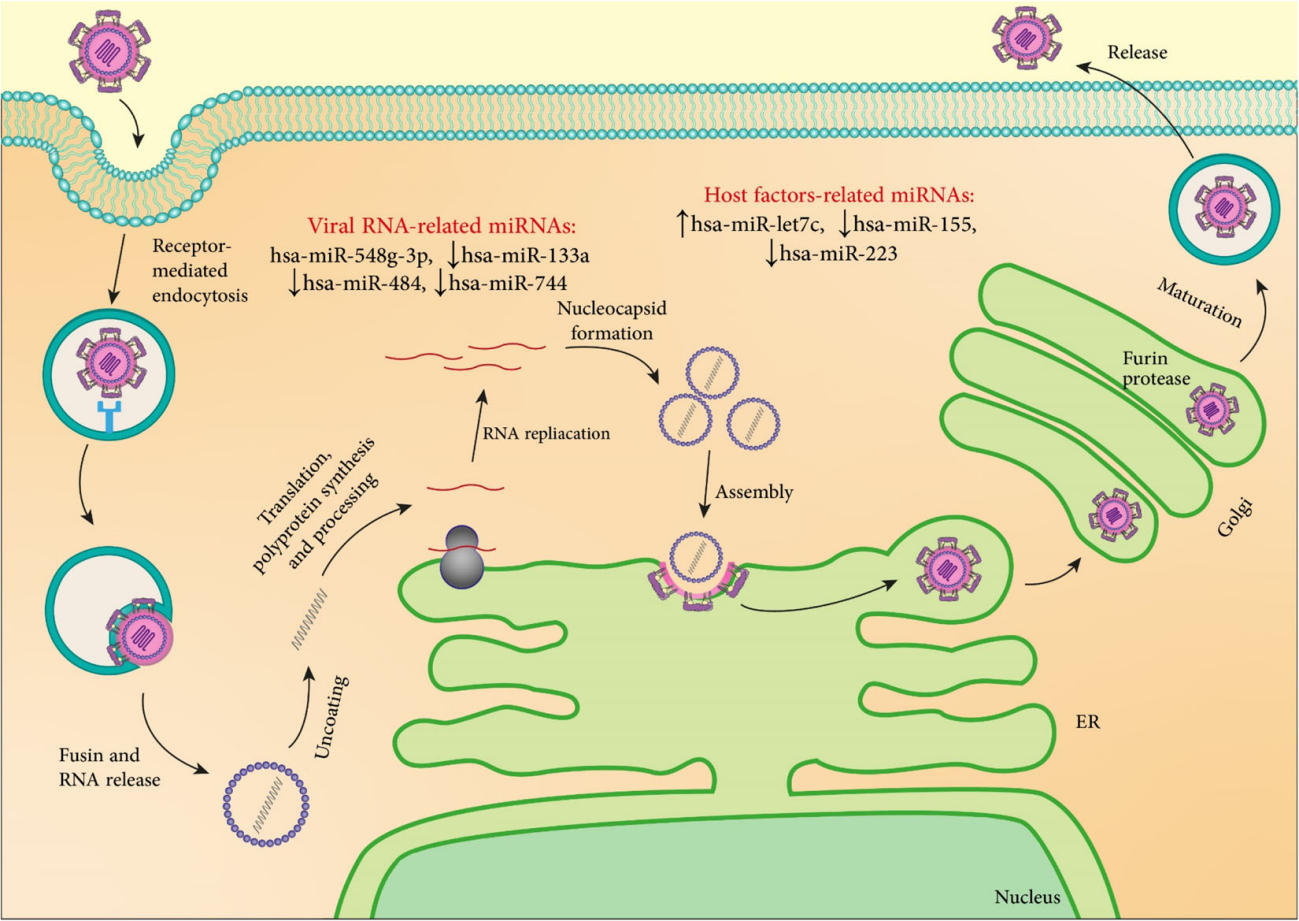
Summary of the miRNAs involved in DENV infection. hsa‐miR‐548g‐3p affects DENV replication through binding to the SLA promotor in the 5′‐UTR. hsa‐miR‐133a, hsa‐miR‐484, and hsa‐miR‐744 are downregulated by DENV 3′‐UTR. Other miRNAs including hsa‐Let‐7c, hsa‐miR‐155, and hsa‐miR‐223 modulate viral infection by regulating host factors. miRNA dysregulation is indicated by an up or down arrow. [Color figure can be viewed at wileyonlinelibrary.com]

A research in monocytic THP‐1 and primary human monocytes cells showed upregulation of hsa‐miR‐146a induced by DENV infection. This miRNA impaired IFN‐β in THP‐1 cells and reduced the expression of the type III IFNs. Dampening the IFN‐ β production promoted pro‐viral effect of the miRNA. On the other hand, hsa‐miR‐146a promoted viral activity by directly targeting the TRAF6. Inhibition of the TRAF6 reduced the type I IFN‐mediated antiviral defense. In addition, inhibition of this miRNA suppressed DENV2 replication which was restored by silencing of IFNAR1/2 and neutralization of IFN‐ β in suppressed hsa‐miR‐146a. These findings showed that the DENV‐induced upregulated hsa‐miR‐146a has a pro‐viral role in DENV infection.^477^ Table 9 highlights some important information about additional miRNAs, and Figure 19 summarizes the miRNAs involved in DENV infection.

**Table 9:** miRNAs and their contribution to DENV infection.

miRNA	Expression	Target/Human or virus	Effect	References
hsa‐miR‐133a	Downregulated	Polypyrimidine tract binding protein (PTB)/Human 3′ UTR of DENV RNA/Virus	Endogenous expression of the miRNA downregulates 3′ UTR of DENV, Overexpression of the miRNA inhibits DENV2 replication through suppressing of the PTB. In the early stages of infection, DENV1 to DENV4 downregulates miR‐133a expression.	478
hsa‐miR‐223	Downregulated	3′ UTR of the STMN1/Human	C/EBPα and E2F1 involves in downregulation of the hsa‐miR‐223 after DENV2 infection. Downregulation of the miRNA increases expression of the STMN1 and enhances DENV2 replication, overexpression of the miRNA inhibits DENV2 replication.	42
hsa‐miR‐484	Downregulated	3′ UTR of DENV RNA/Virus	hsa‐miR‐484 can be downregulated by DENV infection and DENV RNA 3′ UTR expression.	479
hsa‐miR‐744	Downregulated	3′ UTR of DENV RNA/Virus	hsa‐miR‐744 can be downregulated by DENV infection and DENV RNA3′ UTR expression. Overexpression of the miRNA inhibits DENV‐2 protein production and inhibits infection of the four DENV serotypes.	479
hsa‐miR‐150	Upregulated	EZH2/Human	The upregulation downregulates EZH2 and can act as a biomarker in the early stages of DENV infection.	480
hsa‐miR‐383‐5p	Upregulated	PLA2G4A/Human	Overexpression of the miRNA represses PLA2G4A which is important for the production of infectious DENV particles.	481
hsa‐miR‐3614‐5p	‐	ADAR1/Human	Upregulated in DENV‐negative cells, overexpression of the hsa‐miR‐3614‐5p reduces DENV infectivity and replication by targeting DENV proviral protein ADAR1.	482

### 7.2 Zika virus

Zika virus (ZIKV) as another member of the *Flaviviridae* family was first discovered in 1947, in the Zika Valley of Uganda.^483^ Based on the reports, from 2016 to March 2019, there were 81,852, 609, 1800, and 15 reported Zika cases, respectively.^484^ Similar to the DENV, ZIKV also is a mosquito‐transmitted virus. However, it can also be transmitted vertically, sexually, and through blood transfusions.^485^, ^486^, ^487^ ZIKV can cause febrile flu‐like illness and rash, which makes its symptoms similar to DENV infection. In addition, unlike most other flaviviruses, ZIKV infection can also cause Zika congenital syndrome (a group of neurological disorders in infants and fetuses) and Guillain‐Barré syndrome in adults. Adults who are infected may get paralysis.^488^, ^489^

**Figure 20:**
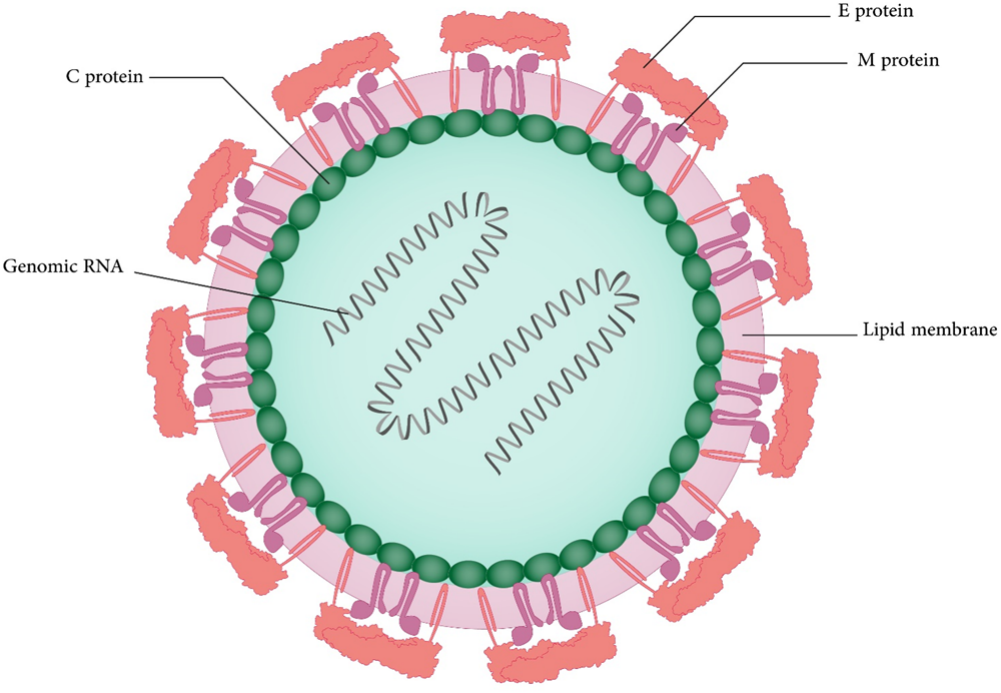
Schematic representation of ZIKV structure. [Color figure can be viewed at wileyonlinelibrary.com]

The structure of the ZIKV (Figure 20) is similar to DENV. The virus is composed of an enveloped, icosahedral virion with a 40–50 nm diameter. The genome of the virus is an 11 kb nonsegmented positive‐sense ssRNA.^490^ A single long ORF, and 5′ and 3′ noncoding regions of the genome translate a single polypeptide chain, which is cleaved to three structural proteins (precursor membrane (prM) protein, envelope (E) protein, and capsid (C) protein) and seven NSPs (NS1‐NS2A‐NS2B‐NS3‐NS4A‐NS4B‐NS5). Virus replication is driven by NSPs while structural proteins are responsible for building the new virus shell. The E protein, located on top of the M protein, is the main protein involved in host immune recognition and receptor binding. It also facilitates the cytoplasmic release of the viral RNA and fusion with the endosomal membrane.^491^ There are 180 copies of prM‐E heterodimers in the icosahedral surface of the immature ZIKV.^489^ The E protein contains the following domains: domain I, domain II, and domain III.^492^ Trimers of the prM–E complex form the spikes of the immature ZIKV, which rearranges into prM–E dimers after the maturation process.^469^, ^493^

**Figure 21:**
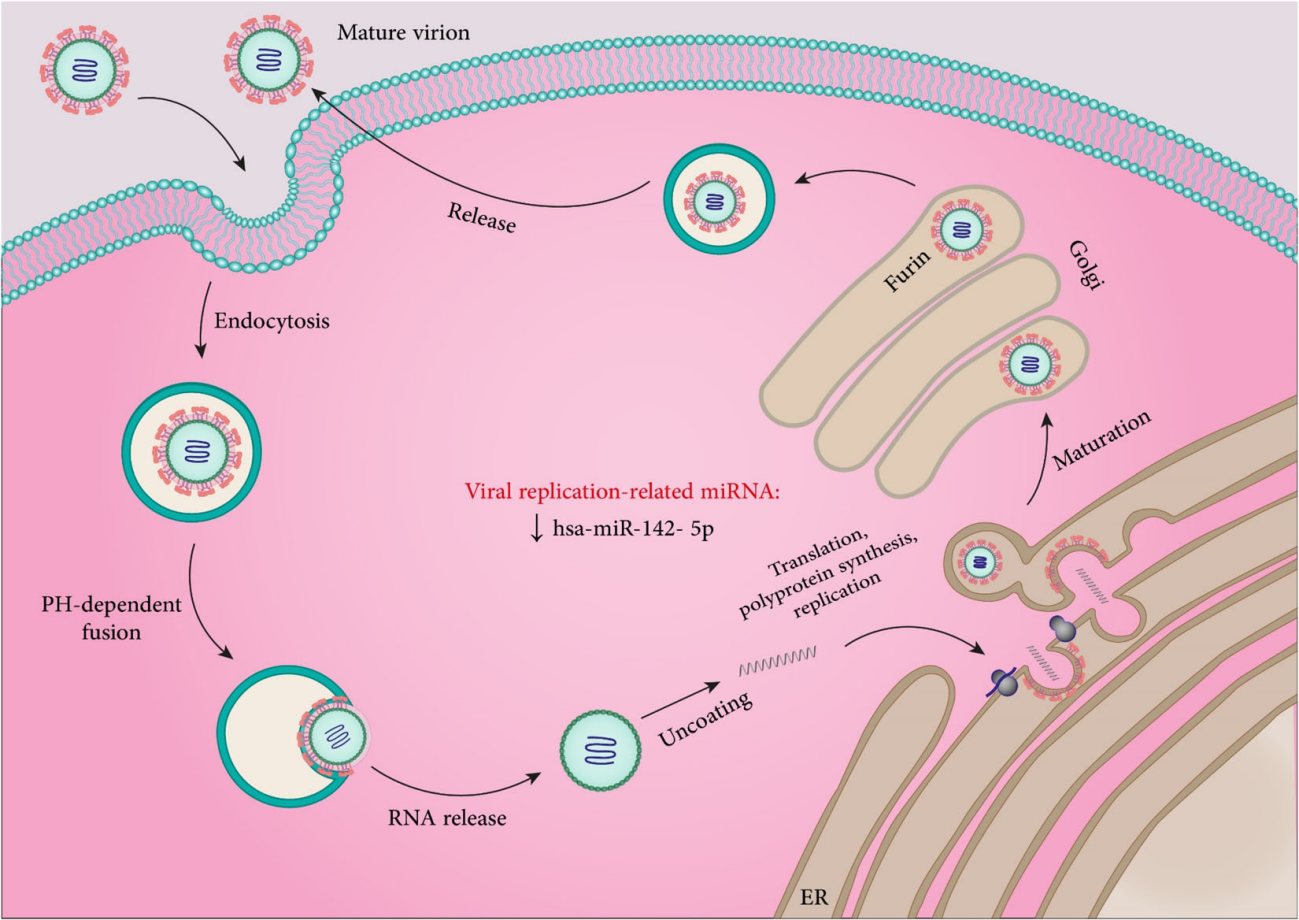
The replication cycle of the ZIKV virus and involved miRNA. hsa‐miR‐142‐5p is downregulated by ZIKV infection and its upregulation inhibits viral replication. [Color figure can be viewed at wileyonlinelibrary.com]

The life cycle of the ZIKV (Figure 21) is similar to DENV which was explained earlier. Zika entry is permitted by the entry factors including TAM receptor AXL, TIM‐1, Tyro3, AXL, and DC‐SIGN.^494^ Briefly, the replication cycle of the virus initiates with the binding of the viral E protein to the host receptor which results in endocytosis. The viral envelope fuses with the host membrane due to the pH of the endosome which leads to a conformational change of the E protein and triggers the fusion.^495^ The ER translates a polyprotein from the viral genome, then is cleaved by the host and viral proteins into structural proteins and NSPs. NS2B–NS3 as the viral protease is the most conserved protease among ZIKV strains.^496^ The RdRp utilizes positive‐strand viruses as templates and synthesizes negative‐strand viral RNA following the release of their positive‐strand genomes into the cytoplasm. On the ER, viral mRNAs and new viral positive ssRNA strands synthesize from the negative strand. The host machinery translates these into viral polyproteins which then cleaves by viral protease to structural proteins and NSPs.^497^ Vesicle packages and the surface of the ER are the sites of genomic replication and virus assembly, respectively. Virion maturation occurs in the *trans*‐Golgi network by exploiting the host secretion system, and matured virion is released by exocytosis^498^

#### 7.2.1 Role of miRNAs in ZIKV infection

Like other viral infections, ZIKV infection also modulates host miRNA expression. Examining expression patterns of 599 miRNAs showed that ZIKV infection of the neurons downregulates miRNA processing enzymes. It also significantly upregulates hsa‐miR‐29a, hsa‐miR‐29b, hsa‐miR‐155, hsa‐miR‐203, and downregulates hsa‐miR‐124‐3p. These miRNAs which are regulated by ZIKV infection also play a role in the infection of the other flaviviruses.^499^

In a study performed by Seong et al. in ZIKV‐infected human umbilical cord mesenchymal stem cells (hUCMSCs), it has been found that ZIKV infections downregulates the expression of hsa‐miR‐142‐5p and overexpression of the miRNA inhibits viral replication. Decreased levels of hsa‐miR‐142‐5p led to upregulated expression of its targets such as cellular ITGAV and IL6ST. ITGAV has a role in signaling and cell surface adhesion while IL6ST modulates myocyte apoptosis. ITGAV and IL6ST, in turn, reduced the expression of STAT3 and affected JAK/STAT signaling pathway. On the other hand, overexpression of hsa‐miR‐142‐5p in A549 cells suppressed the replication of the ZIKV through decreasing gene expression of the viral NS5. Furthermore, its overexpression in A549 cells reduced the mRNA expression of the ITGAV and IL6ST, indicating the regulatory role of the hsa‐miR‐142‐5p in ZIKV infection.^500^

In another study in microglial cells, it was demonstrated that ZIKV‐N1 suppresses the ROS activity and induces the expression of hsa‐miR‐146a. Upregulation of the miRNA downregulates the expression of STAT‐1 and TRAF6 which suppresses pNF‐κBp65 and TNF‐α downstream. This study showed the role of the ZIKV‐NS1 in suppressing cellular antiviral and pro‐inflammatory responses through regulation of the hsa‐miR‐146a.^501^

ZIKV infection in A549 cells highly induces expression of the hsa‐miR‐103a‐3p. ZIKA infection activates the p38 MAPK signaling pathway which results in stimulation of the ZIKV replication. OTUD4 is a target of the miR‐103a‐3p and its overexpression decreases ZIKV replication and inhibits p38 MAPK signaling pathway. ZIKV‐induced upregulation of the miRNA promotes replication of the ZIKV through targeting of OTUD4.^502^

In ZIKV‐infected glioblastoma cells, ZIKV infection enhances the expression of the hsa‐miR‐34c. As a result of miRNA overexpression, the sphere formation, glioblastoma cell growth, NUMB and BCL2 protein expressions are reduced. NUMB functions in the development of the normal central nervous system. ZIKV infection causing an apoptotic response by sub G0 induction increases the death of the glioblastoma cells. Totally, BCL2 inhibition by ZIKV could increase the effect of chemo/radiotherapies which makes ZIKV a great oncolytic virus in glioblastoma treatment.^503^

Analyzing the miRNA patterns in ZIKV‐infected human neural stem cells (hNSCs) introduced hsa‐miR‐124‐3p and hsa‐miR‐let‐7c as other miRNAs regulated by the viral infection. ZIKV upregulated let‐7c and slightly increased the expression of the hsa‐miR‐123‐3p. Both miRNAs were identified to have a role in microcephaly. Also, hsa‐miR‐124‐3p was identified to have a role in the posttranscriptional regulation of the infected cells. High‐mobility group AT‐hook 2 (HMGA2), as a target gene of the let‐7c, was downregulated following let‐7c overexpression in ZIKV infection. A target gene related to ZIKV pathogenesis with functions in neural stem cells has not been identified for the hsa‐miR‐124‐3p. However, transferrin receptor (TFRC) was predicted as the potential target gene of the hsa‐miR‐124‐3p. ZIKV infection of hNSCs decreased mRNA levels of the TFRC.^504^

In another study, the expression of the hsa‐miR‐9 was evaluated in the developing and adult mouse CN. ZIKV infection in the cortex upregulated the expression of the hsa‐miR‐9 which in turn led to increased neuronal apoptosis and decreased neurogenesis. Besides, the expression of GDNF, a specific target of the hsa‐miR‐9, was reduced following ZIKV infection. GDNF protects neurons from apoptosis. Taken together, ZIKV infection may result in microcephaly through decreasing neurogenesis and the neural progenitor population.^505^

miRNA biogenesis mediated by the Dicer is essential for embryonic brain development. Zeng et al. demonstrated that ZIKV capsid can suppress Dicer activity and hence miRNA biogenesis by mapping the ZIKV‐host interaction in neural stem cells (NSCs). ZIKV evasion from host RNAi machinery can be facilitated through the ability of the capsid protein of the ZIKV in hijacking Dicer which results in blocking of the cortical development and neurogenesis. The viral capsid strongly binds to the Dicer and also targets the linker domain between the two functional RNase III domains. The evidence showed that the histidine on position 41 of the capsid is responsible for Capsid‐Dicer interaction and a single mutation in H41R amino acid eliminated this interaction. The capsid protein of the other flaviviruses does not have the ability to block the Dicer activity. The study suggested that capsid modulation of the Dicer may increase viral pathogenesis and replication.^506^

### 7.3 Ebola virus

Ebola virus (EBOV) is a member of the *Filoviridae* family and categorizes into five species: *Bundibugyo ebolavirus* (Bundibugyo virus, BDBV), Reston ebolavirus (Reston virus, RESTV), *Täı Forest ebolavirus* (Taï Forest virus, TAFV), *Sudan ebolavirus* (Sudan virus, SUDV), *and Zaire ebolavirus* (Ebola virus, EBOV). Ebola virus and Sudan ebolavirus are responsible for 40%–90% of Ebola outbreaks and they are more pathogenic than others. The first reported case of Ebola virus disease (EVD) also known as Ebola hemorrhagic fever caused by EBOVs was in 1976.^507^ As one of the deadliest viruses, the EBOV outbreak in the Democratic Republic of the Congo (2018–2020) and West Africa between 2013 and 2016 led to the death of more than 2200 and 11,000 deaths, respectively.^508^, ^509^ The transmission routes of the EBOV are via direct contact with infectious body fluids of EDV patients, such as blood, semen, saliva, and tears.^510^

**Figure 22:**
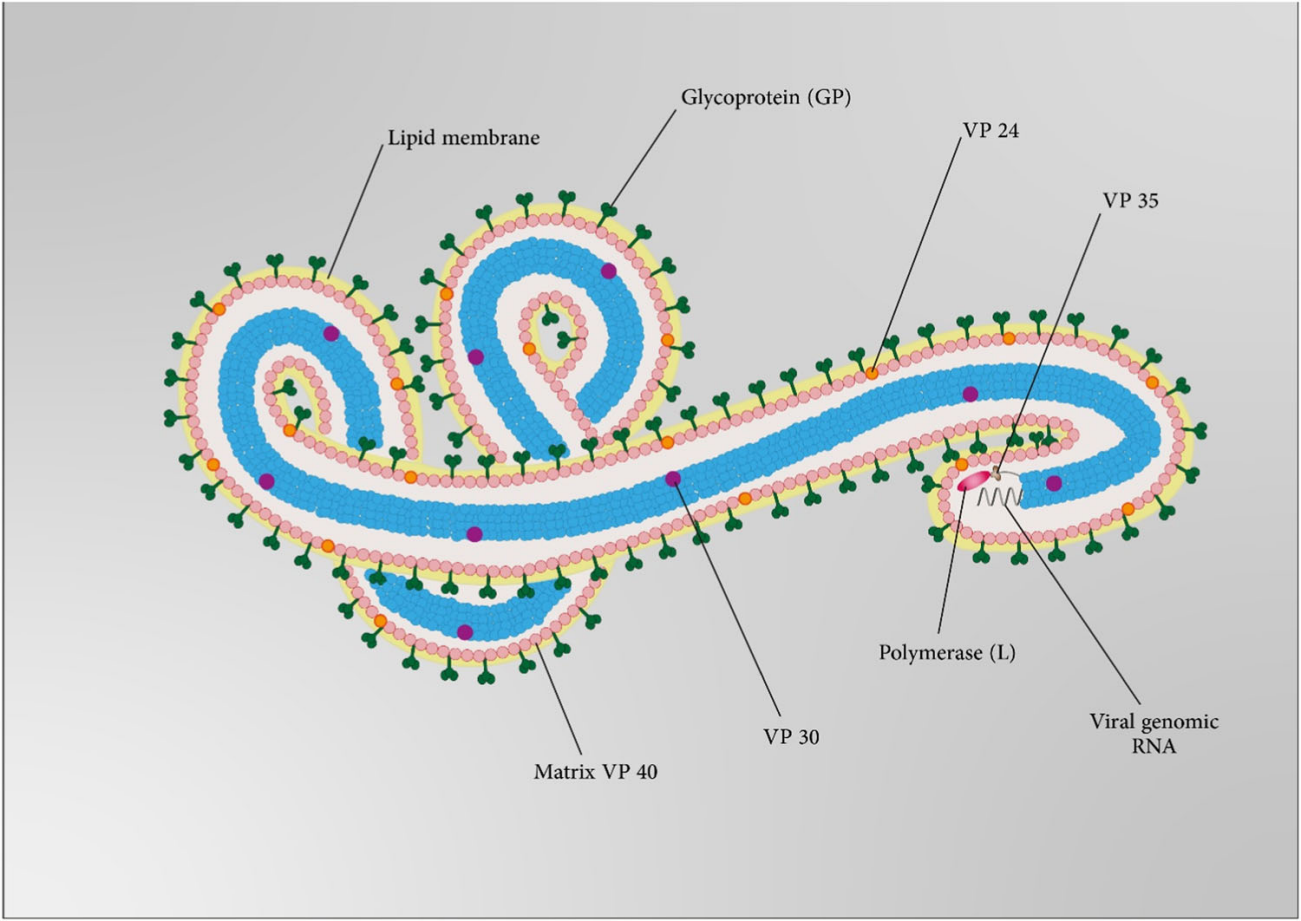
Schematic representation of EBOV structure. [Color figure can be viewed at wileyonlinelibrary.com]

**Figure 23:**
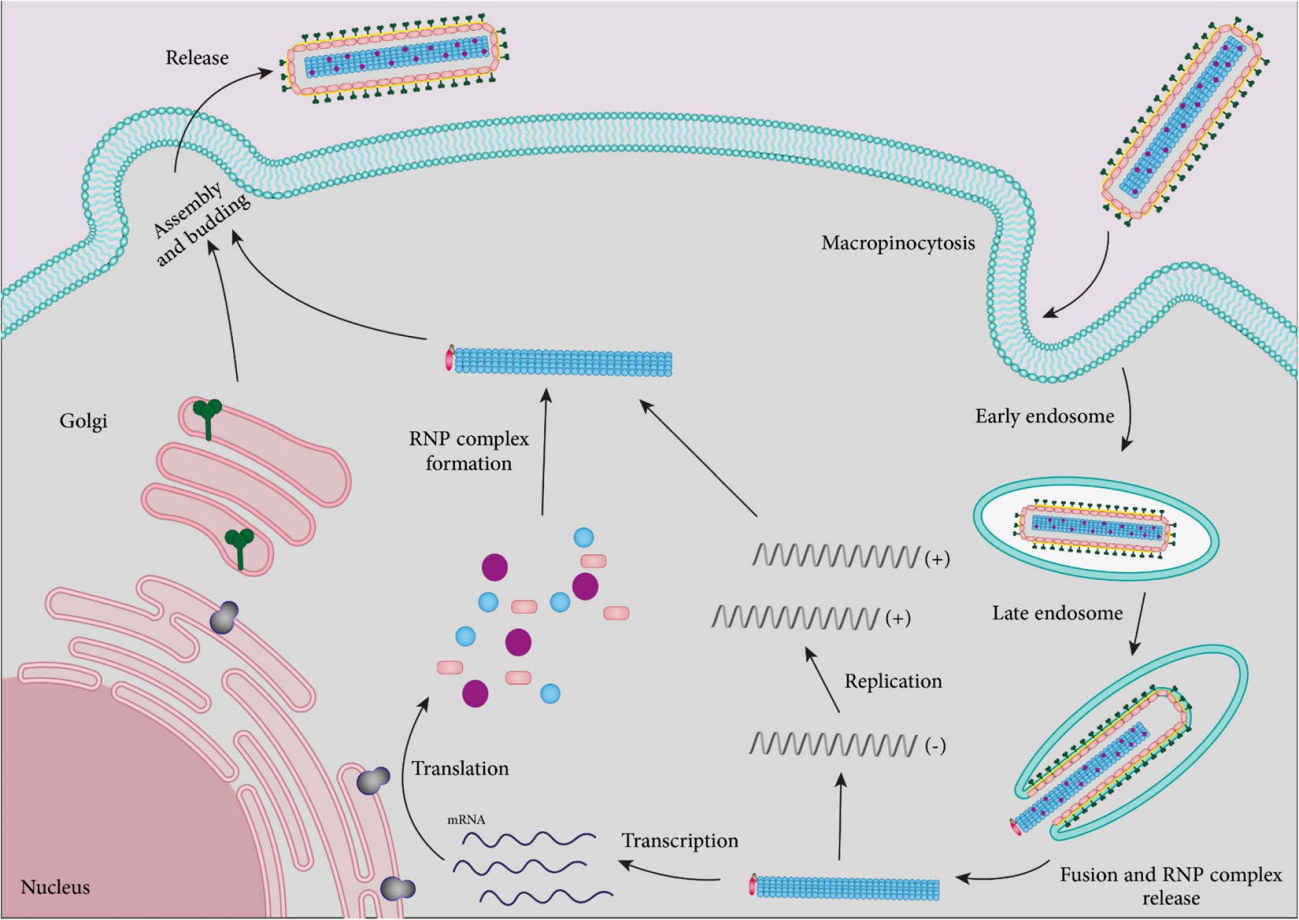
The replication cycle of the EBOV virus. [Color figure can be viewed at wileyonlinelibrary.com]

The viral genome is a 19 kb nonsegmented negative‐strand RNA located in nucleocapsid which encodes seven viral proteins including, RNA‐dependent RNA polymerase (L), VP24, VP30, VP35, VP40, NP, GP (glycoprotein), and soluble glycoprotein (sGP). Nucleoprotein (NP) encapsidates the viral genome. VP30 as a transcriptional activator, VP35 as the polymerase cofactor, and the L polymerase is associated with the NP protein.^511^ Also, VP35 has a role in the formation of the viral RNA synthesis complex by linking NPs with viral RNA synthesis complex.^512^ VP24 and VP40 as a major matrix proteins play a role in replication, transcription, assembly, and budding.^513^ VP40 as the matrix protein surrounds nucleocapsid (Figure 22).^514^ Viral entry is regulated by the viral surface GP spikes, which mediate binding to host cells, endosomal entry, and membrane fusion.^515^ The GP protein is cleaved into GP1 and GP2 disulfide‐linked proteins by cellular cathepsins. The responsibility of the GP1 is interacting with one or more cellular receptors, while GP2 is essential for membrane fusion due to the structural fusion loop.^516^ EBOV infects cells by binding to cellular molecules including lectin‐type molecules. The entry mechanism is more macropinocytosis and less clathrin‐mediated endocytosis.^517^ Following EBOV entry by endocytic pathways, viral membrane is uncoated and fussed with the endolysosomal membranes through cleavages of the GP1,2 by host cathepsins which form fusion‐active form to interact with the host NPC1 receptor.^518^ The cytoplasm is the site of primary transcription. In this way, the viral ribonucleoprotein complex (RNP), which combined with genome RNA and polymerase performs the most crucial function in the viral proliferation cycle, is formed by the nucleocapsid protein (NP), which facilitates genomic RNA encapsidation.^519^ The released RNP facilitates the transcription and functions as a template to form the genomic RNA of the virions.^520^ Host ribosomes translate viral mRNAs into proteins which facilitate secondary transcription and genome replication. Viral proteins including VP24, GP, NP, and VP40 play different roles in assembly and budding. VP40 coordinates the virus assembly at the plasma membrane and maturation of the EBOV virion (Figure 23).^521^

#### 7.3.1 Role of miRNAs in EBOV infection

Similar to other viral infections, EBOV can modify the expression of the miRNAs. Zaire ebolavirus infection of the human retinal pigment epithelial cells in human ARPE‐19 RPE cells leads to upregulation of the hsa‐miR‐29b‐3p, hsa‐miR‐1307‐5p, and hsa‐miR‐33a‐5p as well as downregulation of hsa‐miR‐1307‐5p and hsa‐miR‐3074‐3p. Furthermore, infected RPE cells were also found to express viral EBOV‐miR‐1‐5p which is analog of the hsa‐miR‐155‐5p.^522^ Zebov‐miR‐1‐5p is analog of the hsa‐miR‐155‐5p. In EBOV infection, Zebov‐miR‐1‐5p reduces the expression of the importin‐a5 (KPNA1), a key mediator in terms of EBOV pathogenicity. This regulation results in higher mortality of the infected patients by inhibiting the function of IFN signaling pathway.^523^

The biology of the EBOV and inhibitory effect of the anti‐Ebola miRNAs were studied by a transcription‐ and replication‐competent virus‐like particle (trVLP) system. Three miRNAs including hsa‐miR‐15a‐3p, hsa‐miR‐103b, and hsa‐miR‐150‐3p showed inhibitory effects on trVLP reproduction. Among these miRNAs, the coding regions of VP40 and GP were directly targeted by the hsa‐miR‐150‐3p. miRNA‐induced regulation of these viral proteins inhibited the reproduction of trVLPs.^524^

Sheng et al. showed that EBOV glycoprotein‐expressing adenovirus infection of the HUVECs induced the expression of hsa‐miR‐196b‐5p, hsa‐miR‐320a, and hsa‐miR‐1246. Increased expression of these miRNAs downregulated their target genes with function in cell adhesion: caspase 8 and FADD‐like apoptosis regulator (CFLAR), dystroglycan1 (DAG1), and tissue factor pathway inhibitor (TFPI) which have a role in occurring of the highly lethal hemorrhagic fever. Overexpression of CFLAR, DAG1, and TFPI or inhibition of mentioned miRNAs reduced EBOV GP‐induced cell viability, emphasizing their therapeutic role in EBOV infection.^525^

In addition to host miRNAs, the expression of some viral miRNA‐like fragments was reported in EBOV infection. In a study, they showed the persistence of miR‐VP‐3p as a putative miRNA‐like RNA fragment in the serum of the EVD patients. miR‐VP‐3p is highly conserved among EBOV strains and was also present in the exosomal fractions.^526^ Furthermore, utilizing computational prediction followed by experimental validation resulted in identifying EBOV‐pre‐miR‐2 and EBOV‐pre‐miR‐1 miRNA precursors which are also conserved among different EBOV strains.^527^

## 8 CONCLUSION AND FUTURE PERSPECTIVE

Millions of people around the world are infected by viruses, which are important human pathogens. According to various studies, different host cellular and molecular pathways are involved in the pathogenesis of viral infections. Host cells can be affected by viruses in different ways, including regulating gene and miRNA expression. miRNAs as small RNA molecules and important components of host defense, play a functional role in viral infections. Host miRNAs can play a pro‐viral or antiviral role by affecting virus attachment to the host, propagation, and replication. Virus‐induced modulation and dysregulation of the miRNA levels can increase viral replication, pathogenies, and survival. Also, viral infection‐induced changes in miRNA levels are a key step in host response to infection which can increase or decrease host immune responses and can also lead to abnormal immune responses. miRNA expression can also increase or decrease the viral gene expression which also can affect viral replication.

In addition to host miRNAs, some viruses including HBV, IAV, HIV, HPV, HSV, EBV, and EBOV produce their own miRNAs (v‐miRNAs). They produce viral miRNAs to escape the host immune system or facilitate their replication cycle and infection. These v‐miRNAs can regulate viral and host gene expression. Both host and virus‐induced miRNAs can be beneficial to hosts or viruses by positively or negatively regulating the viral replication cycle.

The specificity and sensitivity of some miRNAs can be used as biomarkers and to diagnose viral infections in early stages Specific therapeutic methods can be achieved by direct targeting of these miRNAs. However, the changed expression of some miRNAs may not be related to viral infections and pathogenesis and may be cell or tissue‐specific. Also, in some viral infections in vivo studies are limited which needs to be improved. The knowledge toward the interaction between miRNAs and viral infections and involved molecular pathways is not fully understood. In addition, the biogenesis and function of viral miRNAs are also not clear. Further studies can be conducted on the regulation of the viral miRNAs and the impact of these regulations on the establishment and replication of viruses. Also, more studies should be conducted in the field of targeting viral miRNAs and the results of targeting viral miRNAs. Furthermore, the development of efficient delivery strategies for miRNA‐targeting therapies can facilitate the use of miRNAs in therapeutic fields, especially in the treatment of viral infections. Understanding more miRNAs involved in viral infections, their roles, involved genes, and identifying more viral miRNAs can facilitate finding therapies for targeting these miRNAs to cure viral‐induced infections and diseases.
